# Multicenter Longitudinal Quality Assessment of MS-Based Proteomics in Plasma and Serum

**DOI:** 10.1021/acs.jproteome.4c00644

**Published:** 2025-02-07

**Authors:** Oliver Kardell, Thomas Gronauer, Christine von Toerne, Juliane Merl-Pham, Ann-Christine König, Teresa K. Barth, Julia Mergner, Christina Ludwig, Johanna Tüshaus, Pieter Giesbertz, Stephan Breimann, Lisa Schweizer, Torsten Müller, Georg Kliewer, Ute Distler, David Gomez-Zepeda, Oliver Popp, Di Qin, Daniel Teupser, Jürgen Cox, Axel Imhof, Bernhard Küster, Stefan F. Lichtenthaler, Jeroen Krijgsveld, Stefan Tenzer, Philipp Mertins, Fabian Coscia, Stefanie M. Hauck

**Affiliations:** † Metabolomics and Proteomics Core, Helmholtz Zentrum München, German Research Center for Environmental Health, Munich 80939, Germany; ‡ Clinical Protein Analysis Unit (ClinZfP), Biomedical Center, Faculty of Medicine, LMU Munich, 82152 Martinsried, Germany; § Bavarian Center for Biomolecular Mass Spectrometry at Klinikum rechts der Isar (BayBioMS@MRI), Technical University of Munich, 80333 Munich, Germany; ∥ Bavarian Center for Biomolecular Mass Spectrometry (BayBioMS), School of Life Sciences, Technical University of Munich, 85354 Freising, Germany; ⊥ German Center for Neurodegenerative Diseases (DZNE) Munich, DZNE, Munich 81377, Germany; # Department of Proteomics and Signal Transduction, Max-Planck Institute of Biochemistry, Martinsried 82152, Germany; †† German Cancer Research Center (DKFZ), 69120 Heidelberg, Germany; ‡‡ Medical Faculty, Heidelberg University, 69120 Heidelberg, Germany; §§ Institute for Immunology, University Medical Center of the Johannes Gutenberg University Mainz, Mainz 55131, Germany; ∥∥ Immunoproteomics Unit, Helmholtz-Institute for Translational Oncology (HI-TRON) Mainz, 55131 Mainz, Germany; ⊥⊥ Max-Delbrück-Center for Molecular Medicine in the Helmholtz Association (MDC), 13125 Berlin, Germany; ## Institute of Laboratory Medicine, University Hospital, LMU Munich, 81377 Munich, Germany; ††† Computational Systems Biochemistry Research Group, Max-Planck Institute of Biochemistry, Martinsried 82152, Germany; ‡‡‡ Proteomics and Bioanalytics, School of Life Sciences, Technical University of Munich, 85354 Freising, Germany; §§§ Neuroproteomics, School of Medicine and Health, Klinikum rechts der Isar, Technical University of Munich, 81675 Munich, Germany; ∥∥∥ Munich Cluster for Systems Neurology (SyNergy), 81377 Munich, Germany

**Keywords:** longitudinal round-robin study, clinical specimen, plasma, serum, LC-MS/MS, R package mpwR

## Abstract

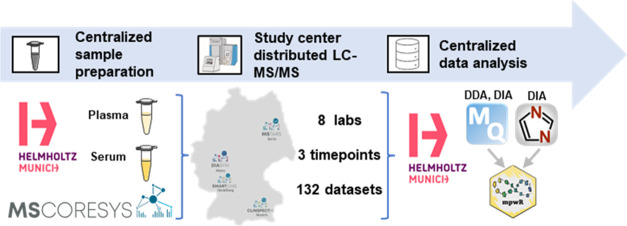

Advancing MS-based proteomics toward clinical applications evolves around developing standardized start-to-finish and fit-for-purpose workflows for clinical specimens. Steps along the method design involve the determination and optimization of several bioanalytical parameters such as selectivity, sensitivity, accuracy, and precision. In a joint effort, eight proteomics laboratories belonging to the MSCoreSys initiative including the CLINSPECT-M, MSTARS, DIASyM, and SMART-CARE consortia performed a longitudinal round-robin study to assess the analysis performance of plasma and serum as clinically relevant samples. A variety of LC-MS/MS setups including mass spectrometer models from ThermoFisher and Bruker as well as LC systems from ThermoFisher, Evosep, and Waters Corporation were used in this study. As key performance indicators, sensitivity, precision, and reproducibility were monitored over time. Protein identifications range between 300 and 400 IDs across different state-of-the-art MS instruments, with timsTOF Pro, Orbitrap Exploris 480, and Q Exactive HF-X being among the top performers. Overall, 71 proteins are reproducibly detectable in all setups in both serum and plasma samples, and 22 of these proteins are FDA-approved biomarkers, which are reproducibly quantified (CV < 20% with label-free quantification). In total, the round-robin study highlights a promising baseline for bringing MS-based measurements of serum and plasma samples closer to clinical utility.

## 1 Introduction

Clinical proteomics is a rapidly growing research area, which aims at analyzing protein profiles in clinical samples such as blood, urine, or tissue biopsies to decipher pathogenic mechanisms and ultimately to aid in understanding, diagnosis and treatment of diseases.^1^ Mass spectrometry (MS) is one of the key proteomics technologies with the potential to use clinical knowledge for developing new diagnostic methods, targets for drug development as well as expand knowledge of the underlying disease mechanisms. The successful implementation of MS-based assays in a clinical setting requires the establishment of standardized start-to-finish and fit-for-purpose workflows with a strong emphasis on achieving high levels of accuracy, reproducibility, and efficient handling of large sample numbers.^2^ In particular, the ability to consistently detect and measure proteins across large cohorts including measurements conducted at different time points is a decisive element in this process. Several multicenter studies have been performed to assess repeatability as well as intra- and interlaboratory reproducibility of MS-based proteomics measurements for both data-dependent (DDA) and data-independent acquisition (DIA) methods.^3−6^

In 2010 Tabb et al. evaluated the repeatability and reproducibility of peptide and protein identifications by LC-MS/MS acquired with DDA. The study included interlaboratory data sets from the NCI Clinical Proteomic Technology Assessment for Cancer. In detail, 144 LC-MS/MS experiments measured on Thermo LTQ and Orbitrap instruments were incorporated, and as samples yeast lysate, the NCI-20 defined dynamic range protein mix, and the Sigma UPS 1 defined equimolar protein mix were used. The findings showed higher repeatability and reproducibility for proteins compared to peptides, as well as a lower reproducibility between instruments of the same type compared to the repeatability of technical replicates on a single instrument.^4^

Furthermore, in 2017 Collins et al. conducted an interlaboratory study at 11 sites worldwide showing that DIA-based LC-MS/MS, in particular SWATH-MS, can consistently detect and reproducibly quantify over 4000 proteins in HEK 293 cells. The study demonstrated that reproducible quantitative proteomics data can be obtained across multiple laboratories and further highlighted the potential of SWATH-MS as reliable method for large-scale protein quantification.^5^ Additionally, Poulos et al. performed a comprehensive evaluation of reproducibility across over 1500 DIA-MS runs including pooled human cancer specimens on six Sciex QTOF instruments. First, the performance was monitored over a period of 4 month and subsequently the data was used to develop novel methods for data normalization and missing value imputation to pave a way toward large-scale quantitative proteomic studies.^6^ On top of that, benchmark studies were carried out to accurately evaluate the performance of DIA-methods and respective data analysis pipelines, which additionally advance the pursuit of bringing MS-based proteomics closer to clinical usefulness.^7,8^

Considering the type of samples involved in clinical routine, blood is commonly used, as it is easily accessible and can provide valuable information about the overall health of an individual. Blood tests can be used to measure a wide range of proteins, including enzymes, hormones, and markers of disease. Furthermore, both serum and plasma offer a low invasive source of valuable biological information and thus the clinical use provides a cost-effective and convenient way to monitor a patient’s health status.^9,10^ In MS-based proteomics the high dynamic range of these body fluids and the fact that high abundant proteins obscure low-abundant ones is known to impair the overall identification rate. For example, in plasma, Albumin alone makes up 50% and the top 22 proteins combined make up 99% of plasma proteins by weight.^11^ However, several techniques have been developed to overcome these limiting factors, making identifications of over 4000 proteins in plasma possible.^12,13^ Nevertheless, clinical translation is still pending, and Ignjatovic et al. even pointed out a tendency in proteomic plasma studies of prioritizing the aim to detect the largest number of proteins possible over the objective to focus on proteins that can be consistently detected and demonstrate a clinical utility.^11^ For example, Geyer et al. proposed a strategy termed “plasma protein profiling” in which large cohorts of plasma proteomes are analyzed at the greatest possible depth with streamlined and high-throughput technologies in contrast to measuring a small number of samples and controls in great depth with relatively low-throughput methods.^9,10^

The CLINSPECT-M consortium together with its MSCoreSys partner sites including the MSTARS, DIASyM, and SMART-CARE consortia and their respective proteomics laboratories initiated a longitudinal round-robin study to evaluate the performance of measuring clinically relevant samples such as plasma and serum over time and to monitor intra-, as well as interlaboratory reproducibility. Key performance indicators included number of identifications (IDs), data completeness (DC), as well as quantitative precision. In addition, special focus was on highlighting proteins consistently detected on each platform across all sites to access a baseline for detectable plasma and serum proteins, which were acquired without any enrichment, depletion, or fractionation workflow. In total, eight proteomic study centers distributed over Germany received proteolyzed plasma and serum samples ready for MS injection and measured each clinical specimen on their respective LC-MS/MS platforms including DDA- and/or DIA-based methods at three time points spanning over 9–12 months. Only the injection amount was standardized. No additional guidelines, protocols, or restrictions were placed on the measurements. All generated raw data were analyzed centrally by a common pipeline using MaxQuant as software and the R package mpwR.^14−16^ DIA data was additionally processed with DIA-NN.^17^

## 2 Experimental Procedure

### 2.1 Study Design

Pooled samples of plasma and serum were obtained from anonymized leftover material of clinical patient diagnostics at the Institute of Laboratory Medicine, LMU Hospital, LMU Munich. The Ethics Committee of the Medical Faculty of LMU Munich has provided a waiver for the procedures involving human materials used in this study (Reg. No. 23-0491 KB). Study centers received tryptically digested sample replicates derived from pools of plasma and serum. Each partner was asked to measure five replicates per setup. The sample input amount per injection was restricted to 200 ng for nanoflow setups and to 5 μg for microflow setups. In addition, all partners were asked to measure HeLa standard samples (Pierce HeLa, Thermo Scientific; 50 ng for nanoflow setups; 400 ng for microflow setups) as quality control before and after the round-robin study measurements (see Supporting Figure S1). Also, indexed retention time (iRT) peptides (PROCAL, JPT Peptide Technologies GmbH) were spiked into the samples by each laboratory.^18^ No other restrictions were imposed on the study centers. Samples were measured at three different time points, each 12–16 weeks apart. A complete description of sample preparation and all LC-MS/MS methods can be found in Supporting sections 7–8.

### 2.2 Software Analysis

All MS data was collected and analyzed centrally. The DDA data analysis was performed for every measurement batch separately (5 technical replicates per batch) using MaxQuant (version 2.0.3.0) by searching against the UniProt Human Reference Proteome database (UP000005640_9606, 20950 entries) and an iRT PROCAL sequence database. Default MaxQuant parameters were applied, including label-free quantification and match between runs (MBR) enabled. The LFQ minimum ratio count was set to two peptides. Trypsin was chosen as the enzyme and up to two missed cleavages were allowed. Carbamidomethylation of cysteine was used as a fixed modification, while protein N-terminal acetylation and methionine oxidation were specified as variable modifications. The FDR was set to 1% for both PSMs and protein level. The DIA data was analyzed for every measurement batch separately (5 technical replicates per batch) using MaxDIA (version 2.0.3.0) with a library-based strategy.^19^ The results files from the respective MaxQuant DDA search were used as library. Other parameters were adjusted similarly to the DDA data analysis. For all DIA data an additional processing with DIA-NN (version 1.8.1) in library-free mode was performed including MBR and heuristic protein inference enabled, precursor FDR 1%, and robust LC as quantification strategy.

### 2.3 Statistical Methods

The analysis of output files from MaxQuant and DIA-NN was performed in R (v4.0.4) using the R package mpwR (https://CRAN.R-project.org/package=mpwR). DIA-NN output reports were filtered at a protein group *q*-value under 1% (PG.Qvalue <1%) and a precursor *q*-value under 1% (Q.Value <1%). Downstream analysis was conducted after removing reversed sequences, potential contaminants, only identified by site and PROCAL iRT identifications. Also, considering a drastic drop in IDs or an observable performance decline in the course of the measurements (decline >10%), the following outliers were removed: For plasma – T1_LabG_nLC_qexachfx_R04; T1_LabG_nLC_qexachfx_DIA_R01, R03, R05; T2_LabE_nLC_timspro_DIA_R05; T3_LabE_nLC_timspro_DIA_R04, R05; and for serum – T2_LabG_nLC_qexachfx_DIA_R02; T3_LabB_ultimate_fuslum_DIA_R05; T3_LabE_nLC_timspro_DIA_R05. Details are shown in Figures S2 and S3. The proteins in Figure 7 and Supporting Section “Inter-laboratory reproducibility” are based on the “Majority protein IDs” of MaxQuant́s proteinGroup.txt. Also, only data sets without excluded outliers are considered for these plots resulting in 62 data sets for plasma and 63 data sets for serum. In Figures 8 and 9, the quantitative precision based on LFQ intensities is monitored over time for a subset of proteins, which are detectable both in all plasma and in all serum data sets. For some proteins no LFQ values were calculated, since the LFQ ratio of MaxQuant was set to a minimum of 2 peptides and only 1 peptide was identified. Therefore, also no CV was calculated. This is evident for example for the setup LabA_ultimate_qexachf_DIA in which no CV values are available for protein P02753 in T1 (Figure 9). The same principle applies in Figures S81 and S82. For the calculation of the coefficient of variation (CV) the LFQ intensity columns (not log transformed) of MaxQuant́s proteinGroup.txt were used and it was based on the formula , where σ represents the standard deviation and μ the mean. For Supporting Figure S1, the HeLa measurements for LabE_nLC_timspro were not available for the DIA measurements in T2.

### 2.4 Data Availability

The mass spectrometry proteomics data have been deposited to the ProteomeXchange Consortium via the PRIDE^20^ partner repository with the data set identifier PXD054073.

## 3 Results

**Figure 1:**
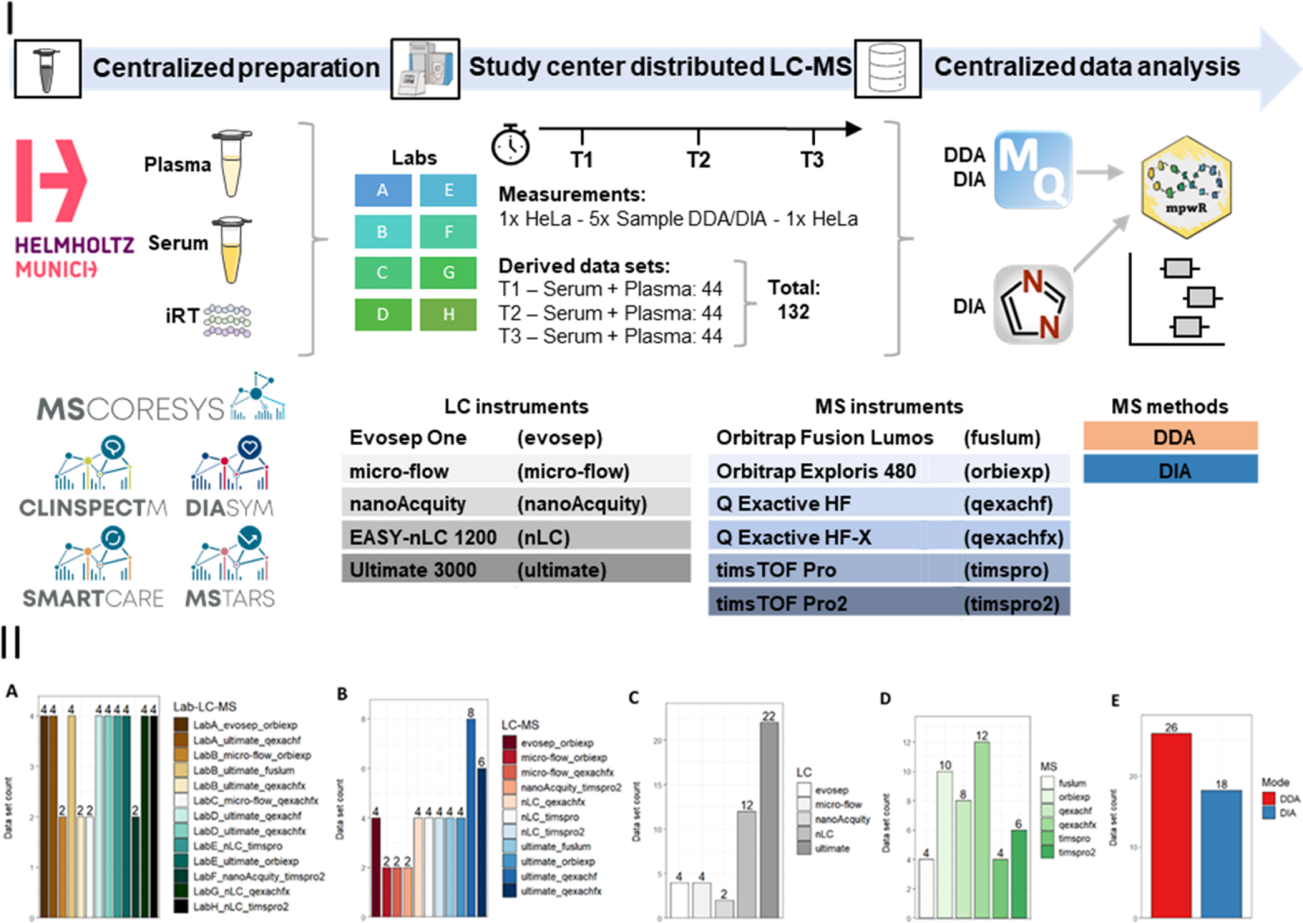
Study design (I) and descriptive summaries of the round-robin study data sets (II) including both plasma and serum analyses for Lab-LC-MS combination (A), LC-MS setups (B), LC systems (C), MS instruments (D), and acquisition mode (E) for one time point, respectively.

The longitudinal round-robin study included 132 data sets generated in eight study centers for both serum and plasma samples and combined across all time points. A depiction of the study design is shown in Figure 1I. In detail, a range of state-of-the-art mass spectrometers were utilized, including models from ThermoFisher (Orbitrap Exploris 480, Q Exactive HF, Q Exactive HF-X, Orbitrap Fusion Lumos) and from Bruker (timsTOF Pro, timsTOF Pro2). The measurements were performed using nanoflow HPLC systems from various manufacturers, including ThermoFisher (Ultimate 3000, EASY-nLC 1200), Evosep (Evosep One), and Waters Corporation (nanoACQUITY). Additionally, microflow LC-MS/MS systems from ThermoFisher were incorporated into the multicenter study. Overall, 11 LC-MS/MS setups were utilized, with the majority of measurements using an Ultimate 3000 connected to a Q Exactive HF setup and an Ultimate 3000 connected to a Q Exactive HF-X system and data were predominantly acquired with DDA methods (60%) (Figure 1II). In addition, a high-level overview of LC-MS parameters is shown in Table 1 and a detailed overview provided in Supporting Table S1. The similarity and divergence between the different setup types and approaches were systematically explored by a variety of PCA plots. In detail, the results were highlighted in three variations: (a) Lab-LC-MS, (b) Laboratory, and (c) MS considering all data sets, all orbitrap MS data sets or all timsTOF MS data sets for plasma and serum, respectively (see Supporting Figures S4–S21). In conclusion every laboratory/setup/instrument type separated, and replicates measured at the same time point clustered together. A clear separation between Orbitrap and timsTOF MS instruments was most evident.

**Table 1:** Overview of Datasets and LC-MS Parameters

laboratory	LC system	MS system	acquisition mode	HPLC column	gradient length [min]	flow rate [nL/min]	MS ranges [m/z]	plasma-median proteins [abs.]	serum-median proteins [abs.]
A	Evosep One	Exploris 480	DDA	Dr. Maisch C18 AQ, (15 cm, 150 μm ID, 1.9 μm beads)	44	variable	375–1500	292	287
			DIA				400–1000	282	268
	Dionex Ultimate 3000 RSLCnano	QExactive HF	DDA	IonOpticks Odyssey column (25 cm × 75 μm, 1.6 μm, C18)	47	300	375–1600	292	288
			DIA				350–1650	280	277
B	Dionex Ultimate 3000 RSLC nano CAP microflow	Exploris 480	DDA	Thermo Fisher Scientific, Acclaim PepMap 100 C18-HPLC column, (150 mm × 1 mm, 2 μm)	30	50,000	360–1300	294	292
	Dionex Ultimate 3000 RSLCnano	Orbitrap Fusion Lumos	DDA	Dr. Maisch ReproSil Gold C18-AQ, 450 mm × 75 μm, 3 μm, self-packed	60	300	360–1300	294	303
			DIA					298	308
	Dionex Ultimate 3000 RSLCnano	QExactive HF-X	DDA	Dr. Maisch ReproSil Gold C18-AQ, 450 mm × 75 μm, 3 μm, self-packed	60	300	360–1300	349	351
C	Thermo Fisher Vanquish	QExactive HF-X	DDA	Thermo Fisher Scientific, Acclaim PepMap 100 C18 column (150 mm, 1 mm ID, 2 μm)	30	50,000	360–1300	325	338
D	Dionex Ultimate 3000 RSLCnano	QExactive HF	DDA	Waters nanoEase M/Z HSS T3 column (25 cm × 75 μm, C18 1.8 μm, 100 Å)	90	250	300–1500	268	263
			DIA				300–1650	316	334
	Dionex Ultimate 3000 RSLCnano	QExactive HF-X	DDA	Waters nanoEase M/Z HSS T3 column (25 cm × 75 μm, C18 1.8 μm, 100 Å)	90	250	300–1500	305	318
			DIA				300–1650	267	257
E	Thermo Fisher Easy nLC 1200	timsTOF Pro	DDA	IonOpticks Aurora Ultimate C18 (1.6 cm, 75 μm, 25 cm)	60	400	100–1700	340	366
			DIA				100–1700	286	294
	Dionex Ultimate 3000 RSLCnano	Exploris 480	DDA	Waters nanoEase MZ BEH C18 (1.7 μm, 130 Å)	50	300	380–1400	342	338
			DIA				350–1400	328	328
F	Waters nanoAcquity LC	timsTOF pro 2	DDA	IonOpticks Aurora Rapid column C18 5 cm × 150 μm, 1.6 μm, analytical emitter column	14	1000	100–1700	188	188
G	Thermo Fisher Easy nLC 1200	QExactive HF-X	DDA	Dr. Maisch ReproSil-Pur C18-AQ particles, 20 cm × 75 μm i.d., 1.9 μm, self-packed	29	250	350–1800	333	339
			DIA				350–1650	297	287
H	Thermo Fisher Easy nLC 1200	timsTOF Pro2	DDA	Dr. Maisch ReproSil-Pur C18-AQ particles, 20 cm × 75 μm i.d., 1.9 μm, self-packed	32	250	100–1700	288	301
			DIA				100–1700	271	285

**Figure 2:**
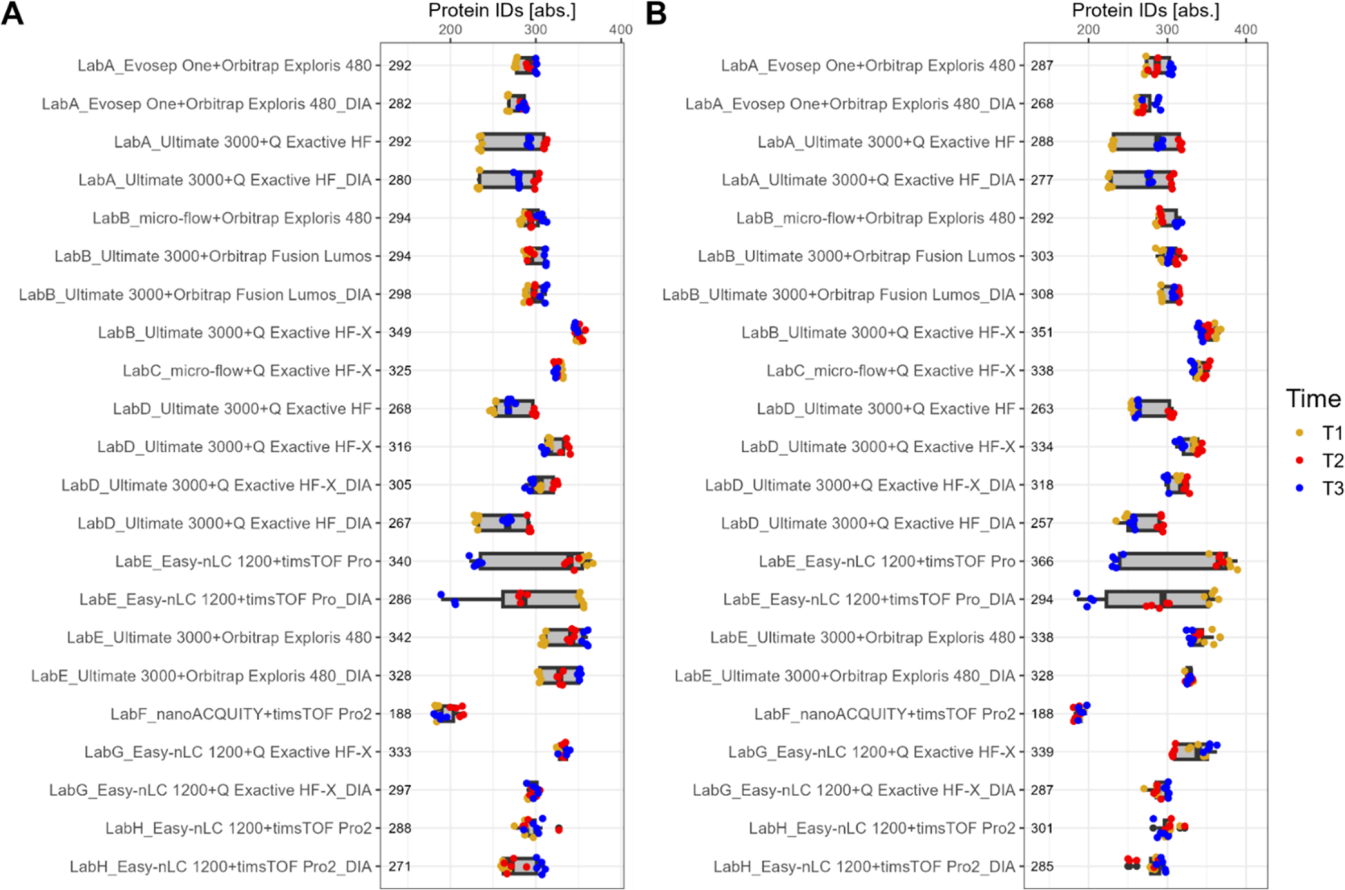
Protein IDs for plasma (A) and serum (B) for different setups. Measurements are color coded by time points. Median number of protein IDs per set up is shown as label.

The performance of the setups was monitored over time in crucial characteristics such as the number of IDs, data completeness, as well as quantitative precision. The boxplots in Figure 2 show the number of IDs on protein-level for plasma (A) and serum (B) including all time points, while dots correspond to individual analyses colored by time point. For both serum and plasma, the setups yielded between 300 and 400 protein IDs, while the majority evolved to around 300 IDs. Also, no tendency for a specific time point is observable, but rather setup specific patterns are visible. For example, if the performance of a setup is best for a specific time point in plasma, in many cases the same trend applied for serum (see e.g., LabA_Ultimate 3000+Q Exactive HF, Figure 2A,B). Furthermore, protein group, peptide and precursor IDs showed similar results (see Supporting Figures S22–S24).

**Figure 3:**
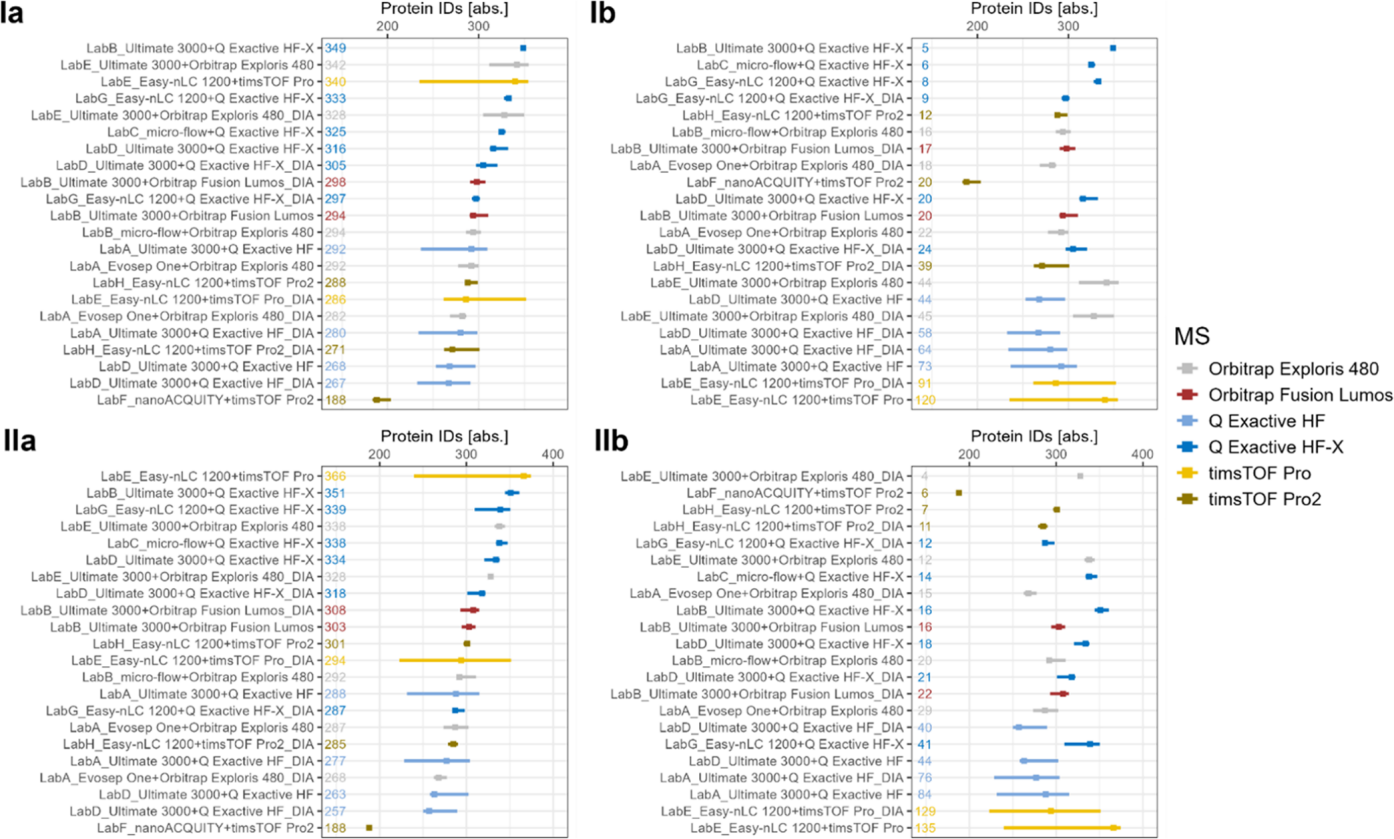
Median number of protein identifications [abs.] and interquartile range (IQR) sorted in decreasing order based on median (a) and in increasing order based on IQR (b) on protein-level for plasma (I) and serum (II). Results are color coded by MS instrument. Labels show median number of protein identifications (a) or IQR (b).

Focusing further on the performance of individual MS instruments and on the variability of the measurements per setup, Figure 3 shows the median and interquartile-range (IQR) on protein-level per setup for both plasma and serum. For both sample types, the same trend is observable for the achieved number of protein IDs, with timsTOF Pro, Orbitrap Exploris 480 and Q Exactive HF-X being among the top performers, and with Q Exactive HF as well as timsTOF Pro2 being among the lowest performing instruments. The variability is highest for timsTOF Pro and Q Exactive HF instruments with an IQR over 50 IDs in case of Q Exactive HF and over 100 IDs in case of timsTOF Pro for both sample types.

**Figure 4:**
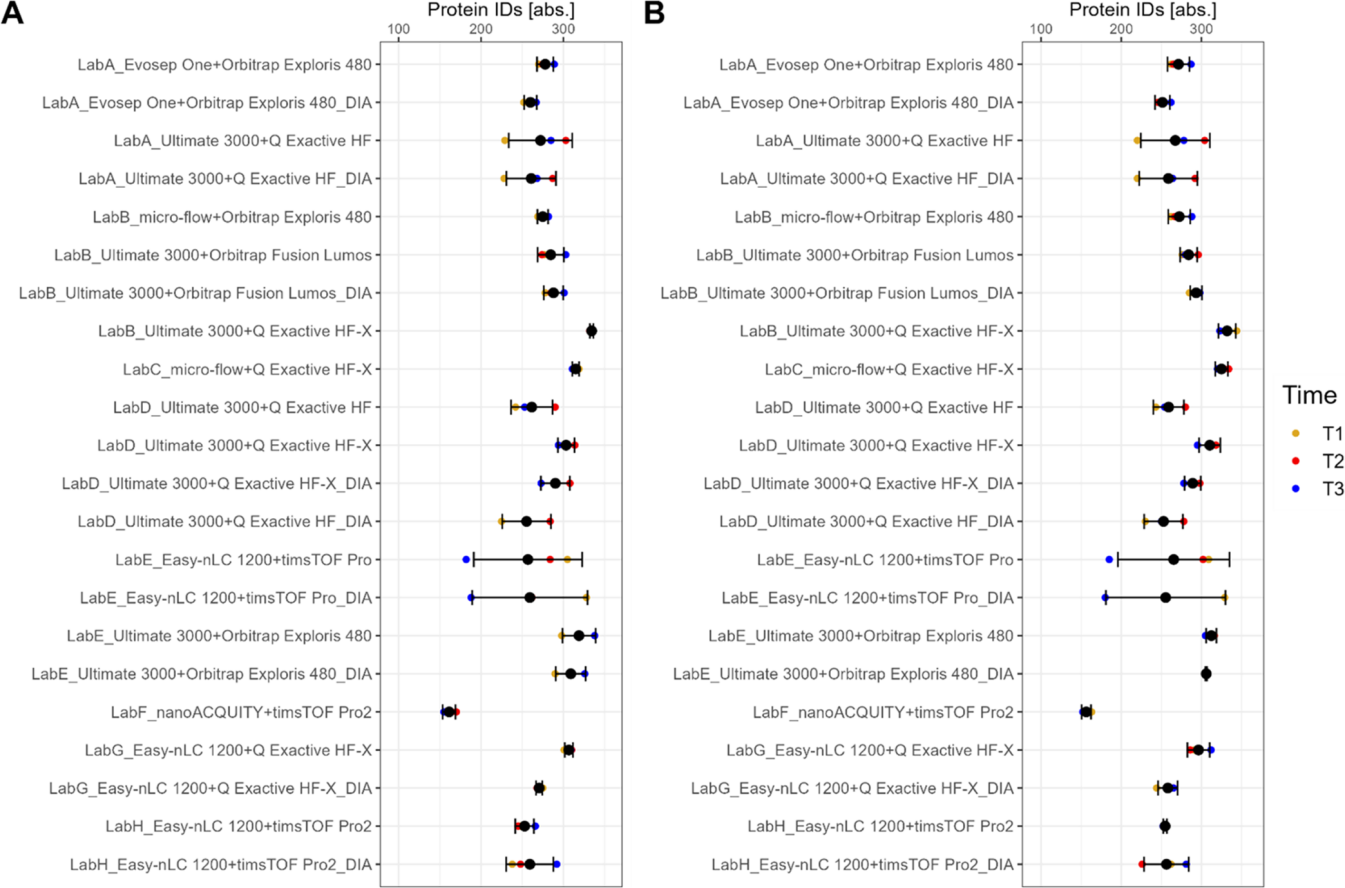
Data completeness based on absolute numbers [abs.] of proteins for plasma (A) and serum (B) for different setups. Only full profiles, which refer to the presence of an identification in each technical replicate run per time point, are displayed and color coded by different time points. Mean and standard deviation as error bars are plotted in black.

Data completeness is presented on protein-level in Figure 4 for plasma and serum. For each time point only full profiles were considered. Full profiles refer to IDs, that were detected in each technical replicate. The mean and standard deviation as error bars are plotted in black. The achieved ID numbers range between 200 IDs and around 340 IDs. This coincides with a relative data completeness of over 90% for most setups (see Supporting Figure S25). Also, similar MS-specific tendencies regarding the intralaboratory reproducibility are noticeable as shown in Figure 3 with the Orbitrap Exploris 480 and Q Exactive HF-X achieving most full profile IDs on average for both plasma and serum (see Supporting Figure S26). In addition, data completeness for protein group-, peptide-, and precursor-level is shown in Supporting Figures S27–S29.

**Figure 5:**
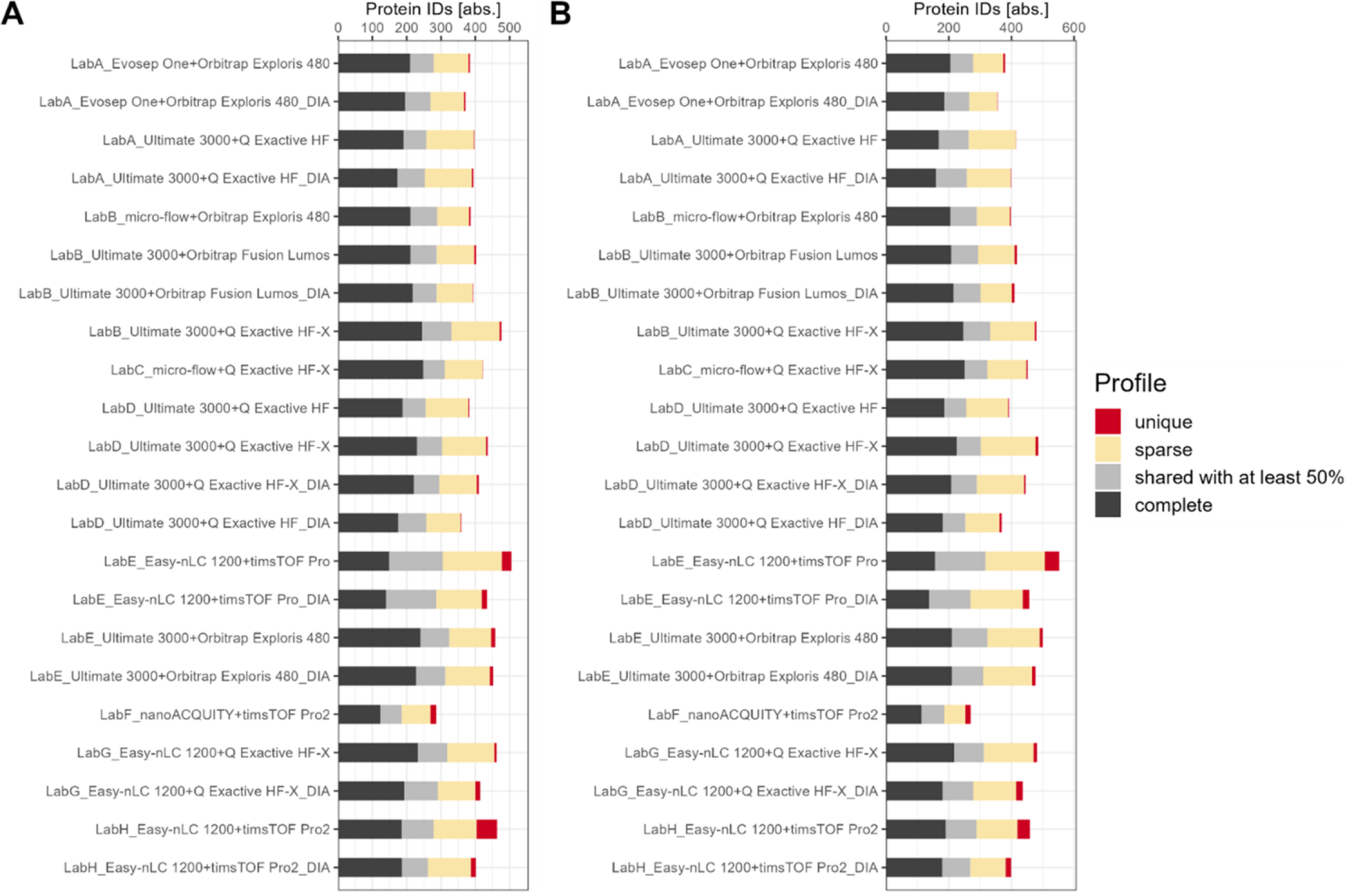
Data completeness based on absolute numbers [abs.] on protein-level for plasma (A) and serum (B). Results of each time point are merged resulting in 15 runs per setup. *Complete* profiles refer to proteins present in all replicate runs (15), *shared with at least 50%* to be at least present in 50% of the replicate runs, *sparse* to be present in more than one run and less than 50% of the runs, and *unique* to be only present in one replicate run.

The overall data completeness for proteins, which are present in each technical replicate per setup and in *all* time points combined, is shown in Figure 5. For both sample types, these detected IDs range between 150 IDs to 200 IDs per setup. Accordingly, the overall relative data completeness is reduced from over 90% per time point to around 25–50% data completeness for all time points combined (see Supporting Figure S30).

**Figure 6:**
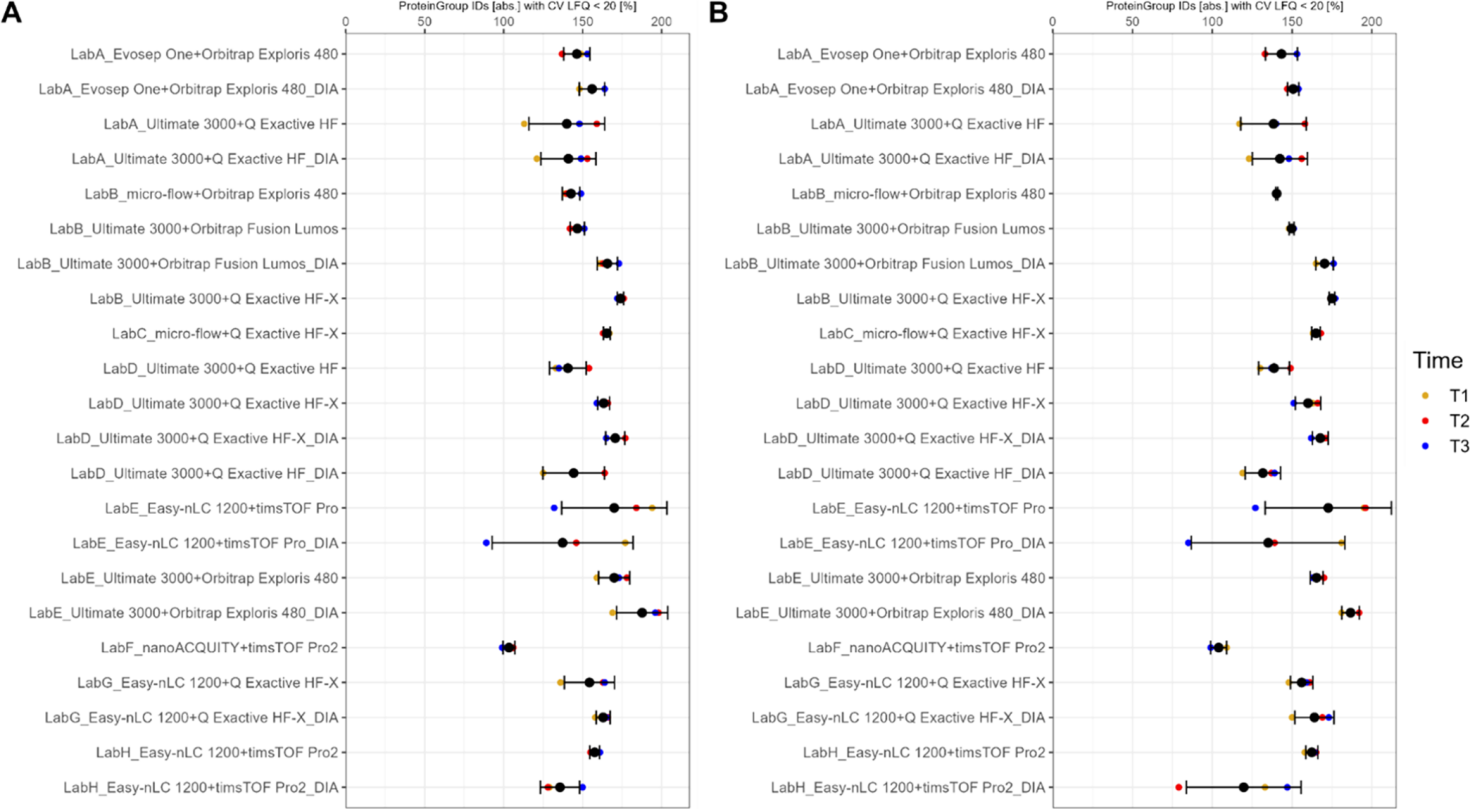
Quantitative precision coefficient of variation (CV) LFQ < 20% on absolute numbers [abs.] on protein group-level for plasma (A) and serum (B). The different time points are color coded.

Monitoring quantitative precision over time was a key aspect to evaluate the performance of the different setups. In general, the median quantitative precision of label-free quantification (LFQ) ranges between 2–12% for plasma and between 2–15% for serum for the respective setups (see Supporting Figures S31 and S32). In addition, by binning the intensity range as well as the peptide per protein group distribution into quartiles, respectively, clear tendencies are observed. The highest precision is achieved in the highest intensity range as well as in the range with protein groups based on the highest number of peptides (see Supporting Figures S33–S58). For a closer focus, the number of protein group IDs with a CV under 20% was analyzed for the different time points and setups, respectively (Figure 6). Most setups achieve around 150 IDs with a CV lower than 20% irrespective of the sample type. In detail, Orbitrap Exploris 480 and Q Exactive HF-X MS instruments are among the top performers and the highest variability is demonstrated by timsTOF Pro and Q Exactive HF MS instruments (see Supporting Figure S59).

**Figure 7:**
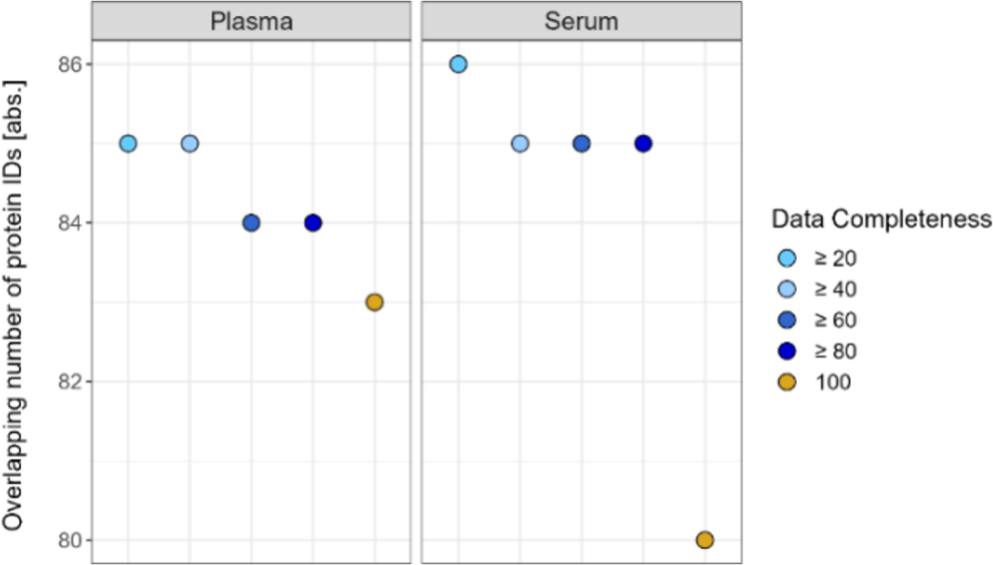
Interlaboratory reproducibility - overlapping protein IDs [abs.] for plasma and serum with different levels of data completeness [%] based on all available data sets per sample type, respectively. For plasma 62 data sets and for serum 63 data sets are considered.

As qualitative indicator for interlaboratory reproducibility the data completeness was assessed by compiling all available data sets across setups and including every time point per sample type, respectively. The overall overlap results in 83 and 80 protein IDs for plasma and serum, respectively (Figure 7). Details are provided in Supporting Figure S60. Interestingly, reducing data completeness per data set has no major effect on the detected overlapping protein IDs. For example, in the case of ≥20% data completeness, the number of protein IDs increases for plasma by 2 and for serum by 6 protein IDs. Moreover, the total number of detected protein IDs considering 100% data completeness per data set but combining all data sets results in 612 IDs for plasma and 653 IDs for serum (see Supporting Figure S61). Similar tendencies are observable at peptide-level (see Supporting Figures S62 and S63). Of the proteins, which are identified in each technical replicate across all data sets, 71 IDs overlap between plasma and serum (see Supporting Figure S64). In addition, highlighting intensity profiles per data set shows that overlapping protein identifications are predominantly detected in a higher intensity range in each setup and for both plasma and serum (see Supporting Figures S65–S76). However, focusing on one-to-one comparisons between setups, the overlap [%] for both sample types and both on protein- and peptide-level is mostly above 60% (see Supporting Figures S77–S80).

**Figure 8:**
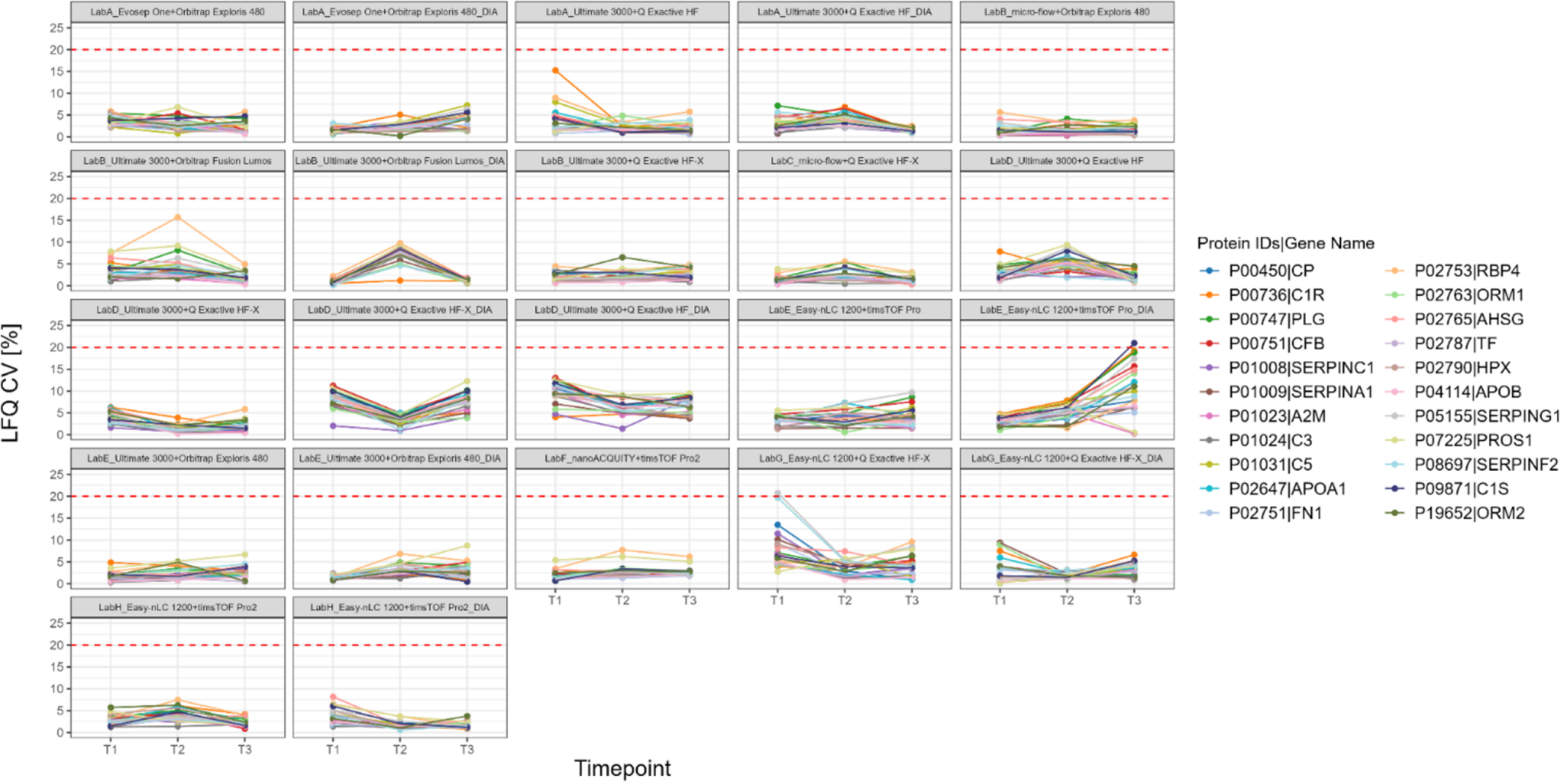
Quantitative precision across time points and per setup for FDA approved biomarker proteins^21^ in plasma. Red dashed horizontal line indicates CV 20%.

**Figure 9:**
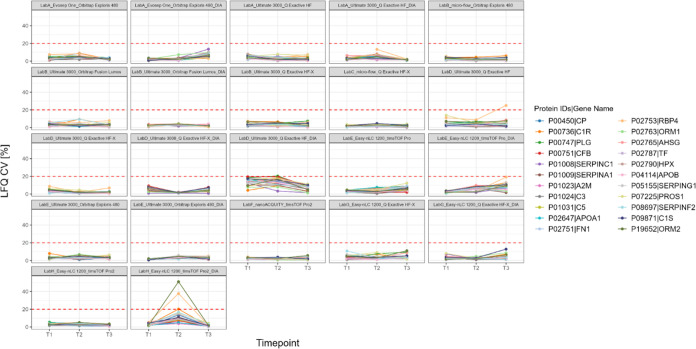
Quantitative precision across time points and per setup for FDA approved biomarker proteins^21^ in serum. Red dashed horizontal line indicates CV 20%.

These overlapping 71 protein IDs were compared to FDA-approved biomarkers, which were listed by Anderson et al.^21^ In total, 22 of the 71 protein IDs are reported to be FDA-approved biomarkers. A detailed list is provided in Supporting Table S2 and an overview of the CVs for all these proteins per set up is displayed in Supporting Figures S81 and S82 for plasma and serum, respectively. Highlighting the clinical utility of the LC-MS/MS workflows, the quantitative precision based on LFQ intensities of the determined 22 biomarkers is displayed for each time point for plasma in Figure 8 and for serum in Figure 9. For both sample types most proteins across all setups have a quantitative CV of less than 20%, which resembles a cutoff commonly used for in vitro diagnostic assays. Occasionally, individual proteins show a higher CV than 20% in specific data sets, for example in T2 for LabH_Easy-nLC 1200+timsTOF Pro2_DIA retinol-binding protein 4 (P02753) with a CV of 38% and α-1-acid glycoprotein 2 (P19652) with a CV of 51% (Figure 9). Also, this is accompanied by a general shift toward higher CV values of all proteins for the respective time point and setup.

## 4 Discussion

The major objective of the round-robin study was to evaluate the performance of measuring plasma and serum samples over time with a particular emphasis on the variability observed across time points for essential performance characteristics such as number of IDs, data completeness, and quantitative precision. Additionally, the study focused on interlaboratory reproducibility by highlighting proteins consistently detected on each platform across all sites to determine a baseline for detectable plasma and serum proteins without utilizing any enrichment, depletion, or fractionation workflow.

In contrast to our previous study^22^ digested plasma and serum samples ready for MS injection were distributed between the participating laboratories, so variances originating from sample preparation were fully avoided. Further, the peptide injection amount was standardized between all measurements and laboratories. However, again, no further guidelines, protocols, or restrictions were imposed on the laboratories with respect to LC-MS/MS measurement settings. In detail, the injection amount was restricted to 200 ng for nanoflow setups and to 5 μg for microflow systems for both sample types. Arguably, for nanoflow setups coupled to Orbitrap instruments, 200 ng might be a potentially insufficient amount, which could result in lower ID numbers. On the other hand, for nanoflow setups coupled to timsTOF Pro devices 200 ng input amount is at the upper limit of suitability. Furthermore, by separately analyzing all technical replicates for a specific setup and time point per sample type the ID rate, data completeness as well as quantitative precision across time points might be impaired. Analyzing for example all time points (e.g., plasma: T1, T2, T3) for a specific setup and sample in a combined software analysis might improve the outcome. For instance, the effect of the enabled settings MBR and LFQ in MaxQuant could lead to higher ID rates, improved overall data completeness, and enhanced quantitative precision when all data sets for a setup and sample type are used, irrespective of the time points. However, the individual analysis per time point presents a closer picture of daily clinical routine, in which an instant analysis of MS results is imperative and large-scale analyses are not feasible and desirable. In addition, it is noteworthy that the library-based approach applied in this study potentially limits the performance of the DIA-based measurements, since the DDA counterpart was used as library input. Utilizing a library with greater depth or choosing a library-free method could potentially enhance identification rate and other factors like data completeness, and quantitative precision. As an example, all DIA data sets were also analyzed with DIA-NN in library-free mode, and the results showed higher ID rates for every DIA data set (see supplementary Figure S83). However, the study was not designed to explore protein depth, but rather focused on monitoring essential performance characteristics over time for the clinically relevant blood-derived sample types and thus on proteins that can be consistently detected and demonstrate a clinical value.

In both serum and plasma samples, the setups identify between 300 and 400 protein IDs, with timsTOF Pro, Orbitrap Exploris 480, and Q Exactive HF-X emerging as top performers, while Q Exactive HF is among the lower performers. In addition, ID rate variability across time points is highest for timsTOF Pro and Q Exactive HF instruments. However, no direct correlation can be made between the highest ID rate and highest variability. Most setups achieve a data completeness of over 90% on protein-level consistently across time points. The quantitative precision of LFQ intensities shows that most setups can achieve around 150 IDs with a CV lower than 20%, regardless of the sample type. In detail, Orbitrap Exploris 480, and Q Exactive HF-X MS instruments are among the most precise performers, while timsTOF Pro and Q Exactive HF instruments display the highest variability.

Another essential aspect of the study was to assess interlaboratory reproducibility. For plasma 83 protein IDs and for serum 80 protein IDs are detected in each technical replicate and across every setup. In fact, 71 of these protein IDs are present in both plasma and serum. Note, that lowering data completeness requirements for individual data sets has only a minor influence on the absolute number of overlapping IDs. This suggests that increasing data completeness to achieve better intralaboratory repeatability does not inevitably result in improved interlaboratory reproducibility. Considering the total number of detected protein IDs at 100% data completeness per setup for plasma (612 IDs) and for serum (653 IDs), emphasizes the presence of setup specific protein signatures. As discussed, interlaboratory reproducibility is expected to be higher if other bioinformatic strategies are applied. In addition, considering all data sets has the disadvantage that low-performing setups have a high influence on the overall achieved overlap. To address this, a detailed pairwise analysis was conducted, focusing exclusively on two setups at a time. This analysis demonstrated an overlap greater than 60% in most cases, highlighting the interlaboratory reproducibility across setups and laboratories.

To showcase the potential of the robustly detected 71 protein IDs, which are measurable both in plasma and serum, these proteins were matched against an FDA-approved biomarker list.^21^ A total of 22 protein IDs were among these known biomarkers and an additional evaluation of the quantitative precision over time for these proteins per setup revealed that the applied MS measurements demonstrate excellent reproducibility based on FDA criteria (CV < 20%).

As a conclusion, when measuring clinically relevant specimens such as plasma and serum great intralaboratory reproducibility was achieved for essential performance characteristics. Moreover, despite a conservative bioinformatic analysis workflow, 71 protein IDs are reproducibly detectable in each study center and 22 of these protein IDs are already known FDA-approved biomarkers, which also display excellent quantitative precision in each LC-MS/MS setup. We acknowledge the fact that streamlined workflows already have been developed to achieve a broader protein depth including covering more FDA-approved biomarkers and with high sample throughput.^9,10,23^ Nevertheless, the results of this nation-wide longitudinal study emphasize that the applied LC-MS/MS measurements and the bioinformatic analysis in its simplest form create an intriguing basis for the development of clinical applications.

## Supporting Information Available

Table S1. Overview of LC-MS parameters per setup. Table S2. Overlapping plasma and serum proteins which are present both in all plasma and serum data sets; also indicated FDA approved biomarker proteins according to Anderson et al. Table S3. Measurement scheme of setup Easy nLC 1200 + QExactive HF-X from laboratory F. Figure S1. QC HeLa protein group IDs before/after plasma and serum samples. Figure S2. Outlier detection–plasma samples. Figure S3. Outlier detection–serum samples. Figure S4. Plasma – PCA: all data sets; color coded by Lab-LC-MS/MS. Figure S5. Plasma – PCA: all data sets; color coded by Lab. Figure S6. Plasma – PCA: all data sets; color coded by MS. Figure S7. Plasma – PCA: timsTOF data sets; color coded by Lab-LC-MS/MS. Figure S8. Plasma – PCA: timsTOF data sets; color coded by Lab. Figure S9. Plasma – PCA: timsTOF data sets; color coded by MS. Figure S10. Plasma – PCA: orbitrap data sets; color coded by Lab-LC-MS/MS. Figure S11. Plasma – PCA: orbitrap data sets; color coded by Lab. Figure S12. Plasma – PCA: orbitrap data sets; color coded by MS. Figure S13. Serum – PCA: all data sets; color coded by Lab-LC-MS/MS. Figure S14. Serum – PCA: all data sets; color coded by Lab. Figure S15. Serum – PCA: all data sets; color coded by MS. Figure S16. Serum – PCA: timsTOF data sets; color coded by Lab-LC-MS/MS. Figure S17. Serum – PCA: timsTOF data sets; color coded by Lab. Figure S18. Serum – PCA: timsTOF data sets; color coded by MS. Figure S19. Serum – PCA: orbitrap data sets; color coded by Lab-LC-MS/MS. Figure S20. Serum – PCA: orbitrap data sets; color coded by Lab. Figure S21. Serum – PCA: orbitrap data sets; color coded by MS. Figure S22. Protein Group IDs for plasma and serum samples. Figure S23. Peptide IDs for plasma and serum samples. Figure S24. Precursor IDs for plasma and serum samples. Figure S25. Relative data completeness on protein-level for plasma and serum samples per time point. Figure S26. Absolute data completeness on protein-level for plasma and serum samples with mean and standard deviation. Figure S27. Absolute data completeness on protein group-level for plasma and serum samples. Figure S28. Absolute data completeness on peptide-level for plasma and serum samples. Figure S29. Absolute data completeness on precursor-level for plasma and serum samples. Figure S30. Relative data completeness on protein-level for plasma and serum samples with combined time points. Figure S31: Plasma – CV distribution of all data sets. Figure S32: Serum – CV distribution of all data sets. Figure S33: Plasma – CV distribution vs binned intensity distribution for Orbitrap Exploris 480 data sets. Figure S34: Plasma - CV distribution vs binned intensity distribution for Orbitrap Fusion Lumos data sets. Figure S35: Plasma - CV distribution vs binned intensity distribution for Q Exactive HF data sets. Figure S36: Plasma – CV distribution vs binned intensity distribution for Q Exactive HF-X data sets. Figure S37: Plasma – CV distribution vs binned intensity distribution for timsTOF Pro data sets. Figure S38: Plasma – CV distribution vs binned intensity distribution for timsTOF Pro2 data sets. Figure S39: Serum - CV distribution vs binned intensity distribution for Orbitrap Exploris 480 data sets. Figure S40: Serum – CV distribution vs binned intensity distribution for Orbitrap Fusion Lumos data sets. Figure S41: Serum – CV distribution vs binned intensity distribution for Q Exactive HF data sets. Figure S42: Serum – CV distribution vs binned intensity distribution for Q Exactive HF-X data sets. Figure S43: Serum – CV distribution vs binned intensity distribution for timsTOF Pro data sets. Figure S44: Serum - CV distribution vs binned intensity distribution for timsTOF Pro2 data sets. Figure S45: Plasma – Distribution of peptides per protein group of all data sets. Figure S46: Serum – Distribution of peptides per protein group of all data sets. Figure S47: Plasma – CV distribution vs binned peptides per protein group distribution for Orbitrap Exploris 480 data sets. Figure S48: Plasma – CV distribution vs binned peptides per protein group distribution for Orbitrap Fusion Lumos data sets. Figure S49: Plasma – CV distribution vs binned peptides per protein group distribution for Q Exactive HF data sets. Figure S50: Plasma – CV distribution vs binned peptides per protein group distribution for Q Exactive HF-X data sets. Figure S51: Plasma – CV distribution vs binned peptides per protein group distribution for timsTOF Pro data sets. Figure S52: Plasma – CV distribution vs binned peptides per protein group distribution for timsTOF Pro2 data sets. Figure S53: Serum – CV distribution vs binned peptides per protein group distribution for Orbitrap Exploris 480 data sets. Figure S54: Serum – CV distribution vs binned peptides per protein group distribution for Orbitrap Fusion Lumos data sets. Figure S55: Serum – CV distribution vs binned peptides per protein group distribution for Q Exactive HF data sets. Figure S56: Serum – CV distribution vs binned peptides per protein group distribution for Q Exactive HF-X data sets. Figure S57: Serum – CV distribution vs binned peptides per protein group distribution for timsTOF Pro data sets. Figure S58: Serum – CV distribution vs binned peptides per protein group distribution for timsTOF Pro2 data sets. Figure S59. Protein group IDs [abs.] with CV LFQ < 20% for plasma and serum samples. Figure S60. Absolute overlap of protein IDs for plasma and serum samples across all data sets. Figure S61. Total number of protein IDs and relative overlap of IDs for plasma and serum samples across all data sets. Figure S62. Absolute overlap of peptide IDs for plasma and serum samples across all data sets. Figure S63. Total number of peptide IDs and relative overlap of IDs for plasma and serum samples across all data sets. Figure S64. Upset plot for comparing overlapping protein IDs for plasma and serum samples. Figure S65. Plasma sample intensity distributions for data sets measured on Orbitrap Exploris 480. Figure S66. Plasma sample intensity distributions for data sets measured on Orbitrap Fusion Lumos instruments. Figure S67. Plasma sample intensity distributions for data sets measured on Q Exactive HF instruments. Figure S68. Plasma sample intensity distributions for data sets measured on Q Exactive HF-X instruments. Figure S69. Plasma sample intensity distributions for data sets measured on timsTOF Pro instruments. Figure S70. Plasma sample intensity distributions for data sets measured on timsTOF Pro2 instruments. Figure S71. Serum sample intensity distributions for data sets measured on Orbitrap Exploris 480. Figure S72. Serum sample intensity distributions for data sets measured on Orbitrap Fusion Lumos instruments. Figure S73. Serum sample intensity distributions for data sets measured on Q Exactive HF instruments. Figure S74. Serum sample intensity distributions for data sets measured on Q Exactive HF-X instruments. Figure S75. Serum sample intensity distributions for data sets measured on timsTOF Pro instruments. Figure S76. Serum sample intensity distributions for data sets measured on timsTOF Pro2 instruments. Figure S77. Plasma – Pairwise comparison heatmap displaying overlap [%] on protein-level. Figure S78. Plasma – Pairwise comparison heatmap displaying overlap [%] on peptide-level. Figure S79. Serum – Pairwise comparison heatmap displaying overlap [%] on protein-level. Figure S80. Serum – Pairwise comparison heatmap displaying overlap [%] on peptide-level. Figure S81. Plasma – Heatmap displaying the LFQ CV [%] for commonly quantified protein groups across all setups. Figure S82. Serum – Heatmap displaying the LFQ CV [%] for commonly quantified protein groups across all setups. Figure S83. Protein IDs for plasma and serum samples for all DIA data sets achieved by DIA-NN processing (PDF)
Table S1: Overview about LC-MS/MS parameter and identified proteins (XLSX)
Table S2: Overlapping plasma and serum proteins which are present both in all plasma and serum datasets (XLSX)

## Abbreviations

MS: mass spectrometry
LC: liquid chromatography
DDA: data-dependent acquisition
DIA: data-independent acquisition
IDs: identifications
DC: data completeness
MBR: match between runs
LFQ: label-free quantification
CV: coefficient of variation
FDA: Food and Drug Administration
evosep: Evosep One
ultimate: Ultimate 3000
nLC: Easy-nLC 1200
qexachf: Q Exactive HF
qexachfx: Q Exactive HF-X
fuslum: Fusion Lumos
orbiexp: Orbitrap Exploris
timspro: timsTOF Pro
timspro2: timsTOF Pro2
T1: first measurement time point of this study
T2: second measurement time point of this study
T3: third measurement time point of this study
