# Correction to “Multicenter Longitudinal Quality Assessment of MS-Based Proteomics in Plasma and Serum”

**DOI:** 10.1021/acs.jproteome.5c00609

**Published:** 2025-07-25

**Authors:** Oliver Kardell, Thomas Gronauer, Christine von Toerne, Juliane Merl-Pham, Ann-Christine König, Teresa K. Barth, Julia Mergner, Christina Ludwig, Johanna Tüshaus, Pieter Giesbertz, Stephan Breimann, Lisa Schweizer, Torsten Müller, Georg Kliewer, Ute Distler, Karim Aljakouch, David Gomez-Zepeda, Oliver Popp, Di Qin, Daniel Teupser, Jürgen Cox, Axel Imhof, Bernhard Küster, Stefan F. Lichtenthaler, Jeroen Krijgsveld, Stefan Tenzer, Philipp Mertins, Fabian Coscia, Stefanie M. Hauck

**Affiliations:** † Metabolomics and Proteomics Core, Helmholtz Zentrum München, German Research Center for Environmental Health, 80939 Munich, Germany; ‡ Clinical Protein Analysis Unit (ClinZfP), Biomedical Center, Faculty of Medicine, LMU Munich, 82152 Martinsried, Germany; § Bavarian Center for Biomolecular Mass Spectrometry at Klinikum rechts der Isar (BayBioMS@MRI), Technical University of Munich, 80333 Munich, Germany; ∥ Bavarian Center for Biomolecular Mass Spectrometry (BayBioMS), School of Life Sciences, Technical University of Munich, 85354 Freising, Germany; ⊥ German Center for Neurodegenerative Diseases (DZNE) Munich, DZNE, 81377 Munich, Germany; # Department of Proteomics and Signal Transduction, Max-Planck Institute of Biochemistry, 82152 Martinsried, Germany; 7 German Cancer Research Center (DKFZ), 69120 Heidelberg, Germany; 8 Medical Faculty, Heidelberg University, 69120 Heidelberg, Germany; 9 Institute for Immunology, University Medical Center of the Johannes Gutenberg University Mainz, 55131 Mainz, Germany; 10 Immunoproteomics Unit, Helmholtz-Institute for Translational Oncology (HI-TRON) Mainz, 55131 Mainz, Germany; 11 Max-Delbrück-Center for Molecular Medicine in the Helmholtz Association (MDC), 13125 Berlin, Germany; 12 Institute of Laboratory Medicine, University Hospital, LMU Munich, 81377 Munich, Germany; 13 Computational Systems Biochemistry Research Group, Max-Planck Institute of Biochemistry, Martinsried 82152, Germany; 14 Proteomics and Bioanalytics, School of Life Sciences, Technical University of Munich, 85354 Freising, Germany; 15 Neuroproteomics, School of Medicine and Health, Klinikum rechts der Isar, Technical University of Munich, 81675 Munich, Germany; 16 Munich Cluster for Systems Neurology (SyNergy), 81377 Munich, German

Karim Aljakouch contributed measurements and data to the manuscript, in line with the contributions of the other coauthors involved in this multicenter study, and therefore fully merits coauthorship. Unfortunately, he was inadvertently omitted from the original submission—an oversight for which I sincerely apologize. This change is reflected in the authorship of this Correction.

