# Supplementary material for: Multicenter Longitudinal Quality Assessment of MS-Based Proteomics in Plasma and Serum
Source: J Proteome Res. 2025 Feb 7;24(3):1017–29. doi: 10.1021/acs.jproteome.4c00644 (PMC11894660; doi:10.1021/acs.jproteome.4c00644)
Supplement: Supplementary file 1 — pr4c00644_si_001.pdf [file pr4c00644_si_001.pdf]

Multicenter longitudinal quality assessment of MS-based proteomics in plasma and serum

Oliver Kardell[1], Thomas Gronauer[1], Christine von Toerne[1], Juliane Merl-Pham[1], Ann-Christine König[1], Teresa K. Barth[2], Julia Mergner[3], Christina Ludwig[4], Johanna Tüshaus[4], Pieter Giesbertz[5,14], Stephan Breimann[5], Lisa Schweizer[6], Torsten Müller[7,8], Georg Kliewer[7,8], Ute Distler[9], David Gomez-Zepeda[7,9,10], Oliver Popp[11], Di Qin[11], Daniel Teupser[12], Jürgen Cox[13], Axel Imhof[2], Bernhard Küster[4,14], Stefan F. Lichtenthaler[5,15,16], Jeroen Krijgsveld[7,8], Stefan Tenzer[9,10], Philipp Mertins[11], Fabian Coscia[11], Stefanie M. Hauck[1*]

[1]Metabolomics and Proteomics Core, Helmholtz Zentrum München, German Research Center for Environmental Health, 80939 Munich, Germany;

[2]Clinical Protein Analysis Unit (ClinZfP), Biomedical Center, Faculty of Medicine, LMU Munich, 82152 Martinsried, Germany;

[3]Bavarian Center for Biomolecular Mass Spectrometry at Klinikum rechts der Isar (BayBioMS@MRI), Technical University of Munich, 80333 Munich, Germany;

[4]Bavarian Center for Biomolecular Mass Spectrometry (BayBioMS), School of Life Sciences, Technical University of Munich, 85354 Freising, Germany;

[5]German Center for Neurodegenerative Diseases (DZNE) Munich, DZNE, 81377 Munich, Germany;

[6]Department of Proteomics and Signal Transduction, Max-Planck Institute of Biochemistry, 82152 Martinsried, Germany;

[7]German Cancer Research Center (DKFZ), 69120 Heidelberg, Germany;

[8]Medical Faculty, Heidelberg University, 69120 Heidelberg, Germany;

[9]Institute for Immunology, University Medical Center of the Johannes Gutenberg University Mainz, 55131 Mainz, Germany;

[10]Immunoproteomics Unit, Helmholtz-Institute for Translational Oncology (HI-TRON) Mainz, 55131 Mainz, Germany;

[11]Max-Delbrück-Center for Molecular Medicine in the Helmholtz Association (MDC), 13125 Berlin, Germany;

[12]Institute of Laboratory Medicine, University Hospital, LMU Munich, 81377 Munich, Germany;

[13]Computational Systems Biochemistry Research Group, Max-Planck Institute of Biochemistry, Martinsried 82152, Germany.

[14]Proteomics and Bioanalytics, School of Life Sciences, Technical University of Munich, 85354 Freising, Germany

[15]Neuroproteomics, School of Medicine and Health, Klinikum rechts der Isar, Technical University of Munich, 81675 Munich, Germany

[16]Munich Cluster for Systems Neurology (SyNergy), 81377 Munich, Germany

*For correspondence: Stefanie M. Hauck, stefanie.hauck@helmholtz-munich.de

Contents

1. HeLa Measurements 5

2. Outlier detection 6

3. Principle component analysis 7

3.1 Plasma – all datasets 7

3.2 Plasma - timsTOF 9

3.3 Plasma - Orbitrap 11

3.4 Serum – all datasets 13

3.5 Serum – timsTOF 15

3.6 Serum - Orbitrap 17

4. Identifications 19

4.1 Protein group-level 19

4.2 Peptide-level 20

4.3 Precursor-level 21

5. Data Completeness 22

5.1 Protein-level: relative DC – T1 vs. T2 vs. T3 22

5.2 Protein-level: Mean and Standard Deviation 23

5.3 Protein group-level: absolute DC – T1 vs. T2 vs. T3 24

5.4 Peptide-level: absolute DC – T1 vs. T2 vs. T3 25

5.5 Precursor-level: absolute DC – T1 vs. T2 vs. T3 26

5.6 Protein-level: relative DC – T1 + T2 + T3 27

6. Quantitative Precision 28

6.1 CV distribution 28

6.2 Plasma: CV vs. Intensity distribution binned 30

6.3 Serum: CV vs. Intensity distribution binned 33

6.4 Peptides per protein group distribution 36

6.5 Plasma: CV vs. Binned peptides per protein group distribution 38

6.6 Serum: CV vs. Binned peptides per protein group distribution 42

6.7 Protein group-level: Mean and Standard Deviation 46

7. Inter-laboratory reproducibility 47

7.1 Protein-level: Absolute overlap of IDs 47

7.2 Protein-level: Total number of IDs & relative overlap of IDs 48

7.3 Peptide-level: Absolute overlap of IDs 49

7.4 Peptide-level: Total number of IDs & relative overlap of IDs 50

7.5 Upset plot ... 51
7.6 Protein-level: Intensity distributions ... 52
8. Overlap [%] – pairwise comparisons ... 62
9. CV heatmap of common protein groups including all set ups ... 66
10. DIA-NN Identifications ... 68
10.1 Protein-level ... 68
11. Sample preparation ... 69
12. LC-MS/MS settings ... 69
12.1 Laboratory A ... 69
12.2 Laboratory B ... 71
12.3 Laboratory C ... 74
12.4 Laboratory D ... 75
12.5 Laboratory E ... 77
12.6 Laboratory F ... 79
12.7 Laboratory G ... 80
12.8 Laboratory H ... 83

1. HeLa Measurements

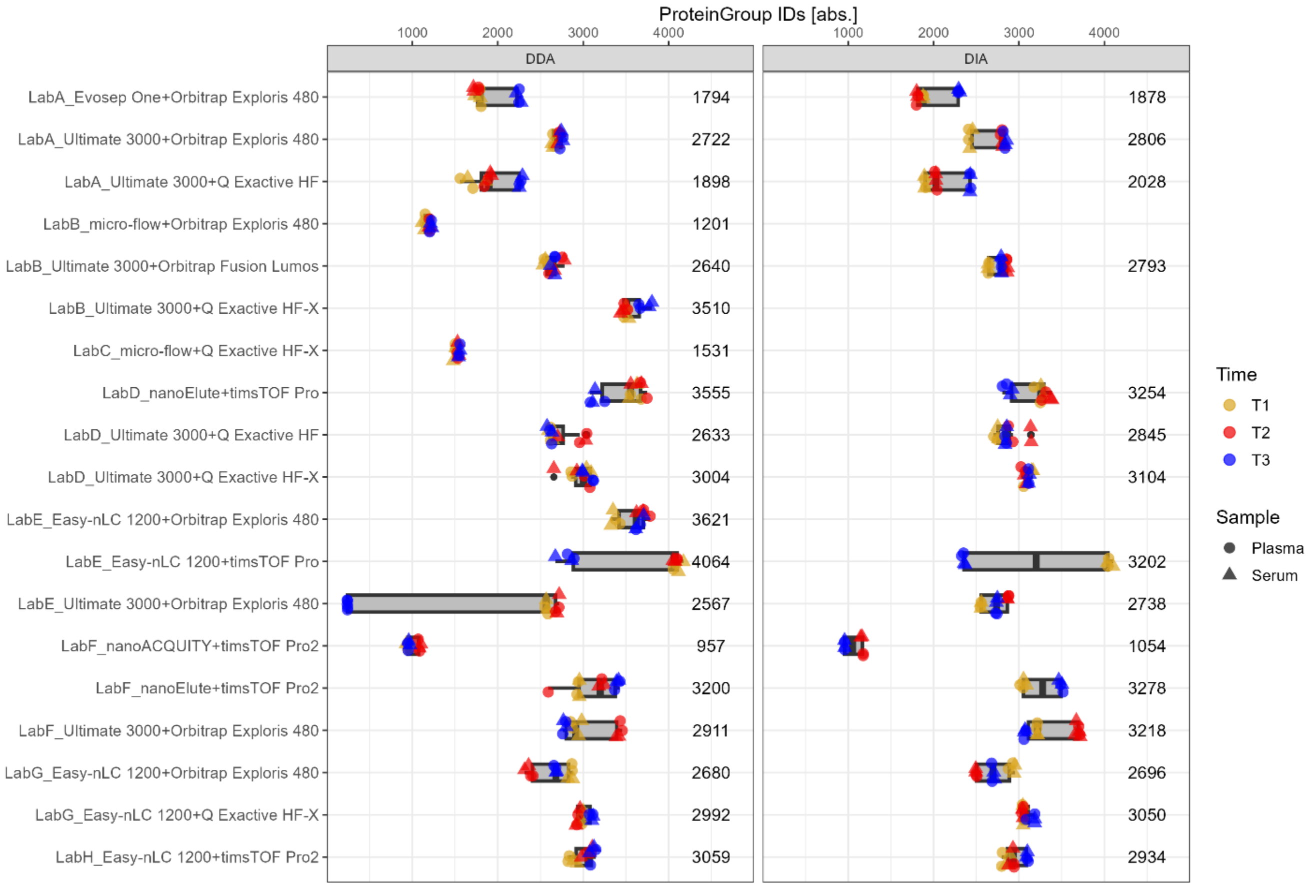

Fig. S1: HeLa protein group IDs per analysis set up before/after plasma and serum sample measurements at different time points, respectively. Time point is color coded and sample type is shown in different shapes. Median protein group IDs per set up is shown as label.

2. Outlier detection

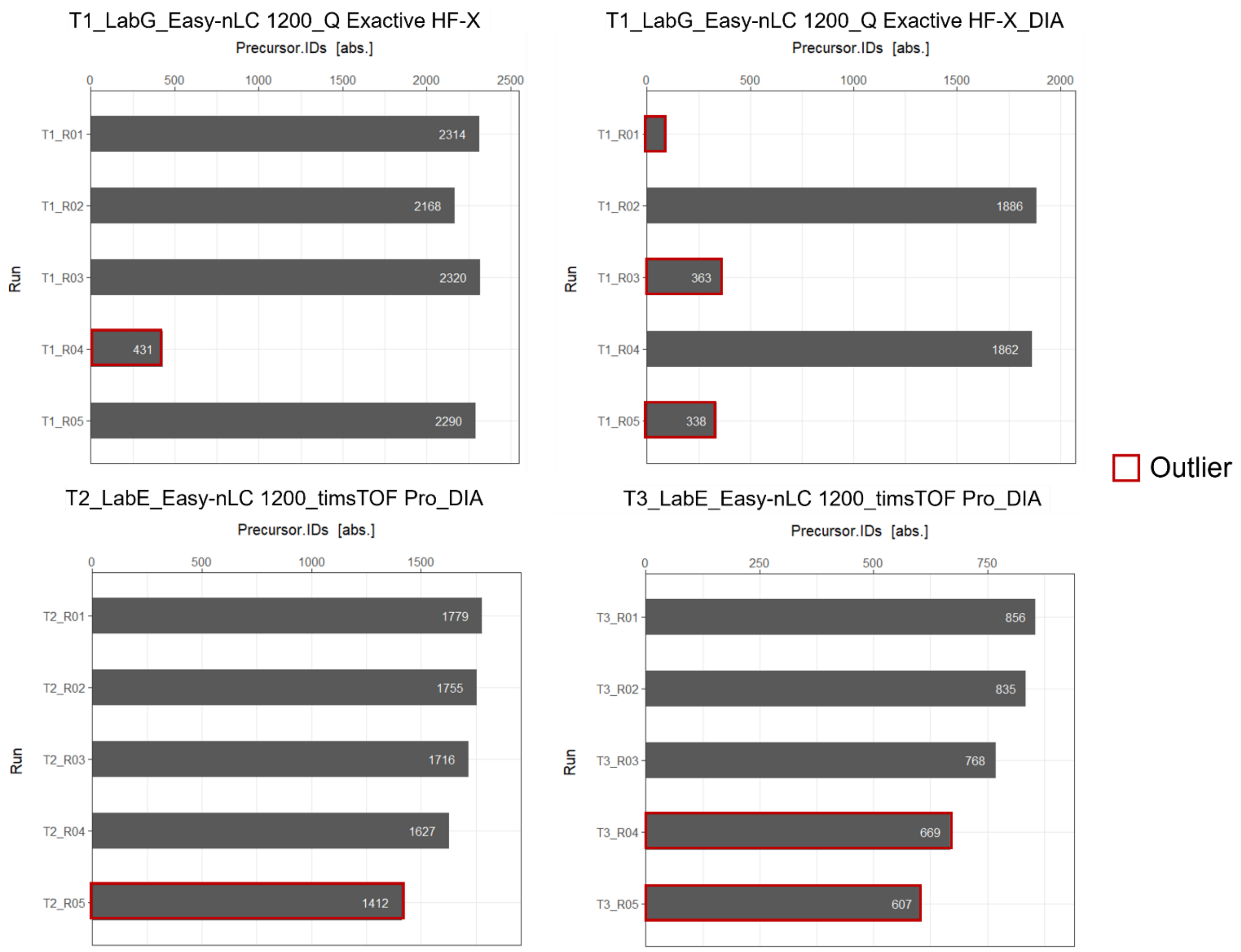

Fig. S2: Precursor IDs for plasma datasets in which outliers were detected. Outliers are highlighted in red.

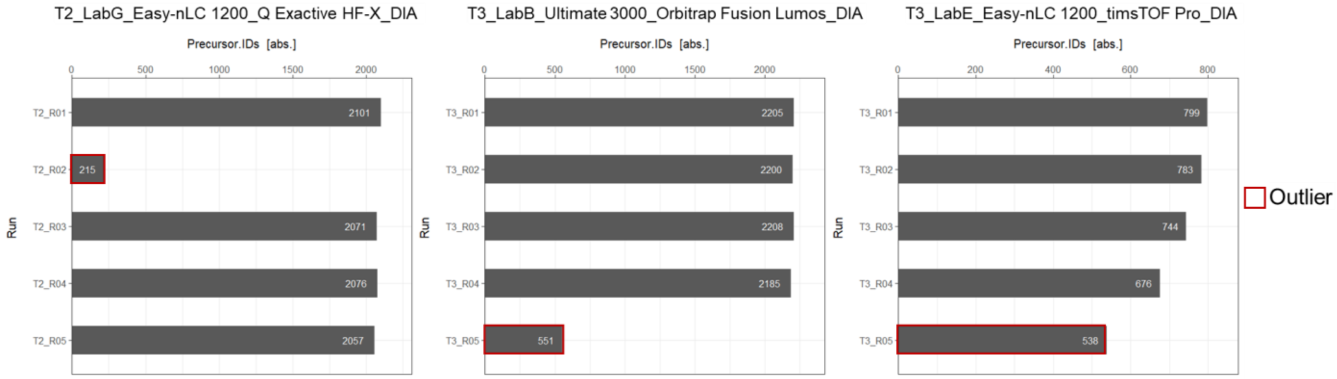

Fig. S3: Precursor IDs for serum datasets in which outliers were detected. Outliers are highlighted in red.

3. Principle component analysis

3.1 Plasma – all datasets

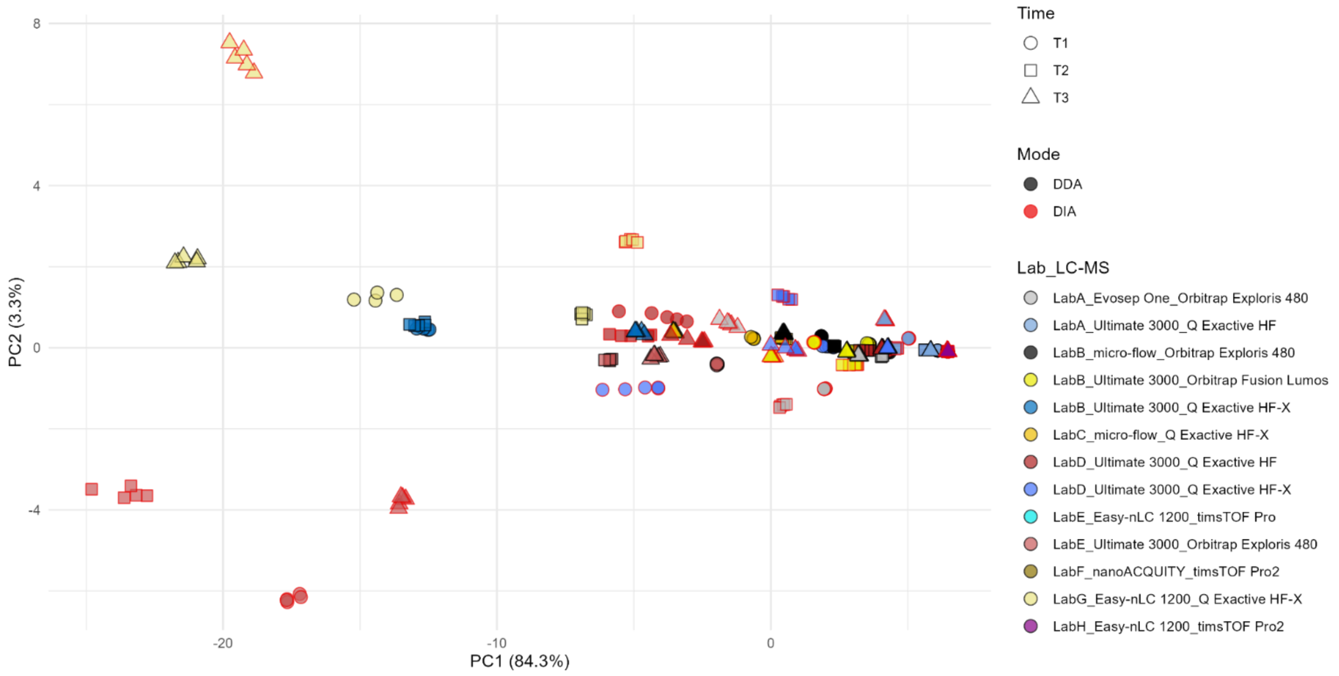

Fig. S4: Principal component analysis of all plasma datasets. Color coded by Laboratory – LC-MS set up. Timepoint is depicted in different shapes and the border of the shapes is color coded by acquisition mode.

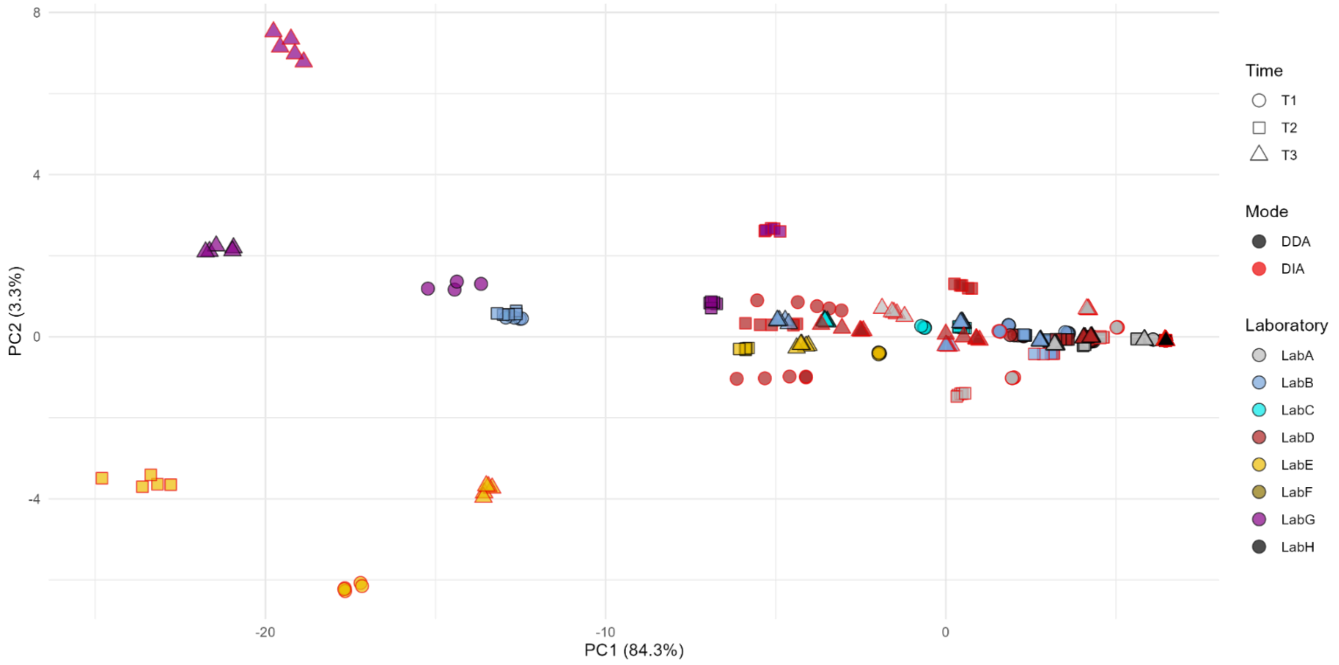

Fig. S5: Principal component analysis of all plasma datasets. Color coded by Laboratory. Timepoint is depicted in different shapes and the border of the shapes is color coded by acquisition mode.

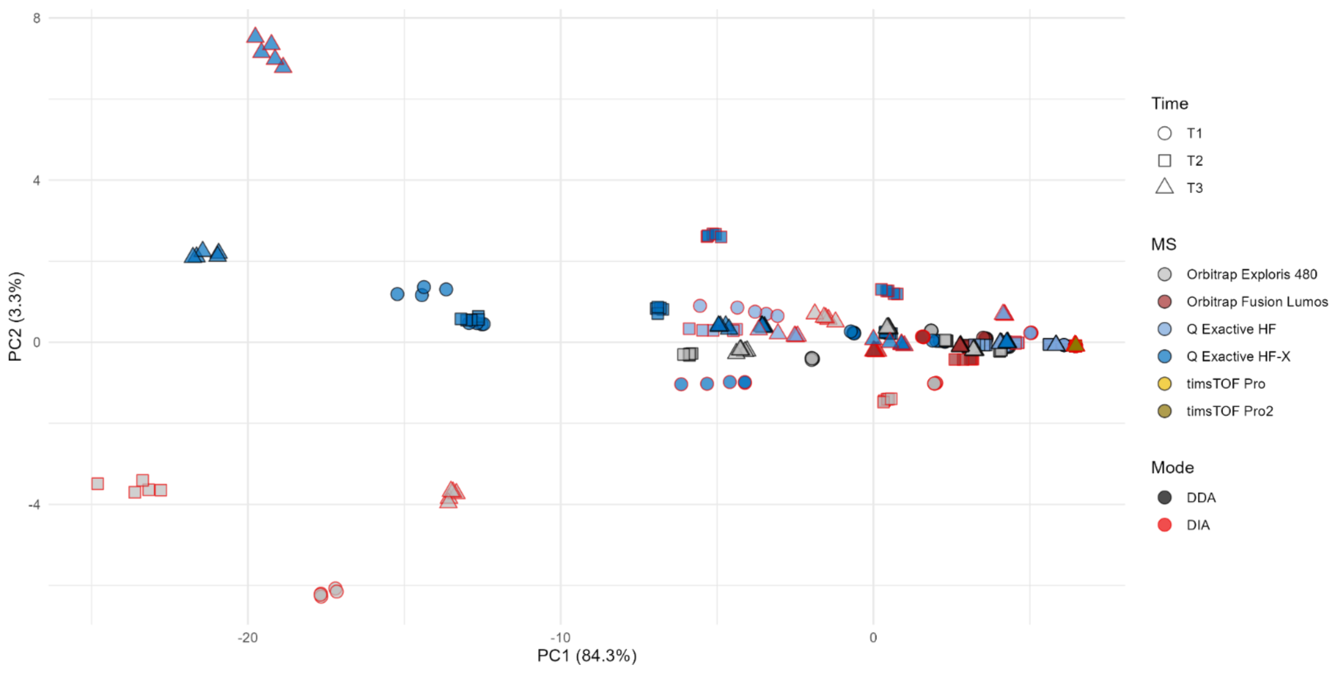

Fig. S6: Principal component analysis of all plasma datasets. Color coded by MS. Timepoint is depicted in different shapes and the border of the shapes is color coded by acquisition mode.

3.2 Plasma - timsTOF

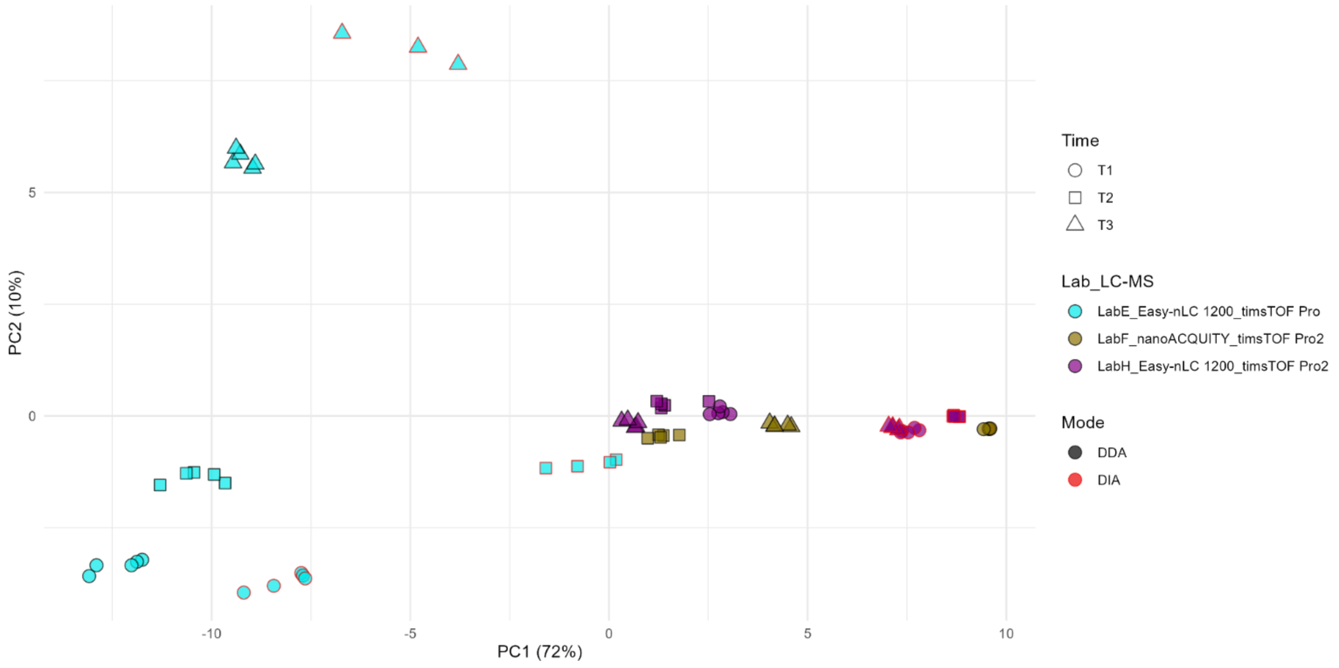

Fig. S7: Principal component analysis of all plasma datasets with timsTOF MS. Color coded by Laboratory – LC-MS set up. Timepoint is depicted in different shapes and the border of the shapes is color coded by acquisition mode.

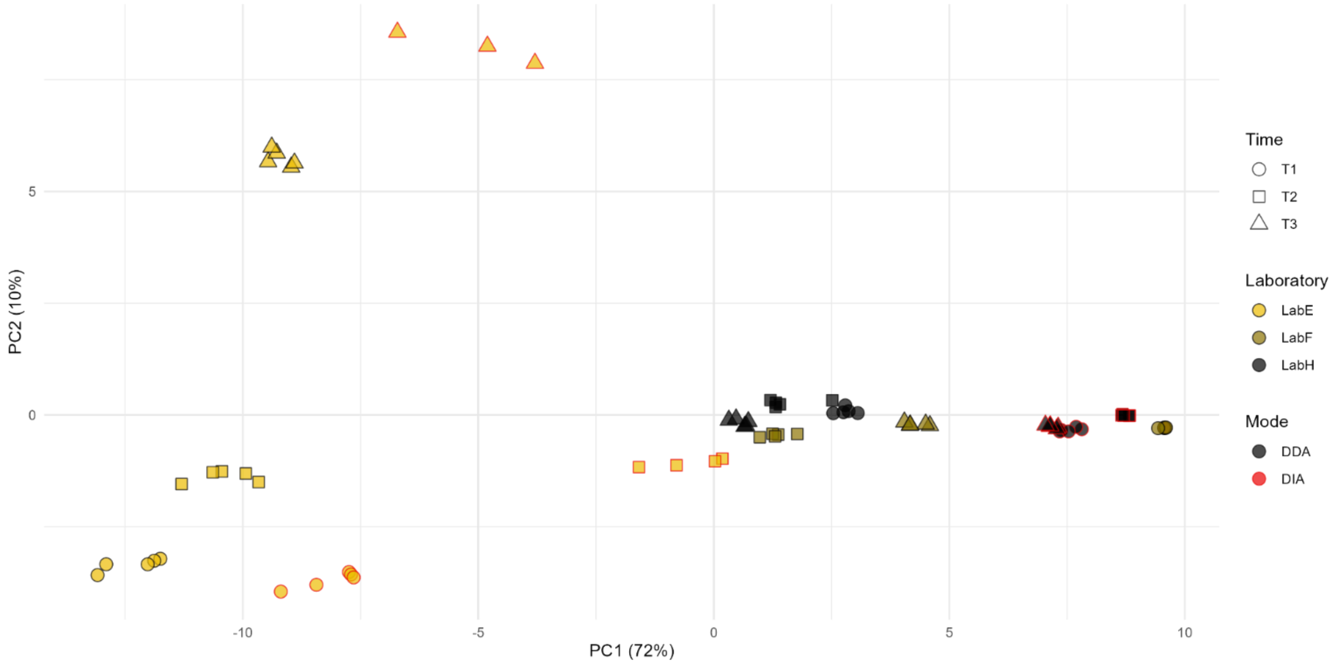

Fig. S8: Principal component analysis of all plasma datasets measured on timsTOF instruments. Color coded by

Laboratory. Timepoint is depicted in different shapes and the border of the shapes is color coded by acquisition mode.

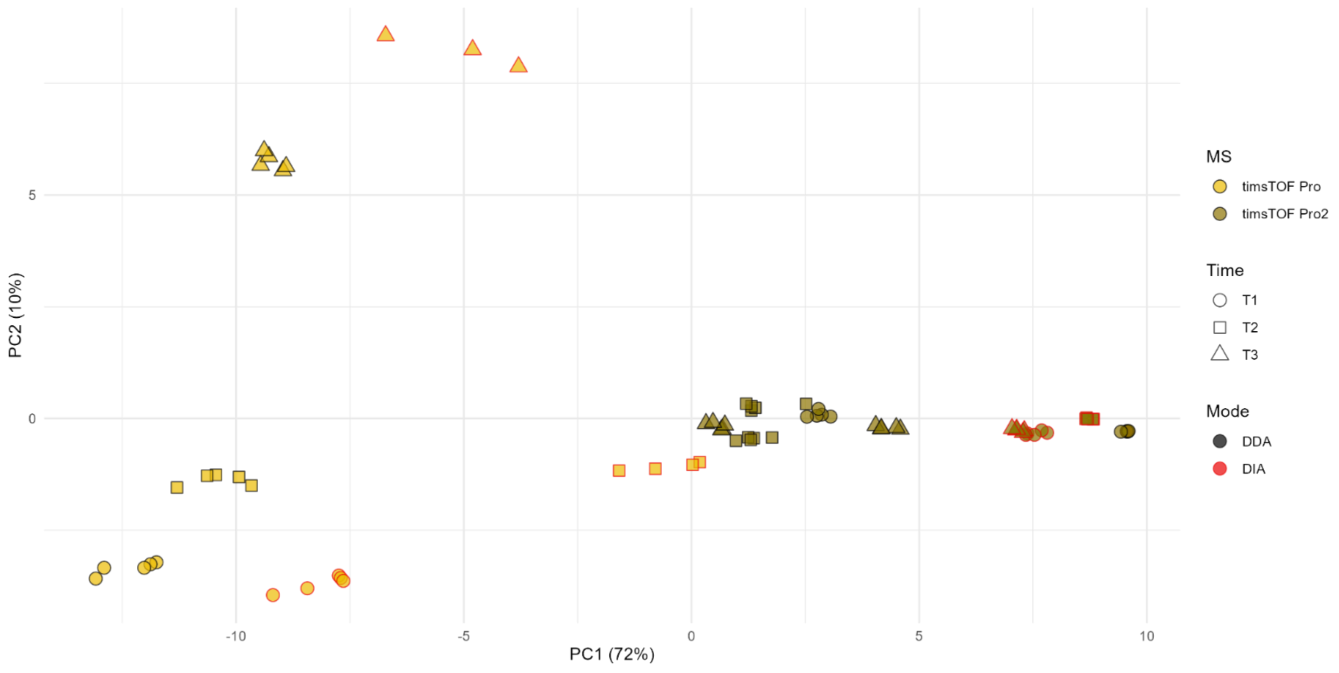

Fig. S9: Principal component analysis of all plasma datasets measured on timsTOF instruments. Color coded by MS. Timepoint is depicted in different shapes and the border of the shapes is color coded by acquisition mode.

3.3 Plasma - Orbitrap

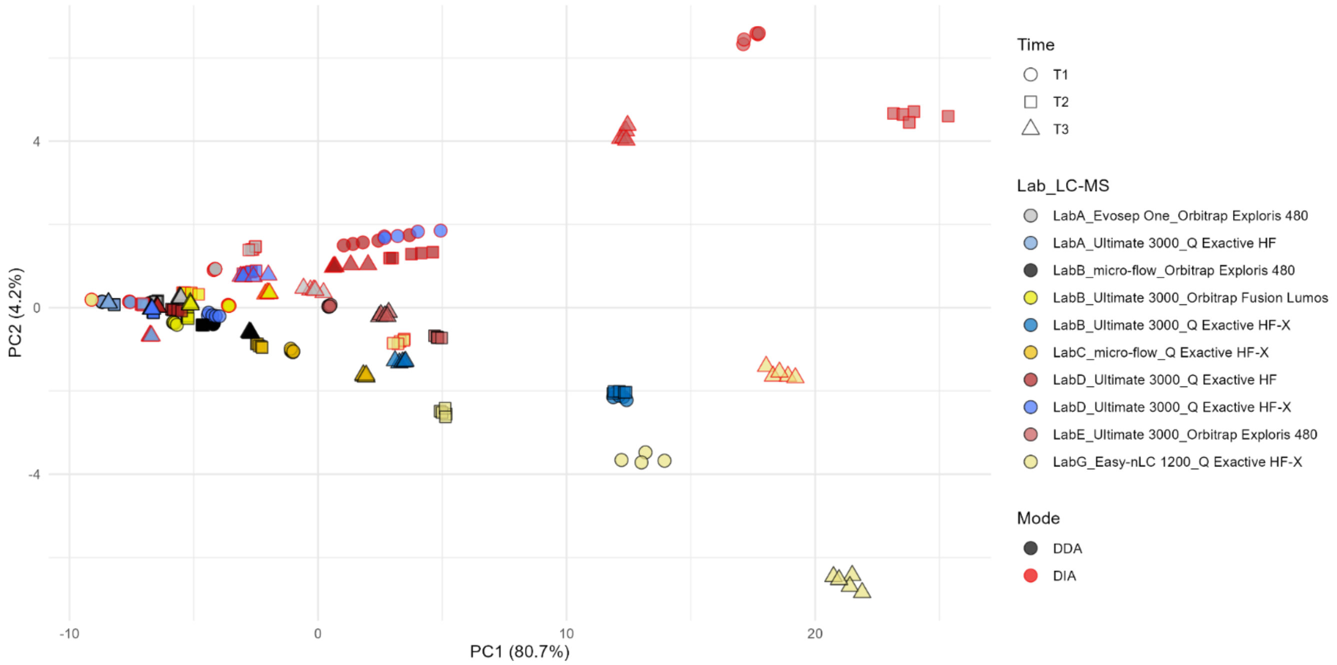

Fig. S10: Principal component analysis of all plasma datasets measured on orbitrap instruments. Color coded by Laboratory – LC-MS set up. Timepoint is depicted in different shapes and the border of the shapes is color coded by acquisition mode.

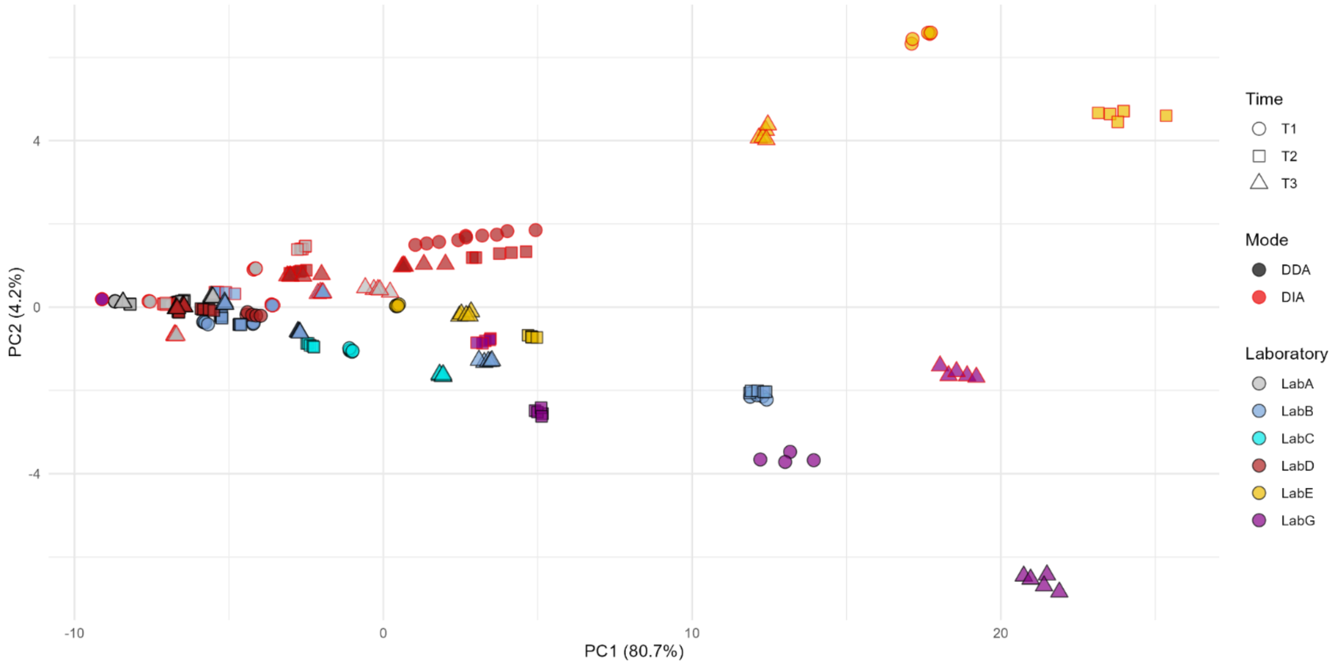

Fig. S11: Principal component analysis of all plasma datasets measured on orbitrap instruments. Color coded by Laboratory. Timepoint is depicted in different shapes and the border of the shapes is color coded by acquisition mode.

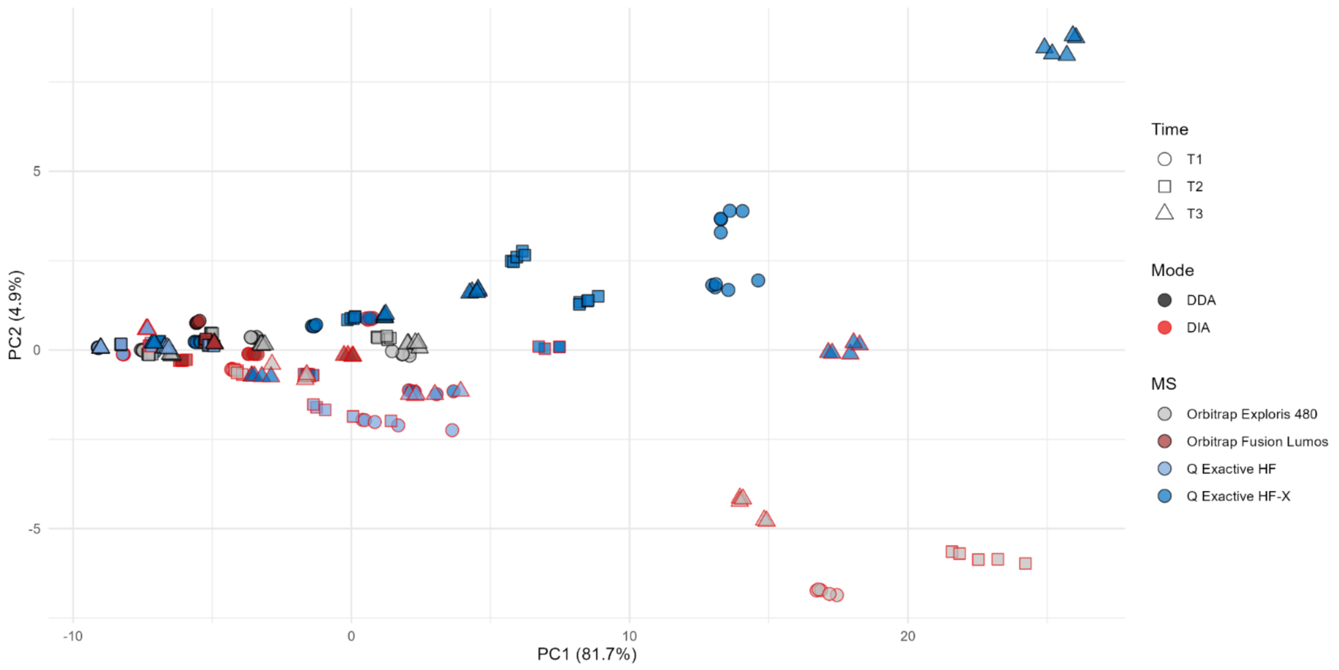

Fig. S12: Principal component analysis of all plasma datasets measured on orbitrap instruments. Color coded by MS. Timepoint is depicted in different shapes and the border of the shapes is color coded by acquisition mode.

3.4 Serum – all datasets

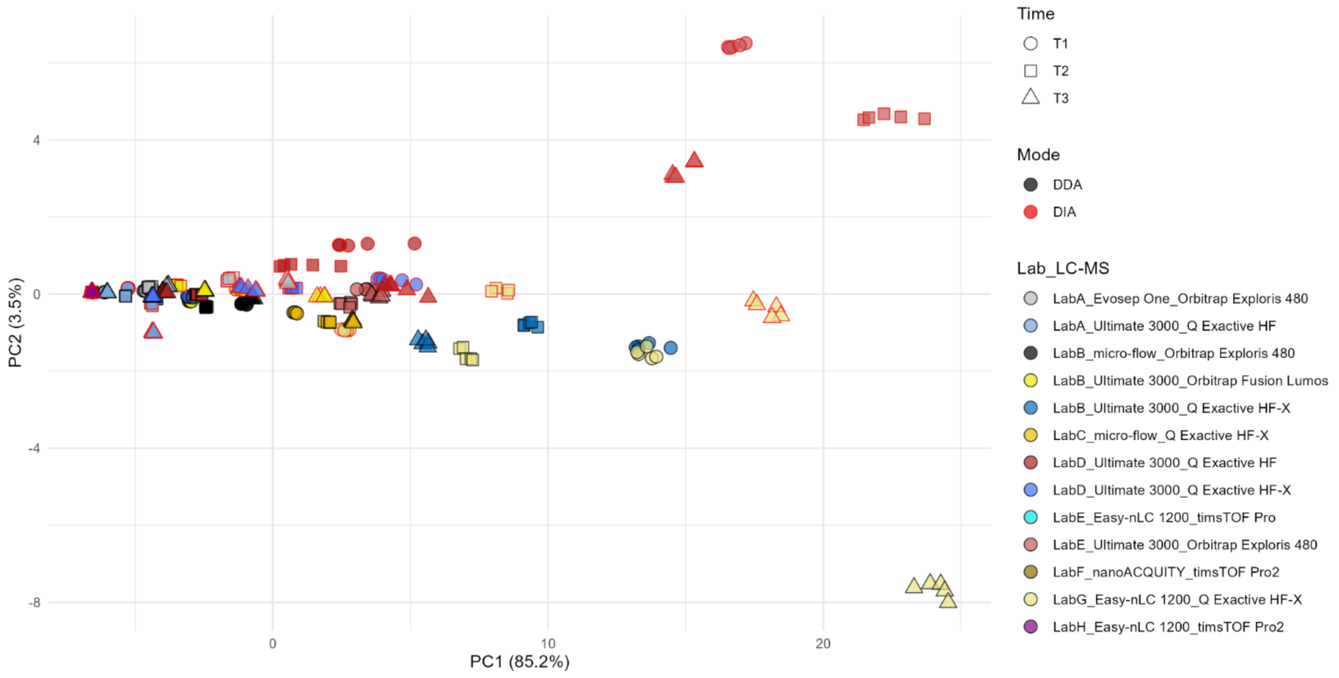

Fig. S13: Principal component analysis of all serum datasets. Color coded by Laboratory – LC-MS set up. Timepoint is depicted in different shapes and the border of the shapes is color coded by acquisition mode.

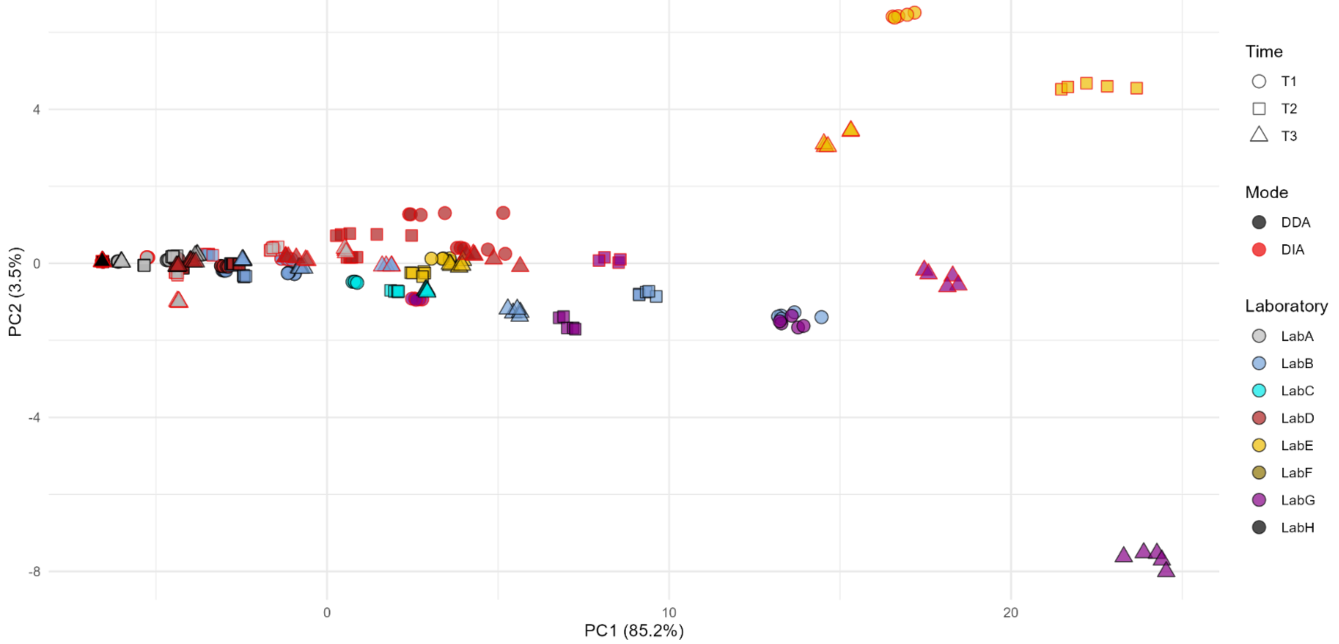

Fig. S14: Principal component analysis of all serum datasets. Color coded by Laboratory. Timepoint is depicted in different shapes and the border of the shapes is color coded by acquisition mode.

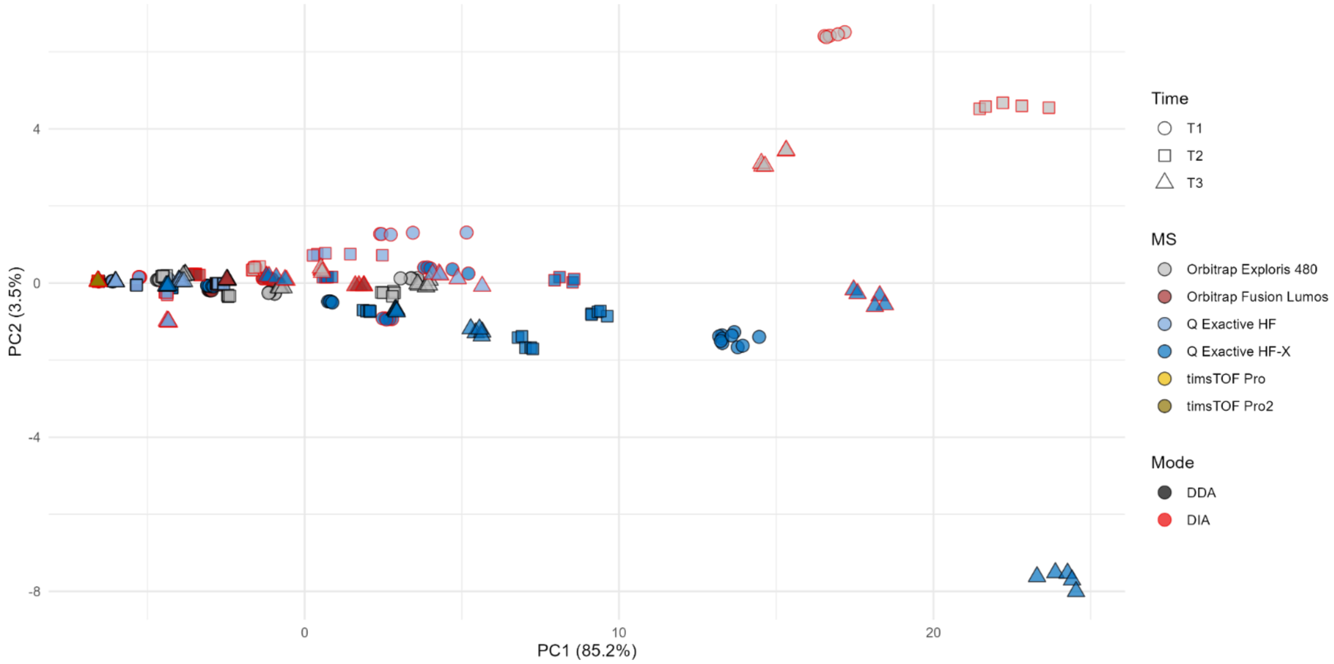

Fig. S15: Principal component analysis of all serum datasets. Color coded by MS. Timepoint is depicted in different shapes and the border of the shapes is color coded by acquisition mode.

3.5 Serum – timsTOF

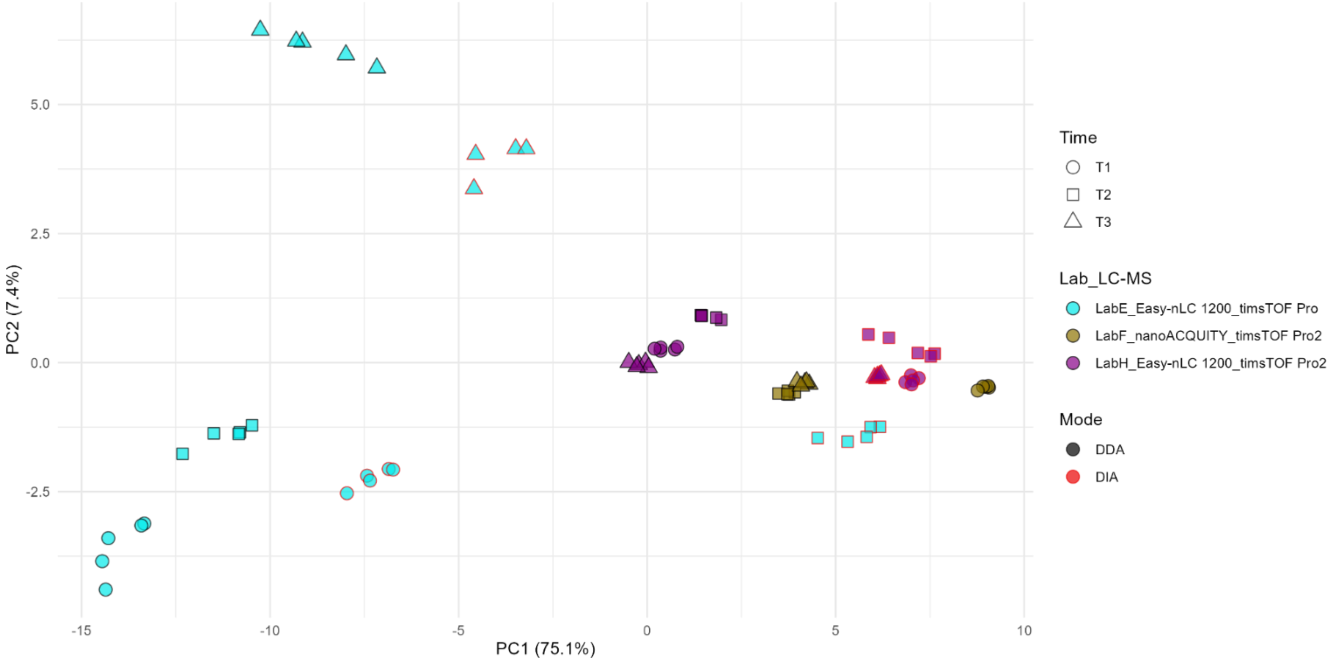

Fig. S16: Principal component analysis of all serum datasets measured on timsTOF instruments. Color coded by Laboratory – LC-MS set up. Timepoint is depicted in different shapes and the border of the shapes is color coded by acquisition mode.

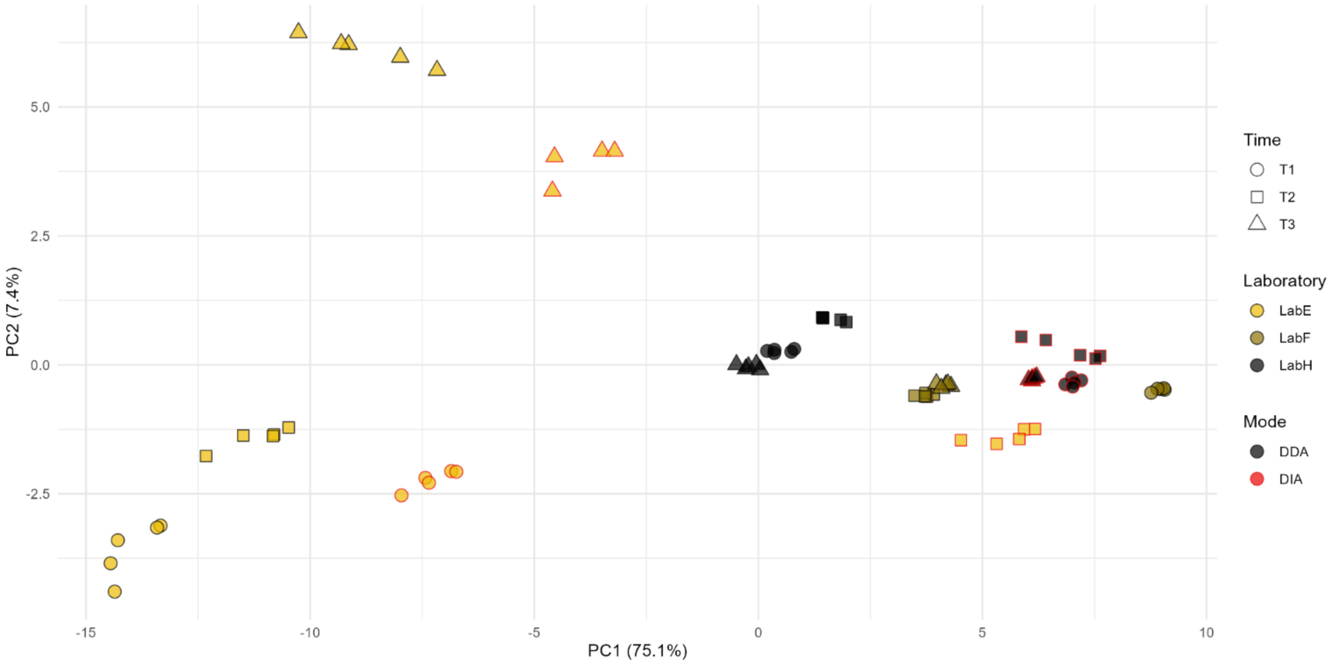

Fig. S17: Principal component analysis of all serum datasets measured on timsTOF instruments. Color coded by Laboratory. Timepoint is depicted in different shapes and the border of the shapes is color coded by acquisition mode.

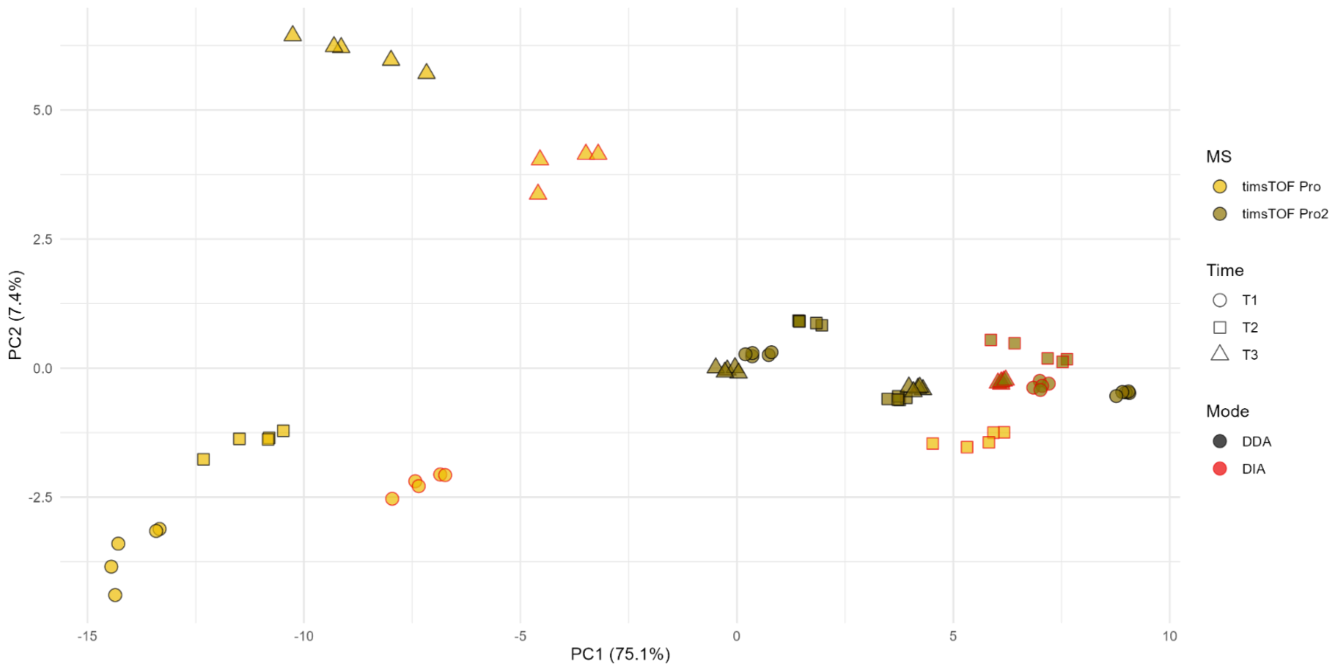

Fig. S18: Principal component analysis of all serum datasets measured on timsTOF instruments. Color coded by MS. Timepoint is depicted in different shapes and the border of the shapes is color coded by acquisition mode.

3.6 Serum - Orbitrap

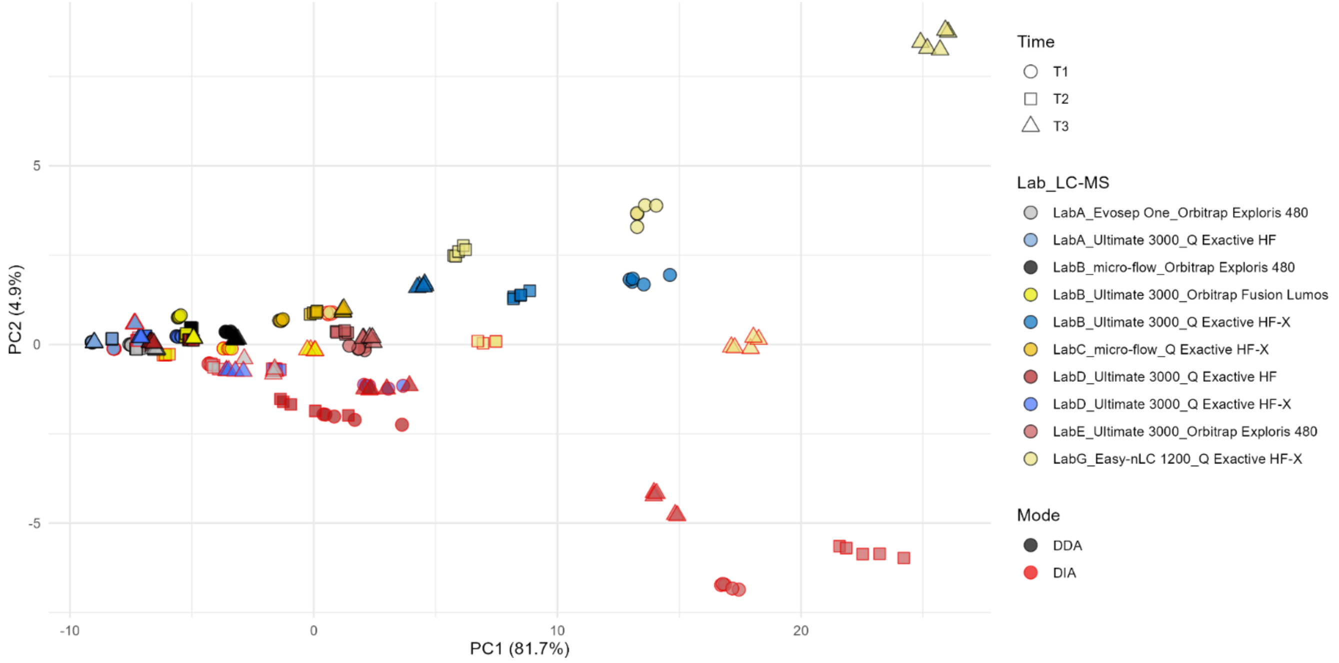

Fig. S19: Principal component analysis of all serum datasets measured on orbitrap instruments. Color coded by Laboratory – LC-MS set up. Timepoint is depicted in different shapes and the border of the shapes is color coded by acquisition mode.

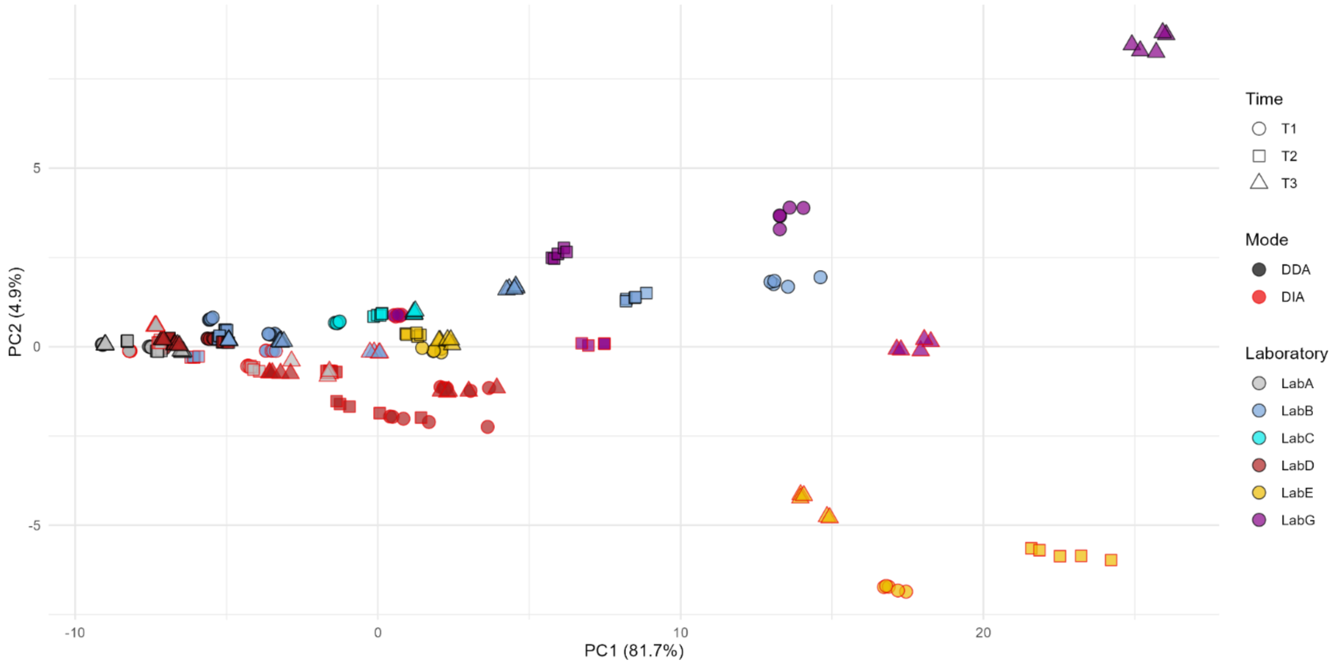

Fig. S20: Principal component analysis of all serum datasets measured on orbitrap instruments. Color coded by Laboratory. Timepoint is depicted in different shapes and the border of the shapes is color coded by acquisition mode.

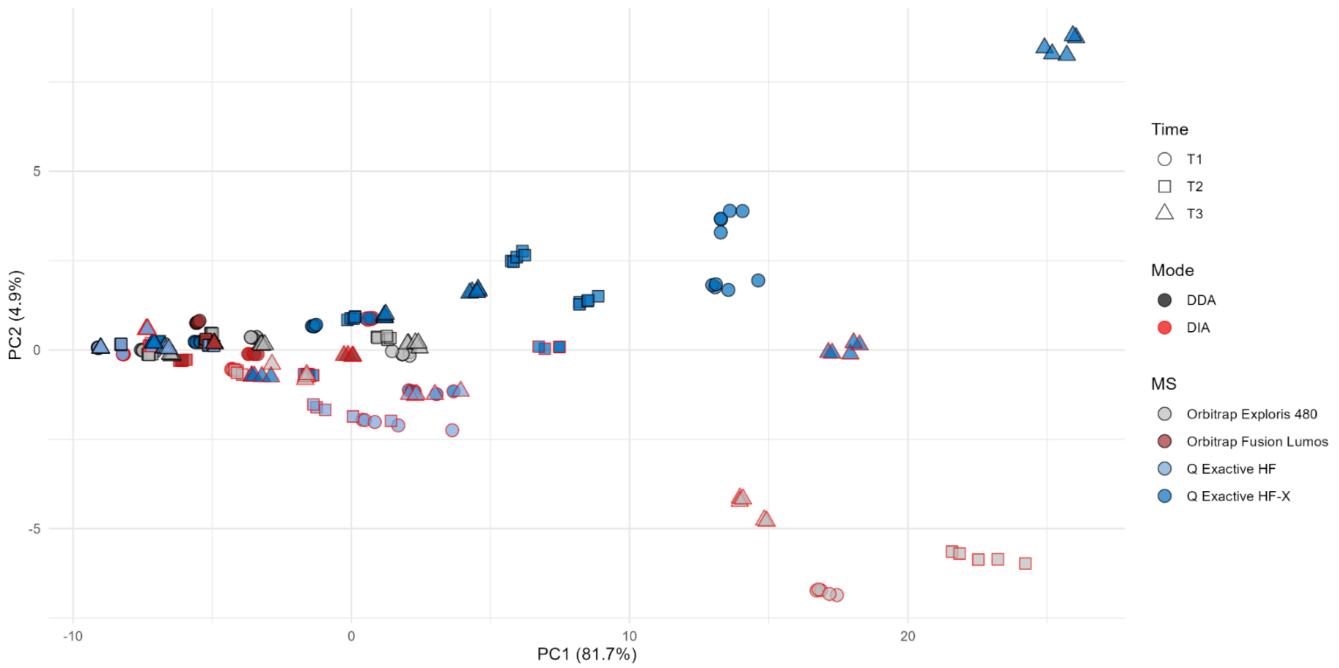

Fig. S21: Principal component analysis of all serum datasets measured on orbitrap instruments. Color coded by MS. Timepoint is depicted in different shapes and the border of the shapes is color coded by acquisition mode.

4. Identifications

4.1 Protein group-level

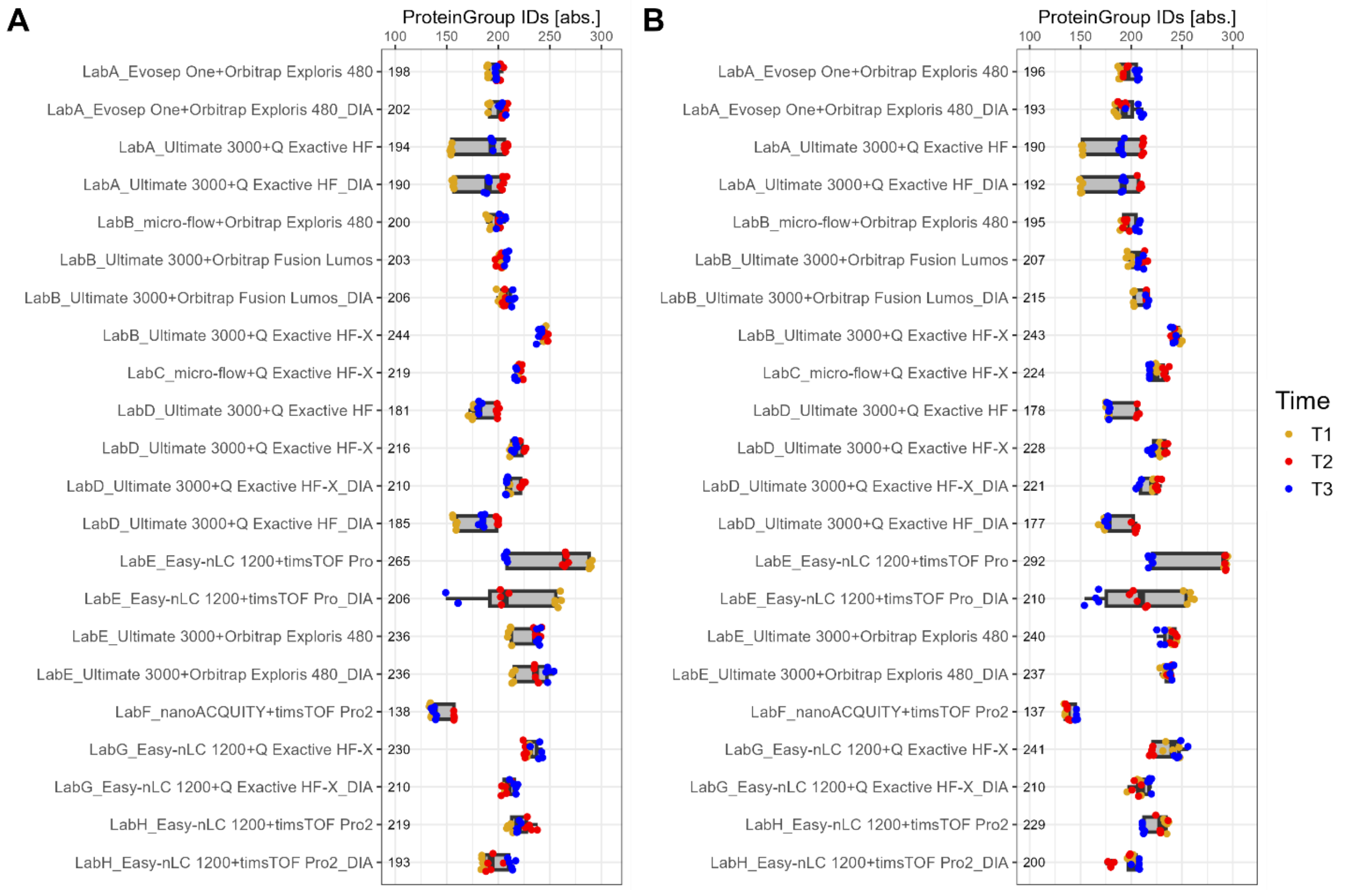

Fig. S22: Protein group IDs for plasma (A) and serum (B) for different set ups. Measurements are color coded by timepoints. Median protein group IDs per set up is shown as label.

4.2 Peptide-level

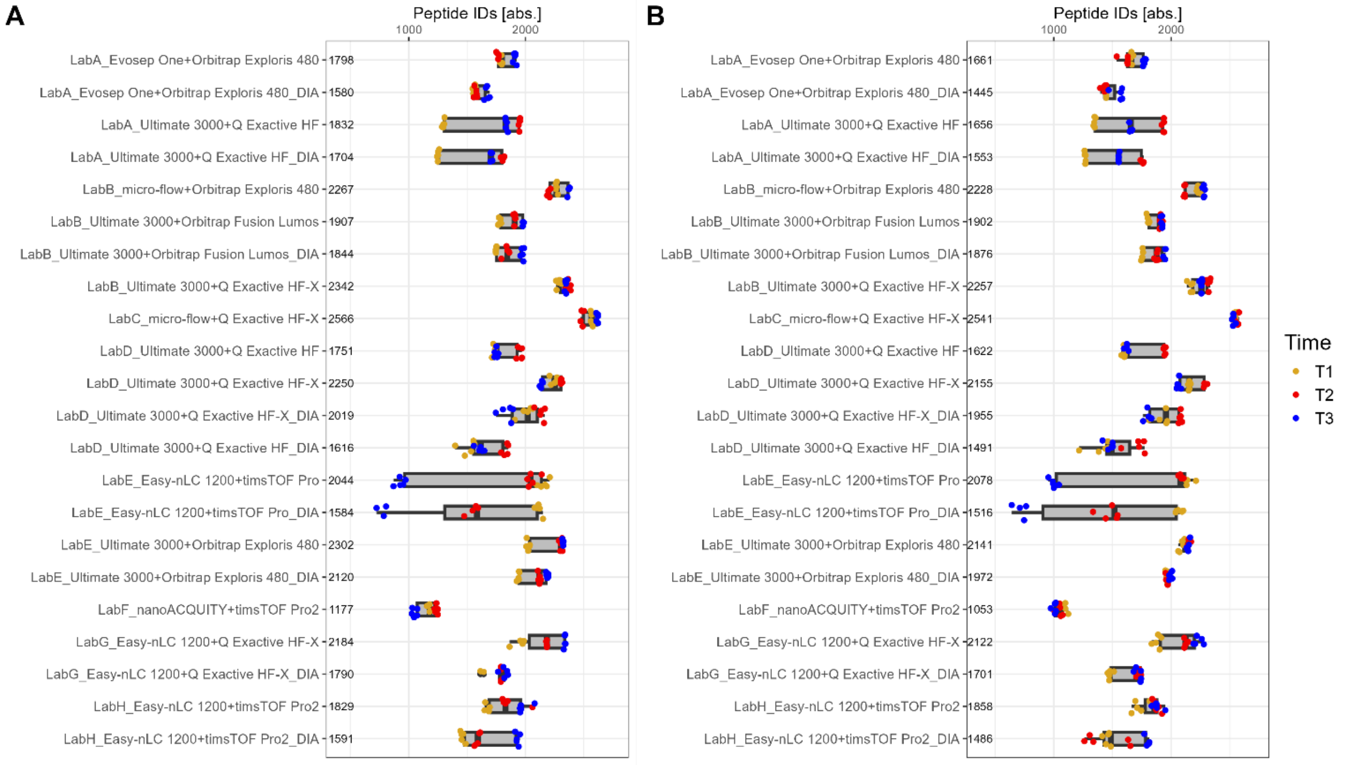

Fig. S23: Peptide IDs for plasma (A) and serum (B) for different set ups. Measurements are color coded by timepoints. Median peptide IDs per set up is shown as label.

4.3 Precursor-level

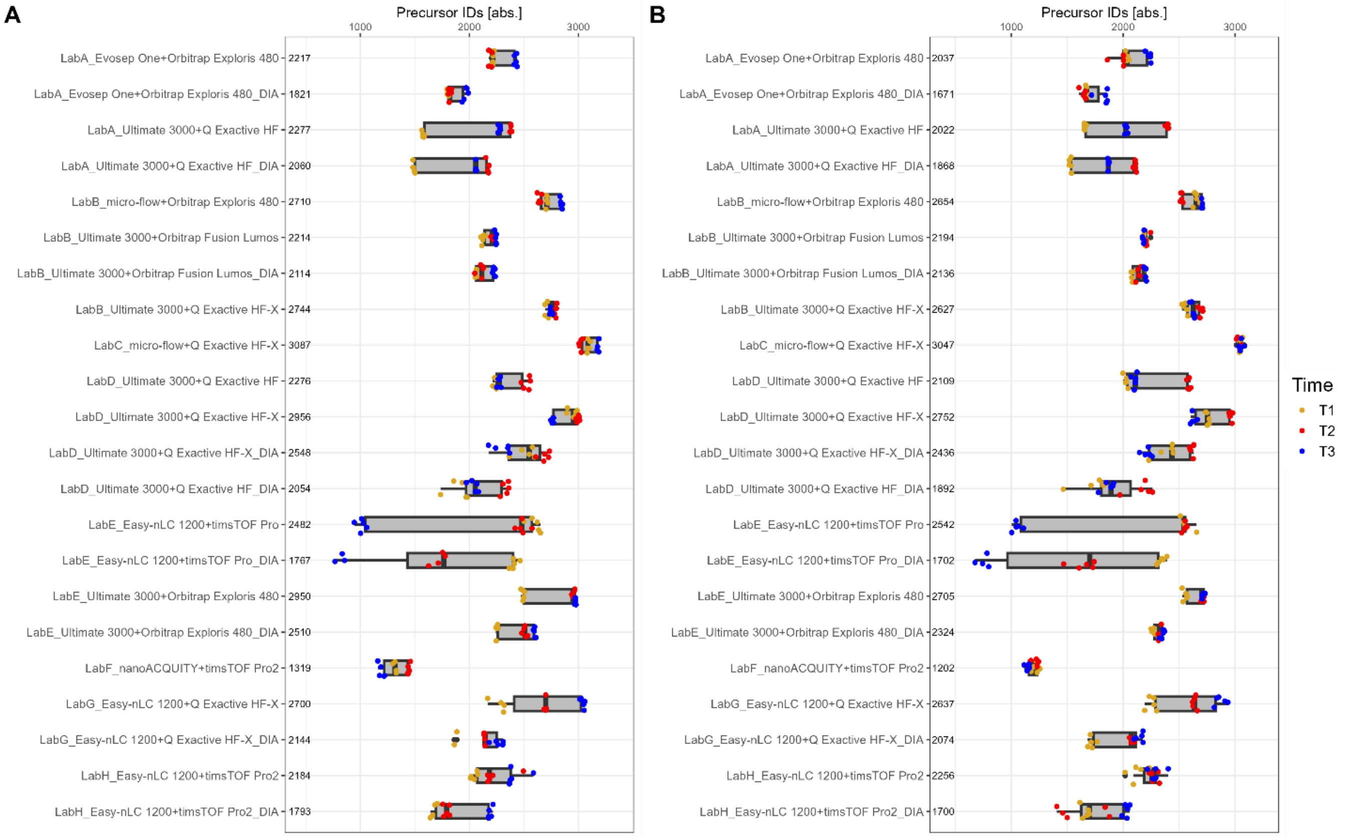

Fig. S24: Precursor IDs for plasma (A) and serum (B) for different set ups. Measurements are color coded by timepoints. Median precursor IDs per set up is shown as label.

5. Data Completeness

5.1 Protein-level: relative DC – T1 vs. T2 vs. T3

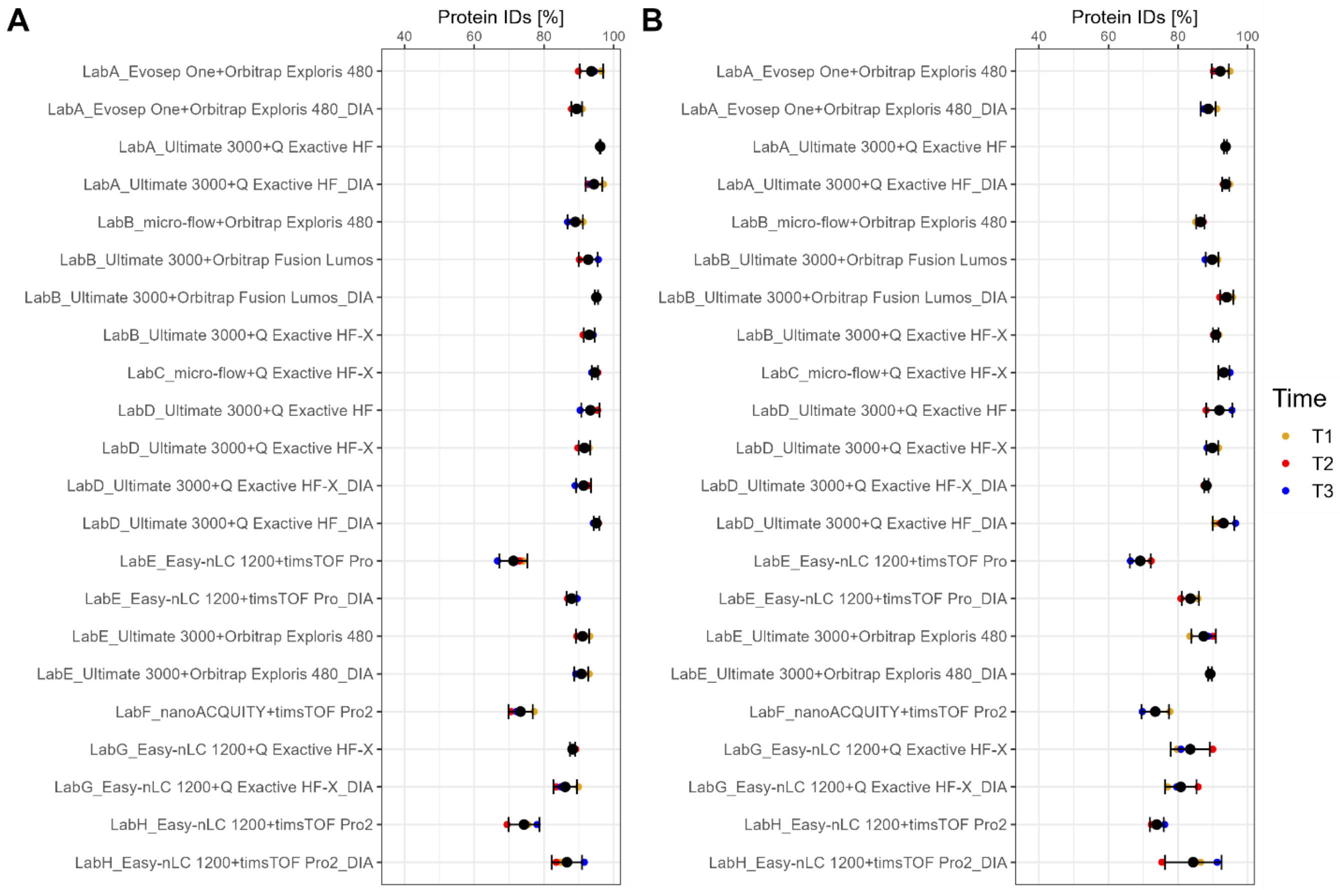

Fig. S25: Relative data Completeness [%] on protein-level for plasma (A) and serum (B) for different set ups. Only full profiles are displayed, and color coded by different timepoints. Mean and standard deviation as error bars are plotted in black.

5.2 Protein-level: Mean and Standard Deviation

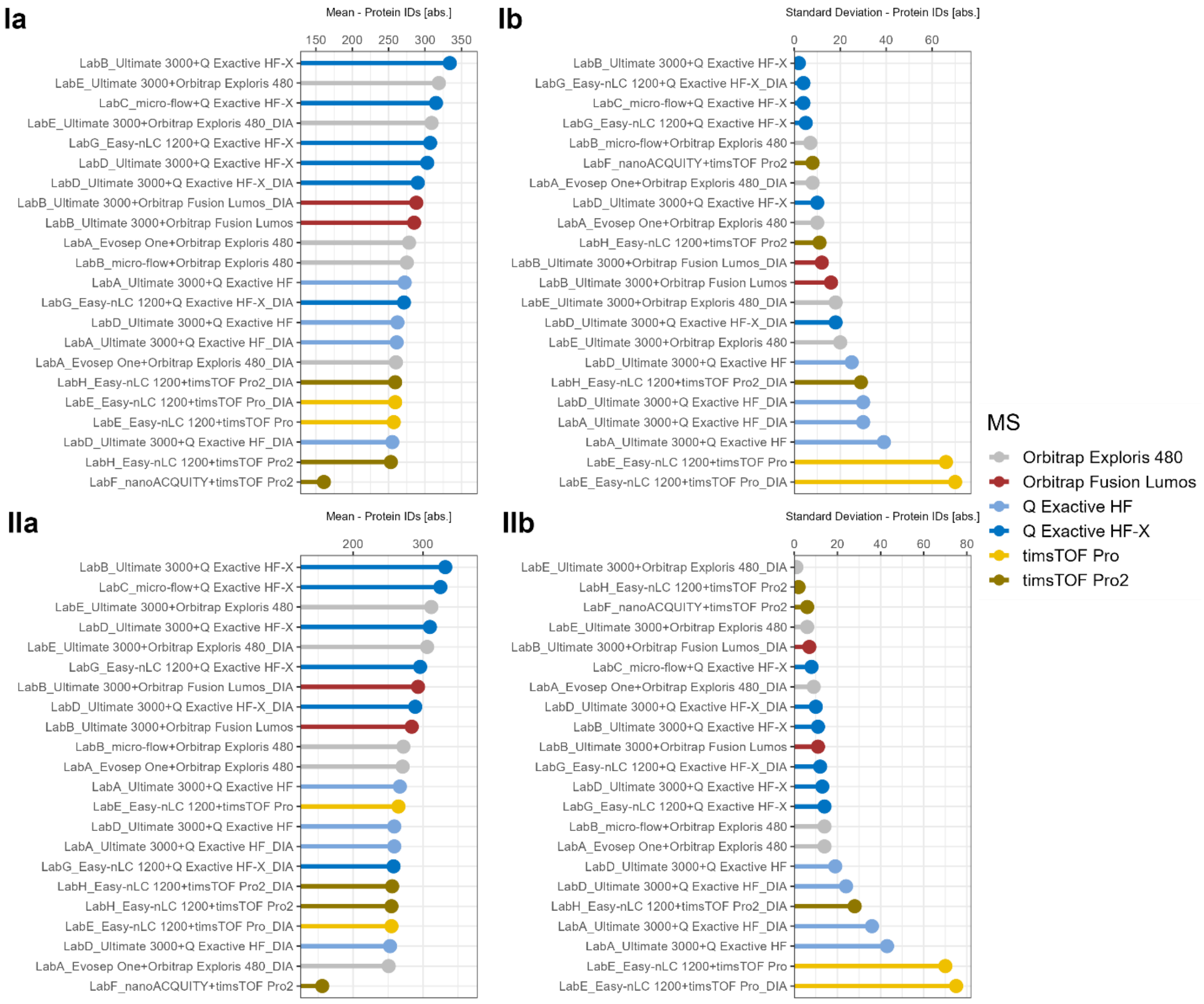

Fig. S26: Data completeness of full profiles [abs.] on protein-level - mean number of identifications in decreasing order (a) and standard deviation in increasing order (b) on protein-level for plasma (I) and serum (II). Results are color coded by MS instrument.

5.3 Protein group-level: absolute DC – T1 vs. T2 vs. T3

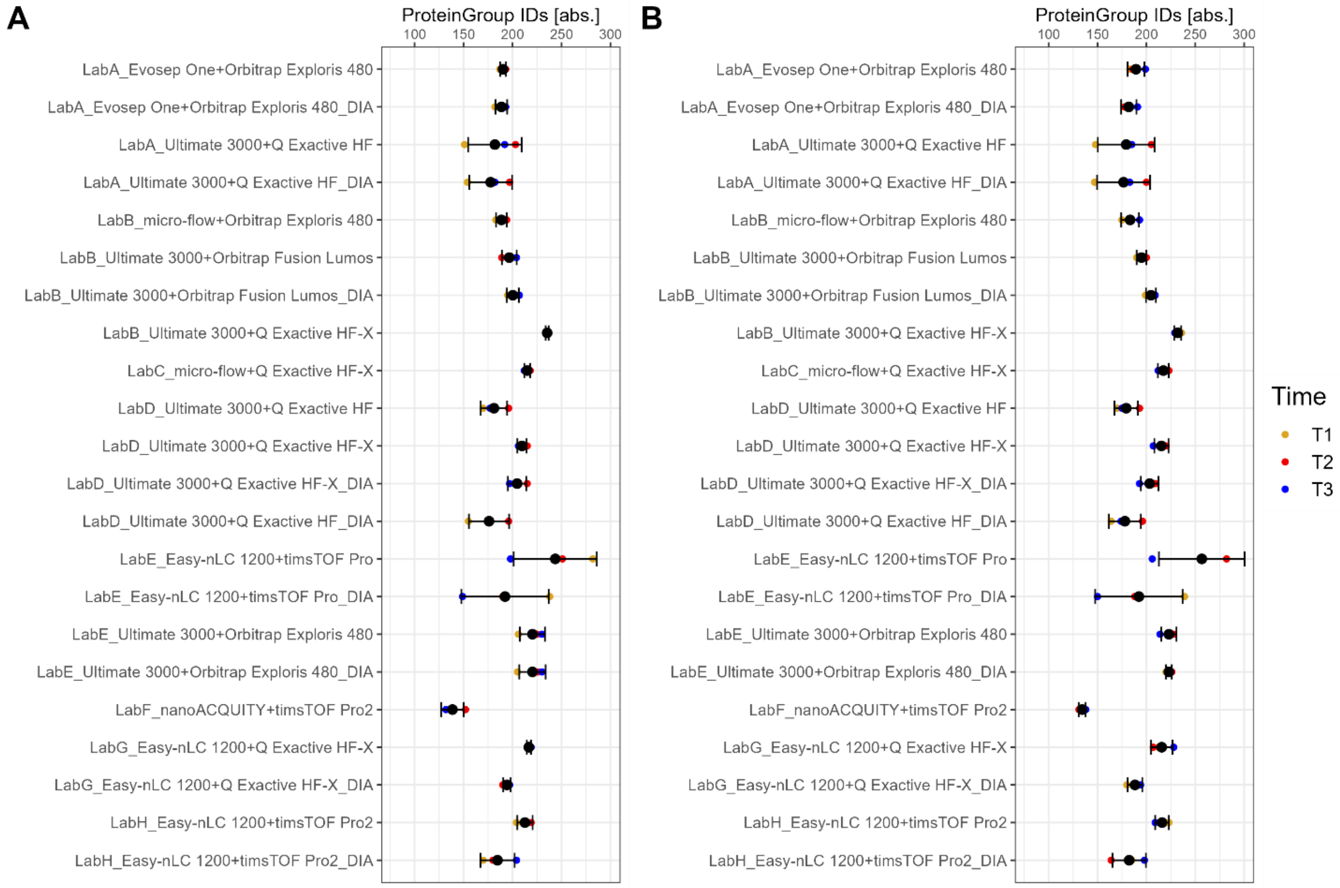

Fig. S27: Data Completeness [abs.] on protein group-level for plasma (A) and serum (B) for different set ups. Only full profiles are displayed, and color coded by different timepoints. Mean and standard deviation as error bars are plotted in black.

5.4 Peptide-level: absolute DC – T1 vs. T2 vs. T3

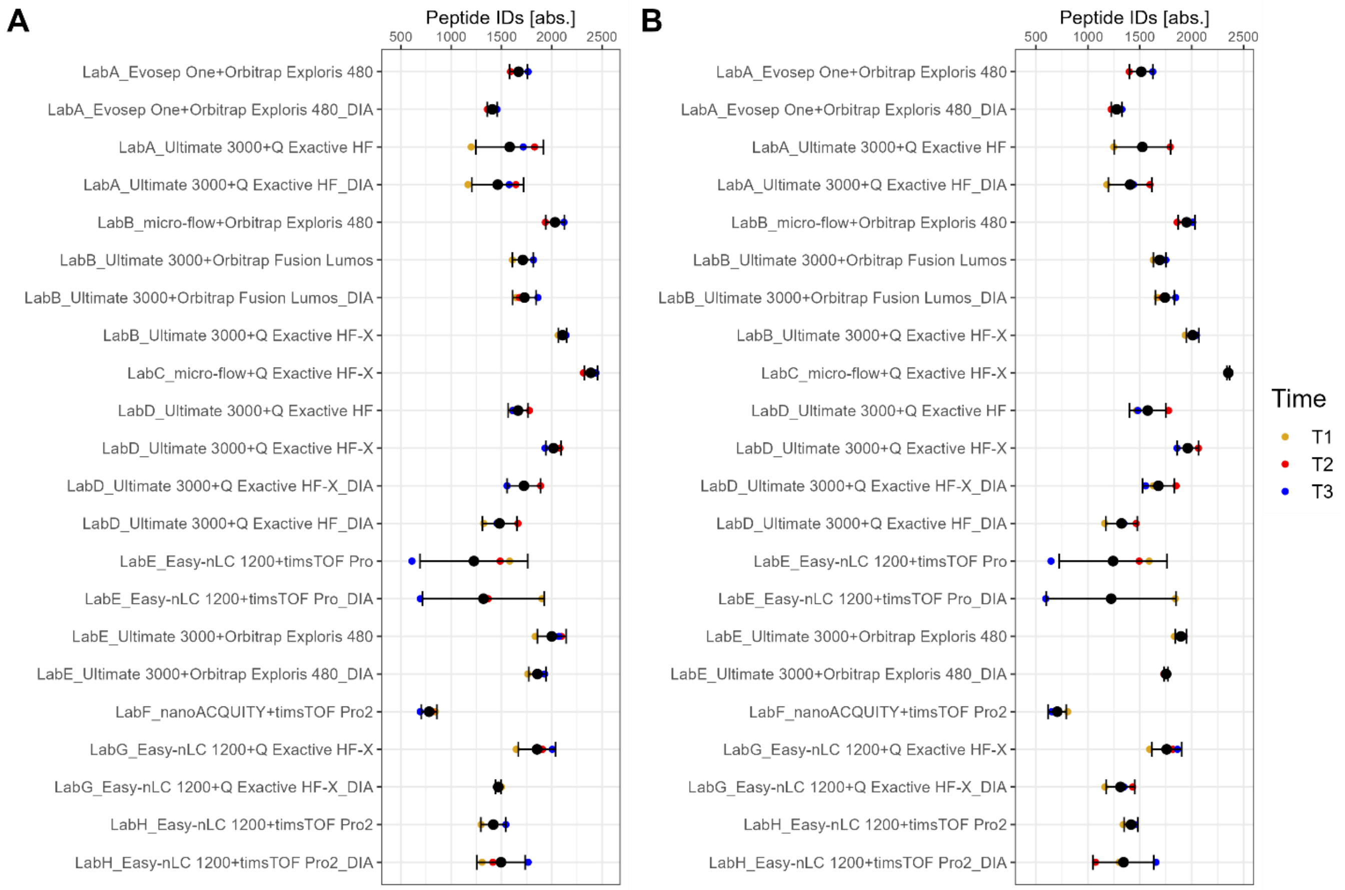

Fig. S28: Data Completeness [abs.] on peptide-level for plasma (A) and serum (B) for different set ups. Only full profiles are displayed, and color coded by different timepoints. Mean and standard deviation as error bars are plotted in black.

5.5 Precursor-level: absolute DC – T1 vs. T2 vs. T3

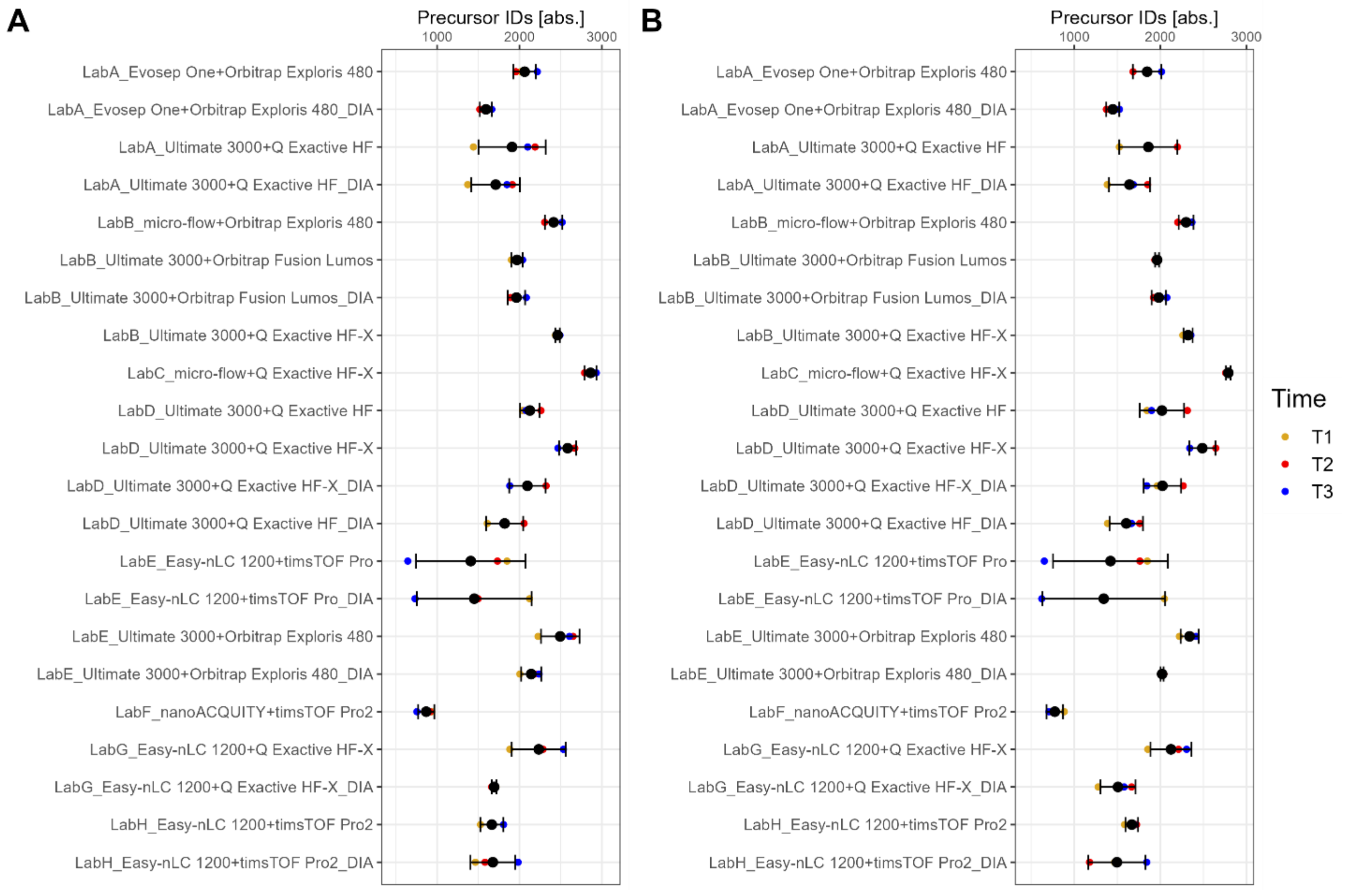

Fig. S29: Data Completeness [abs.] on precursor-level for plasma (A) and serum (B) for different set ups. Only full profiles are displayed, and color coded by different timepoints. Mean and standard deviation as error bars are plotted in black.

5.6 Protein-level: relative DC – T1 + T2 + T3

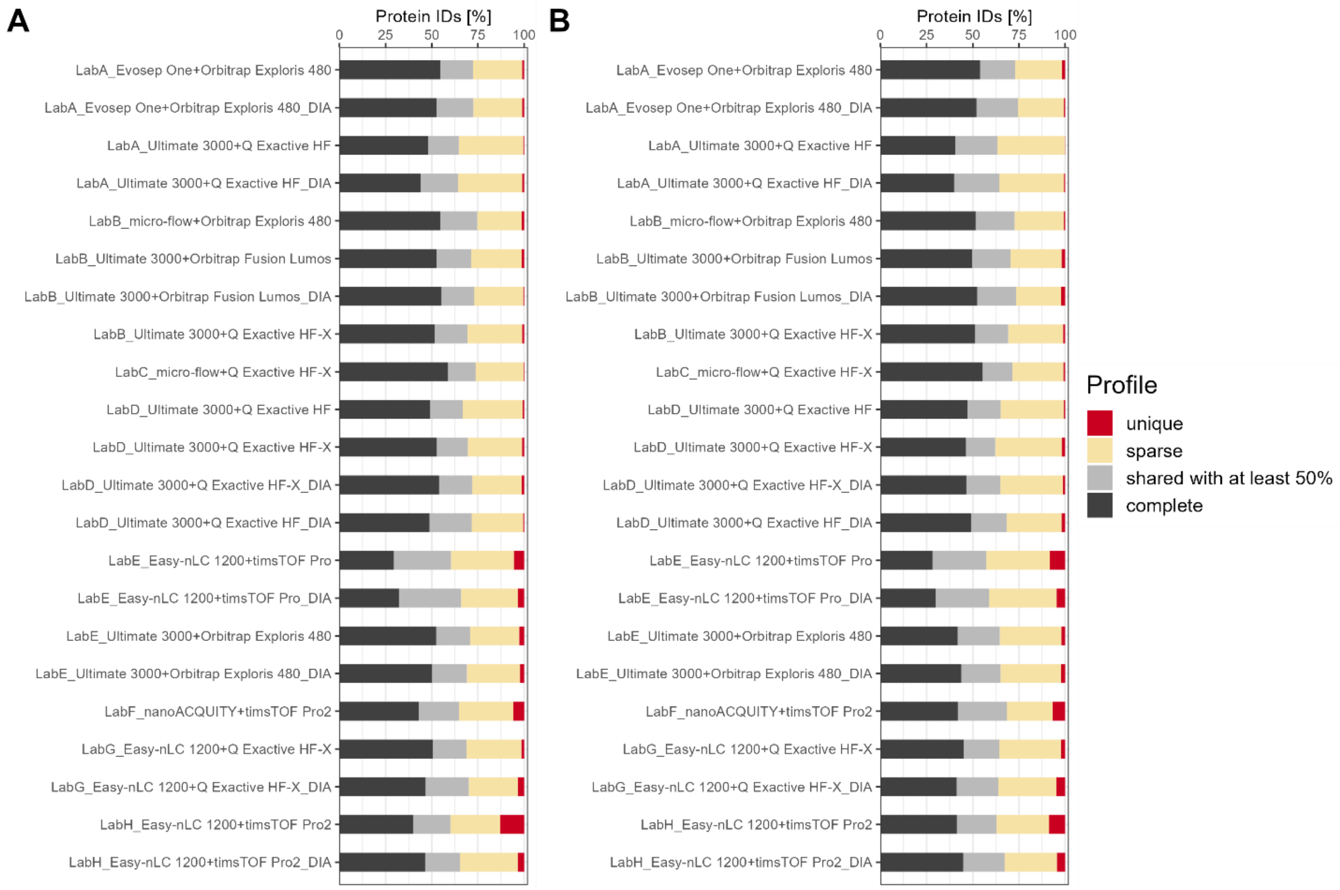

Fig. S30: Relative data completeness [%] on protein-level for plasma (A) and serum (B). Results of each timepoint are merged per set up.

6. Quantitative Precision

6.1 CV distribution

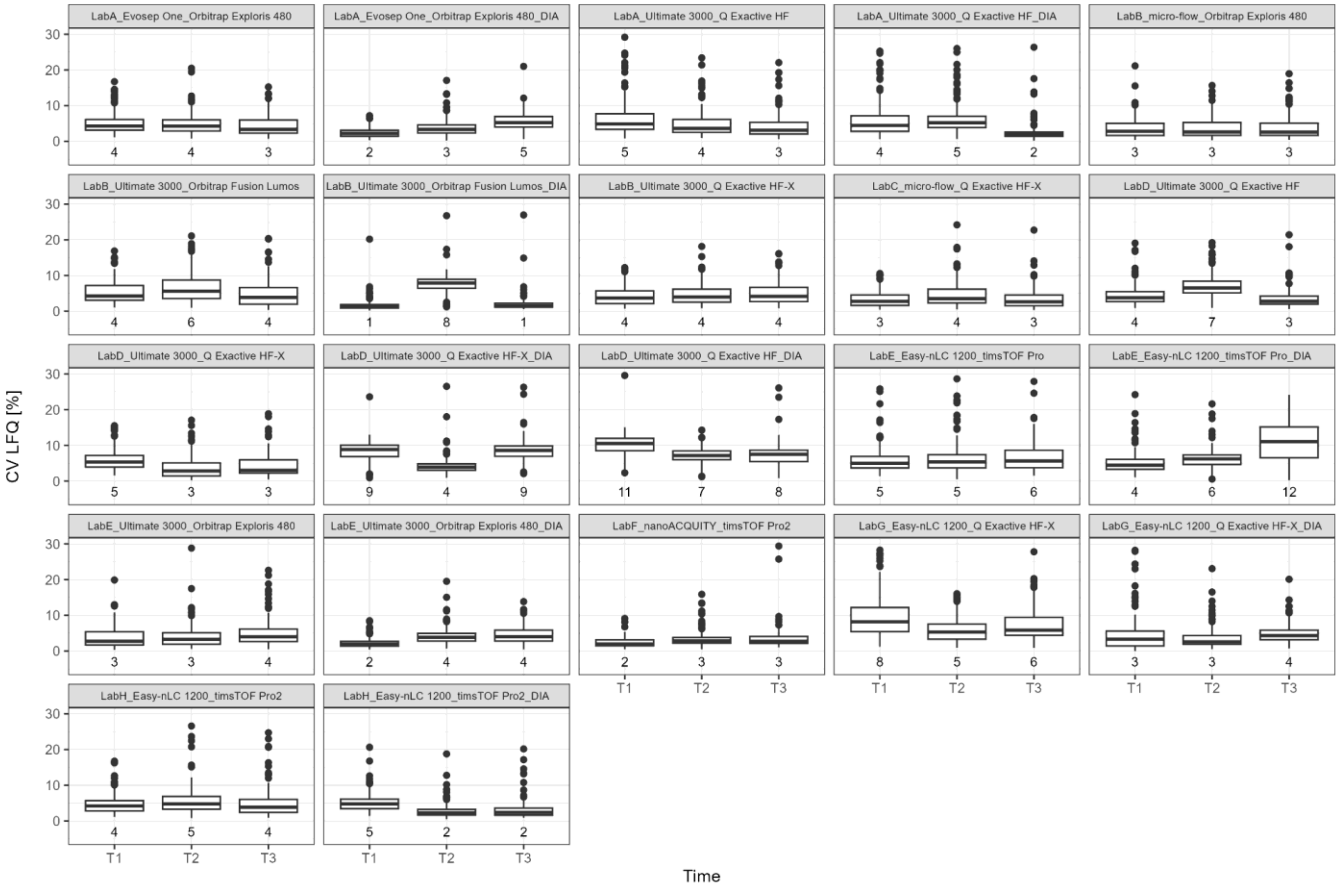

Fig. S31: CV distribution of all plasma datasets separated by lab and time points. Median CV [%] values are shown below boxplots. Y-axis is cut at 30% to aid data visualization.

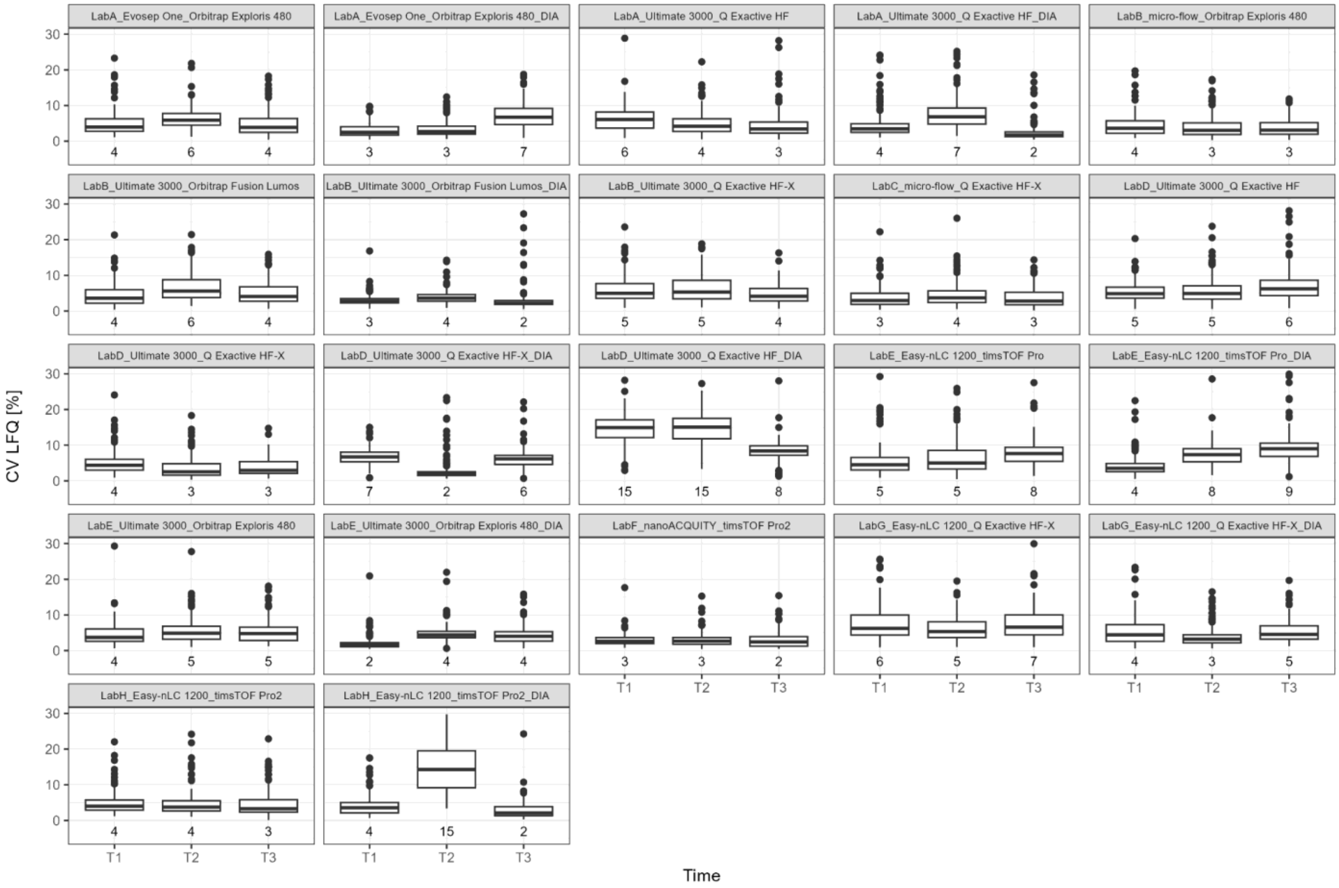

Fig. S32: CV distribution of all serum datasets separated by lab and time points. Median CV [%] values are shown below boxplots. Y-axis is cut at 30% to aid data visualization.

6.2 Plasma: CV vs. Intensity distribution binned

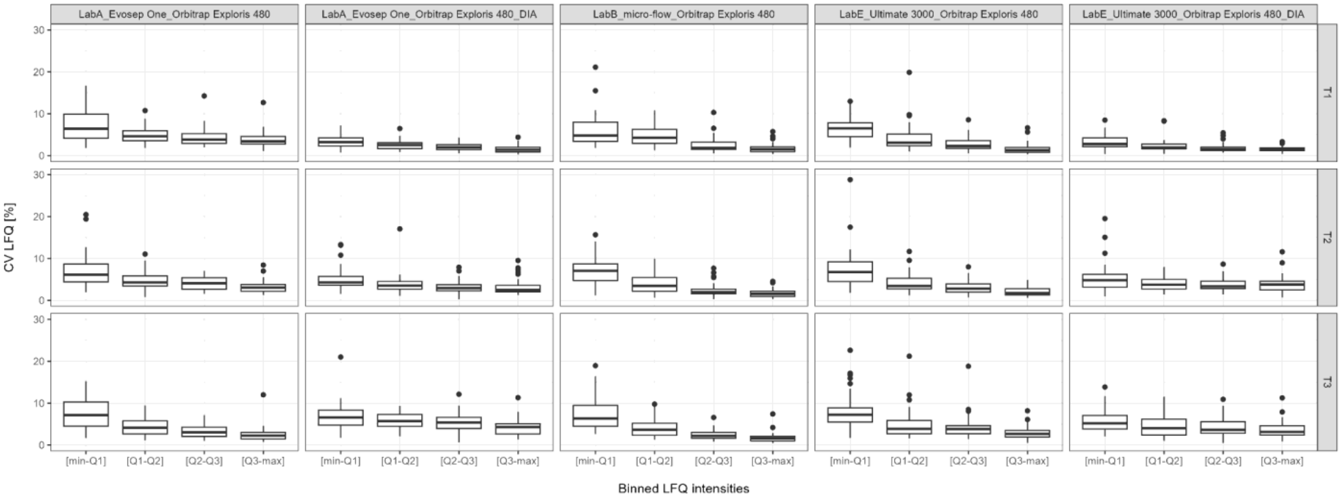

Fig. S33: CV distribution in respect to binned LFQ intensity distribution of all plasma datasets measured on Orbitrap Exploris 480 instruments. Y-axis is cut at 30% to aid data visualization.

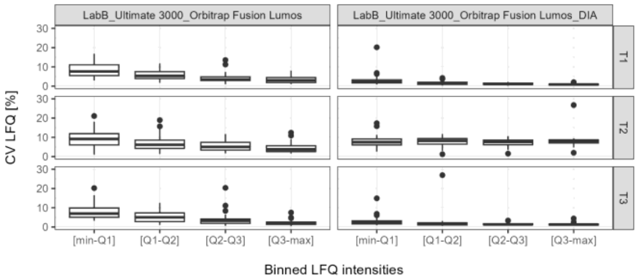

Fig. S34: CV distribution in respect to binned LFQ intensity distribution of all plasma datasets measured on Orbitrap Fusion Lumos instruments. Y-axis is cut at 30% to aid data visualization.

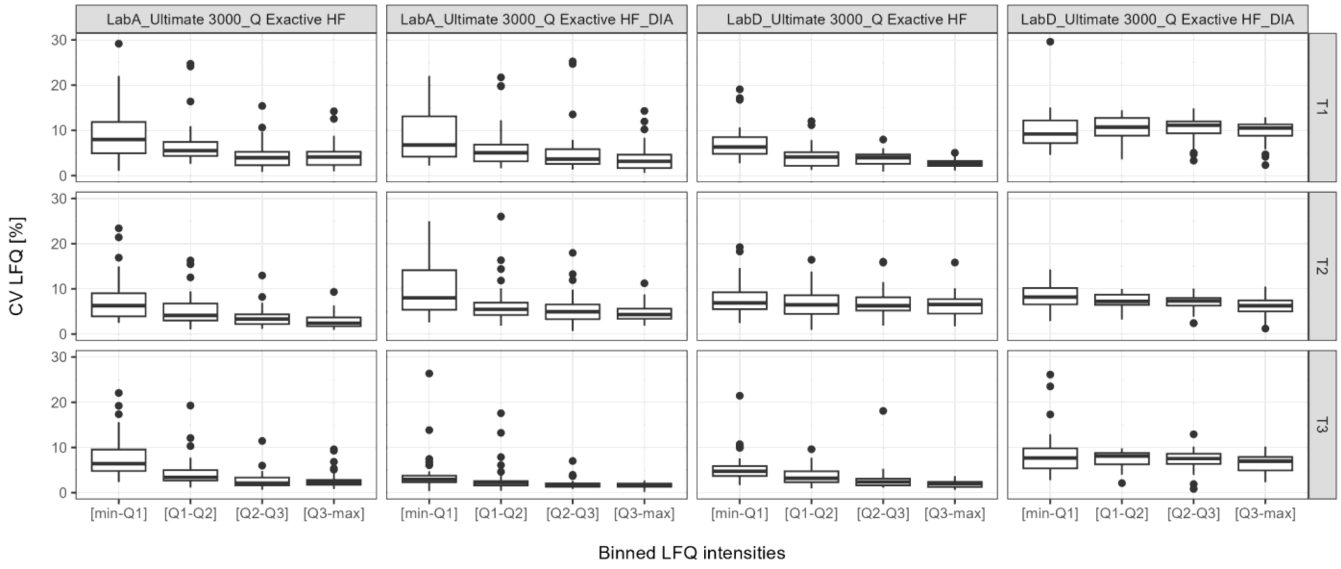

Fig. S35: CV distribution in respect to binned LFQ intensity distribution of all plasma datasets measured on Q Exactive HF instruments. Y-axis is cut at 30% to aid data visualization.

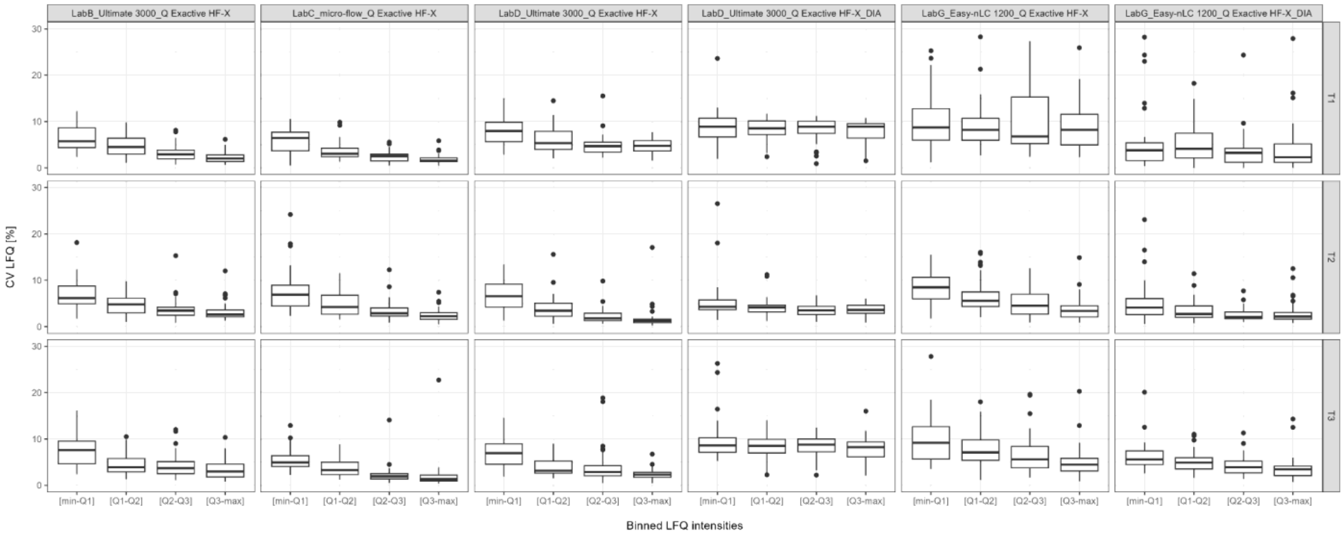

Fig. S36: CV distribution in respect to binned LFQ intensity distribution of all plasma datasets measured on Q Exactive HF-X instruments. Y-axis is cut at 30% to aid data visualization.

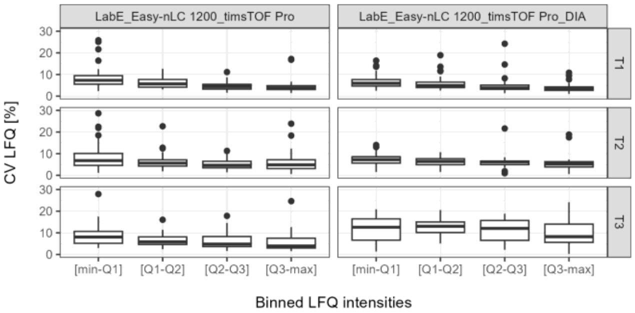

Fig. S37: CV distribution in respect to binned LFQ intensity distribution of all plasma datasets measured on timsTOF Pro instruments. Y-axis is cut at 30% to aid data visualization.

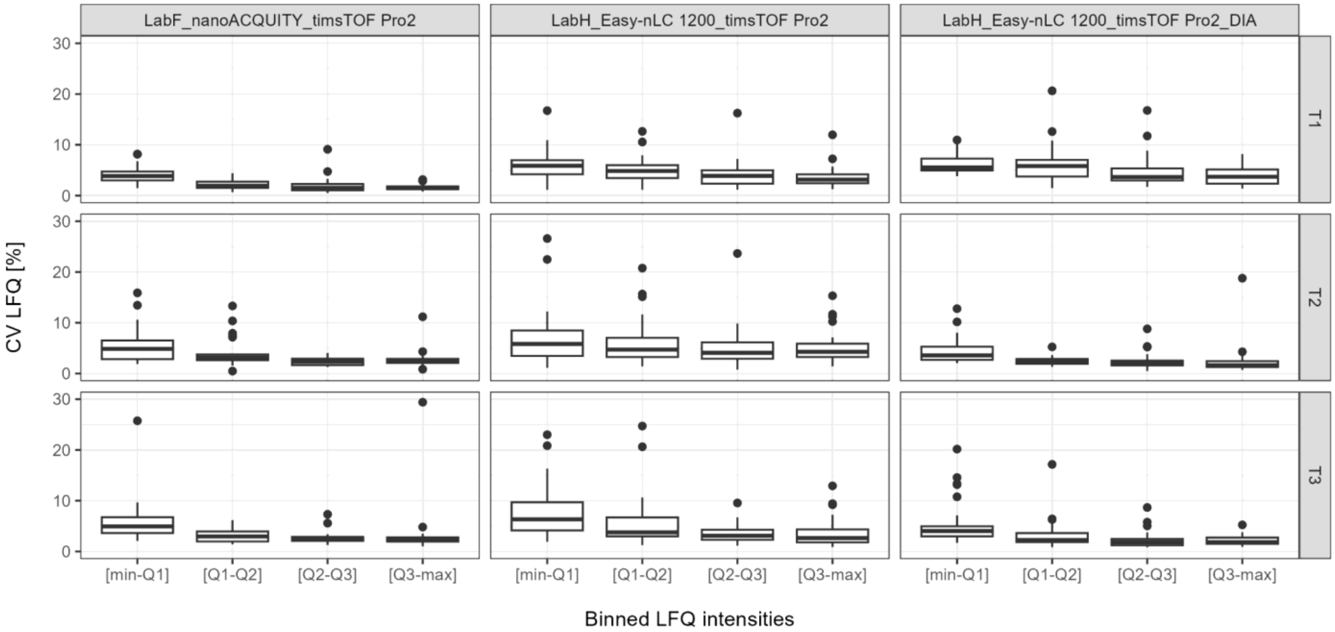

Fig. S38: CV distribution in respect to binned LFQ intensity distribution of all plasma datasets measured on timsTOF Pro2 instruments. Y-axis is cut at 30% to aid data visualization.

6.3 Serum: CV vs. Intensity distribution binned

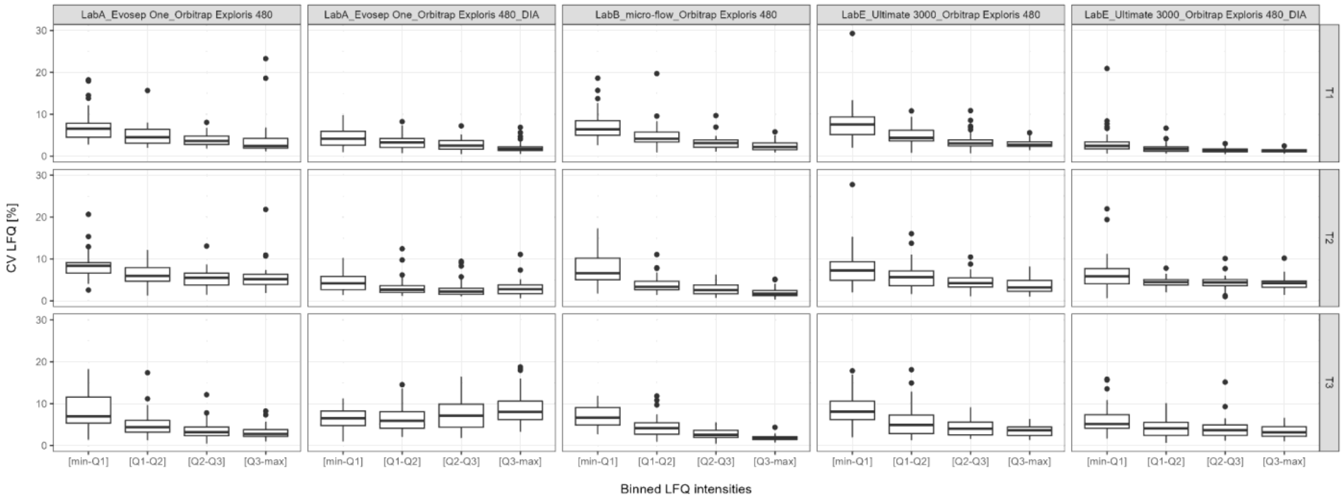

Fig. S39: CV distribution in respect to binned LFQ intensity distribution of all serum datasets measured on Orbitrap Exploris 480 instruments. Y-axis is cut at 30% to aid data visualization.

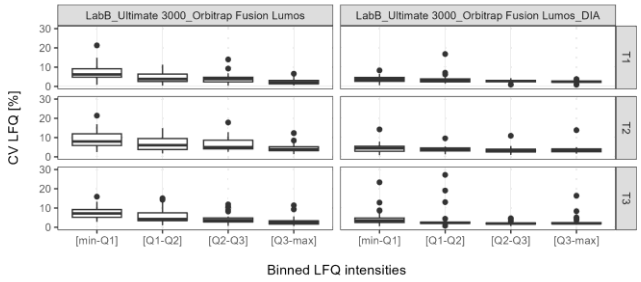

Fig. S40: CV distribution in respect to binned LFQ intensity distribution of all serum datasets measured on Orbitrap Fusion Lumos instruments. Y-axis is cut at 30% to aid data visualization.

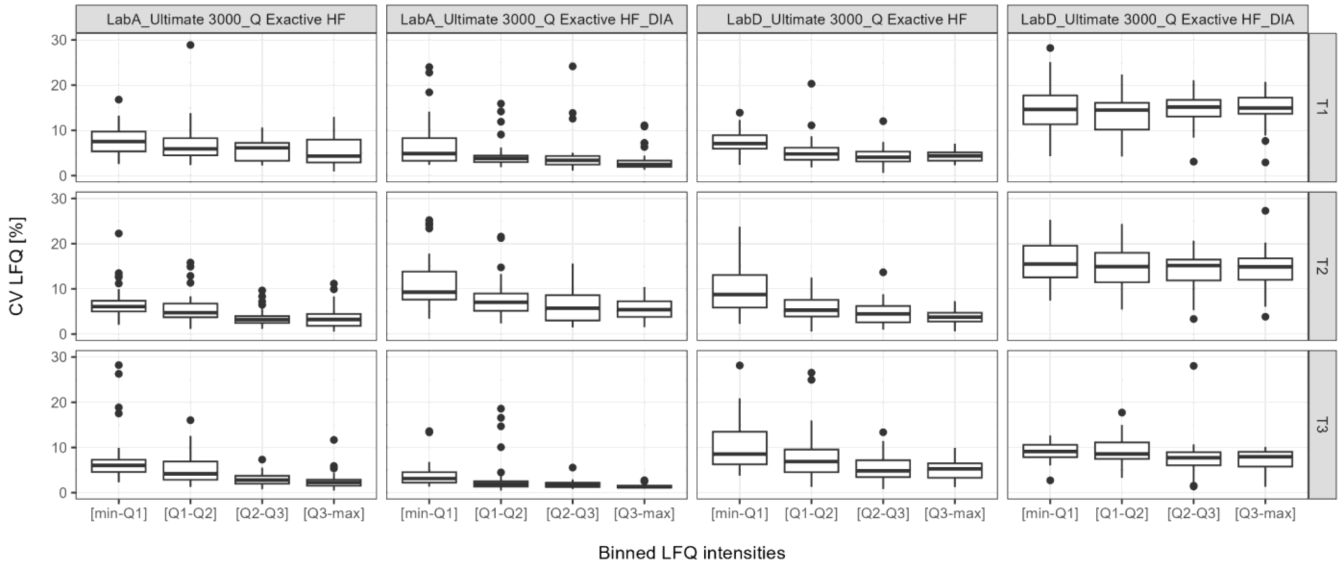

Fig. S41: CV distribution in respect to binned LFQ intensity distribution of all serum datasets measured on Q Exactive HF instruments. Y-axis is cut at 30% to aid data visualization.

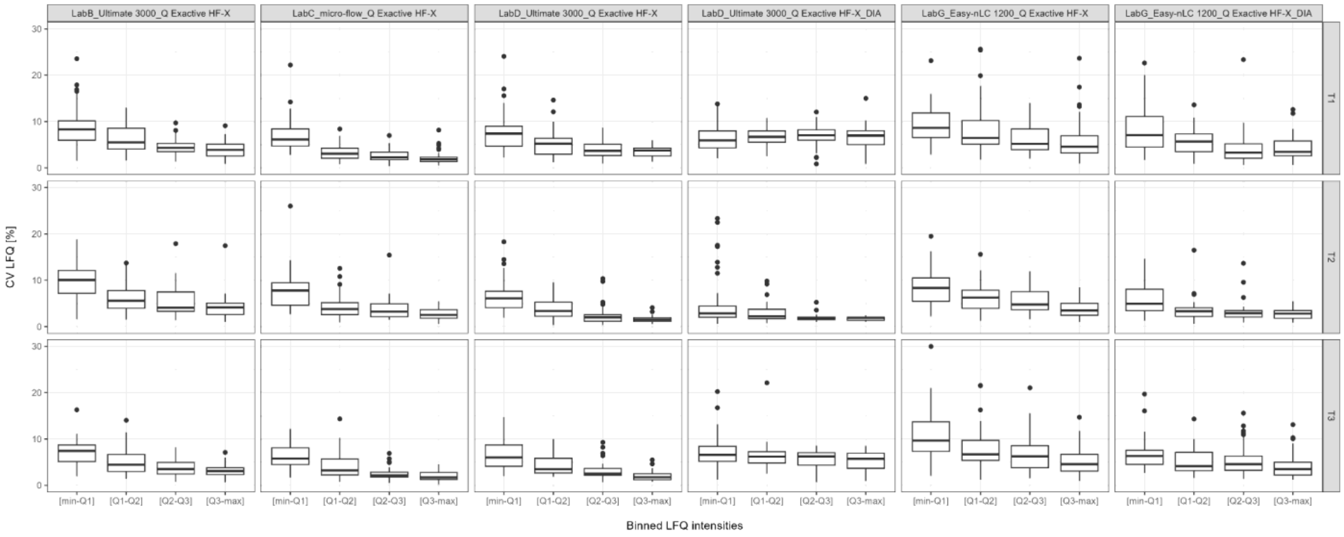

Fig. S42: CV distribution in respect to binned LFQ intensity distribution of all serum datasets measured on Q Exactive HF-X instruments. Y-axis is cut at 30% to aid data visualization.

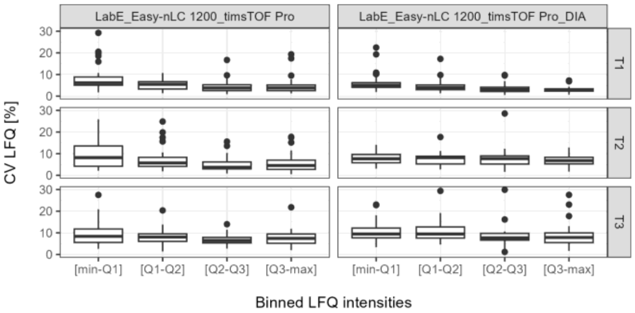

Fig. S43: CV distribution in respect to binned LFQ intensity distribution of all serum datasets measured on timsTOF Pro instruments. Y-axis is cut at 30% to aid data visualization.

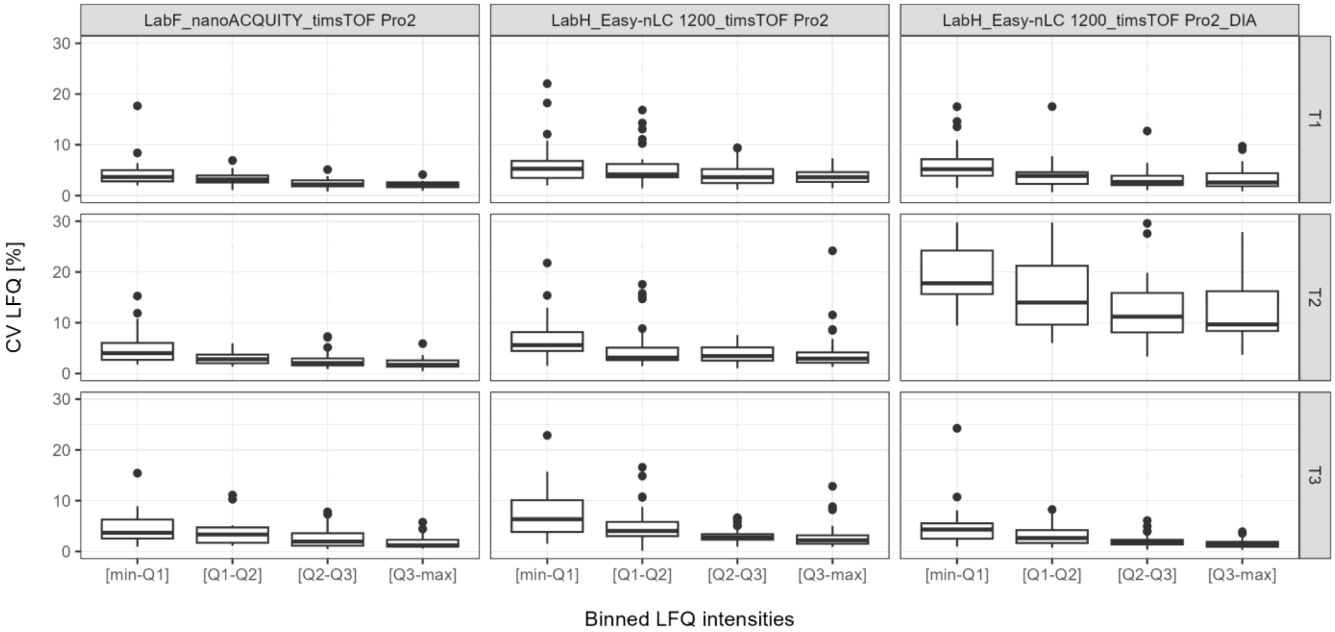

Fig. S44: CV distribution in respect to binned LFQ intensity distribution of all serum datasets measured on timsTOF Pro2 instruments. Y-axis is cut at 30% to aid data visualization.

6.4 Peptides per protein group distribution

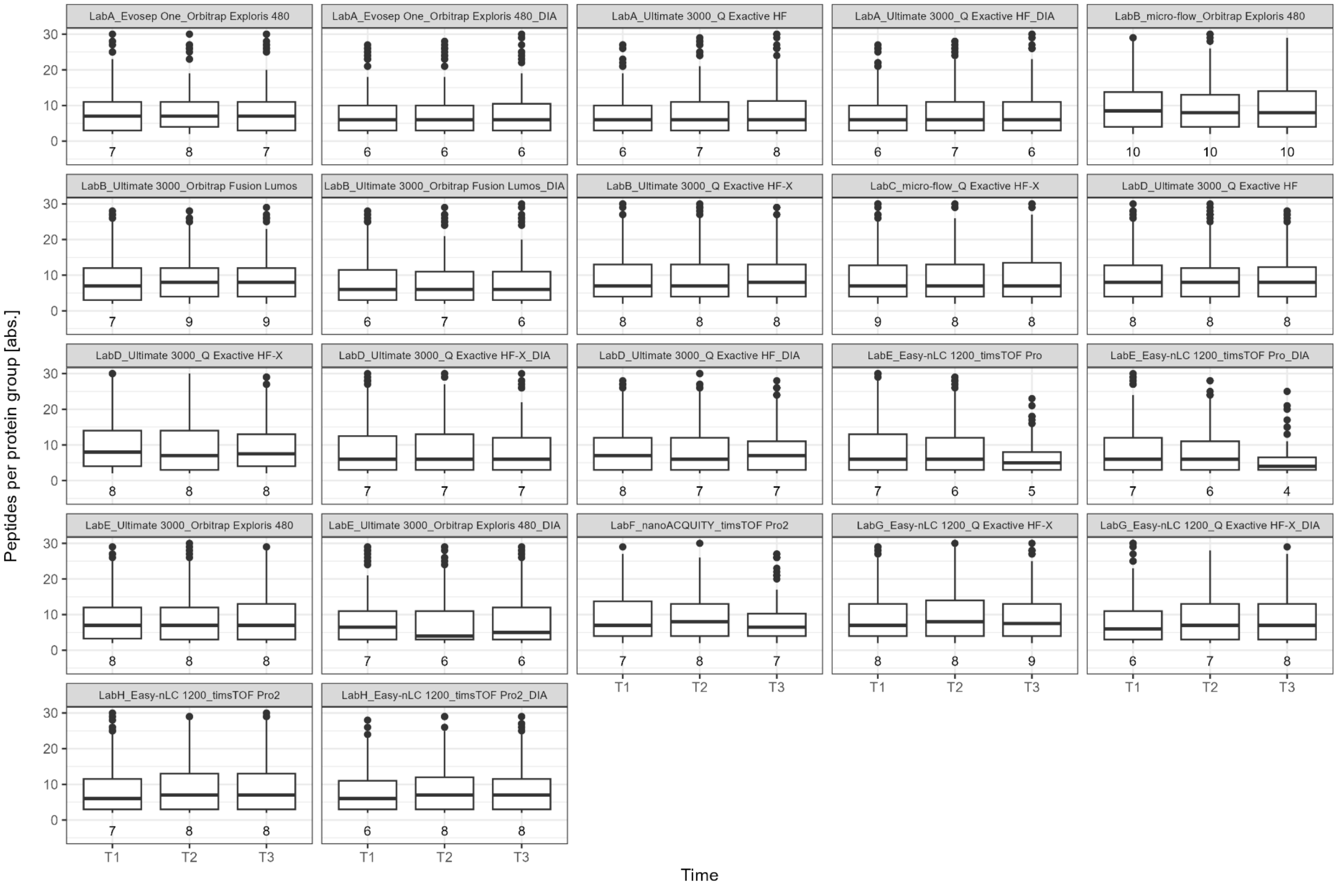

Fig. S45: Distribution of peptides per protein group of all plasma datasets. The median number of peptides per protein group is shown below boxplots. Y-axis is cut at 30 to aid data visualization.

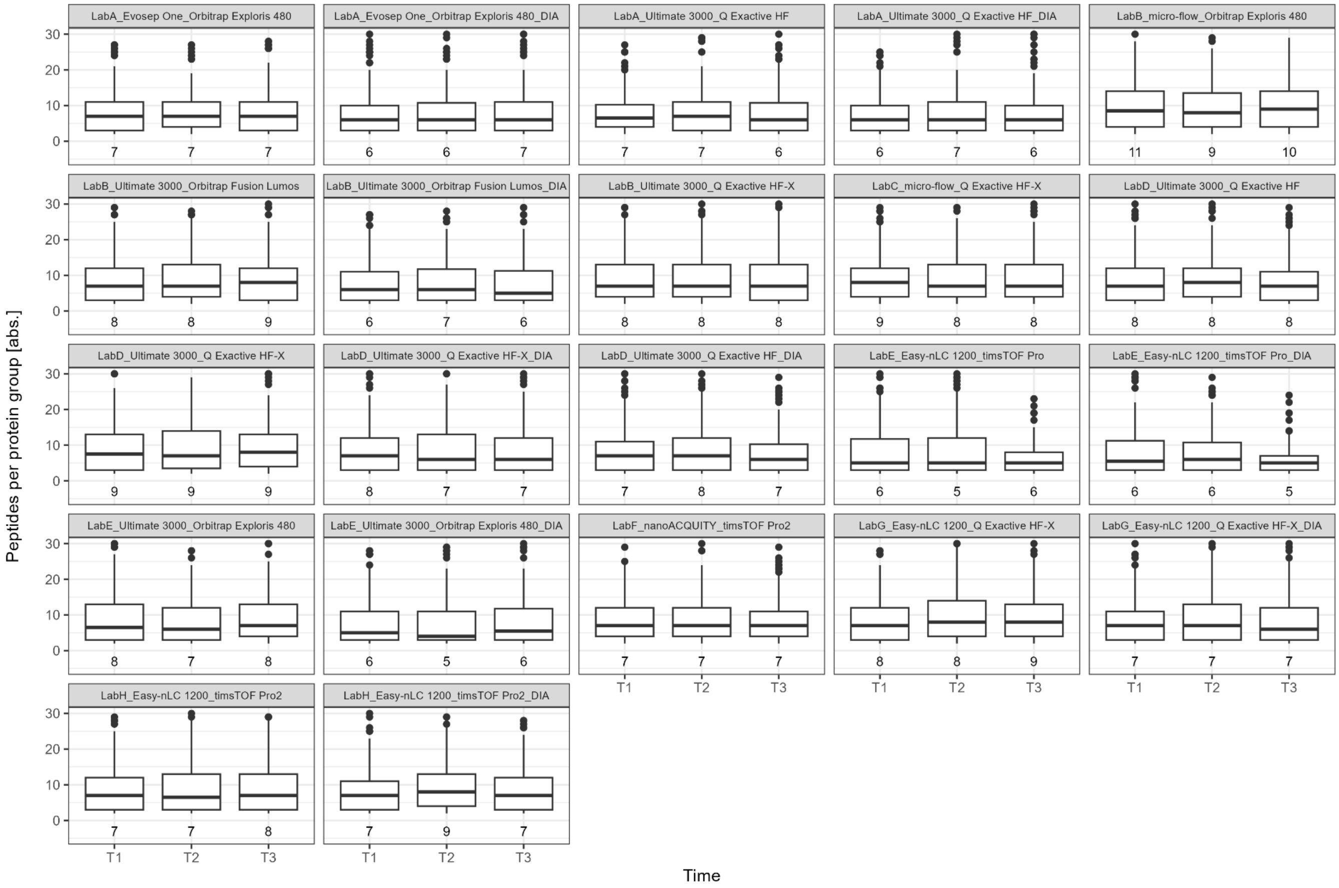

Fig. S46: Distribution of peptides per protein group of all serum datasets. The median number of peptides per protein group is shown below boxplots. Y-axis is cut at 30 to aid data visualization.

6.5 Plasma: CV vs. Binned peptides per protein group distribution

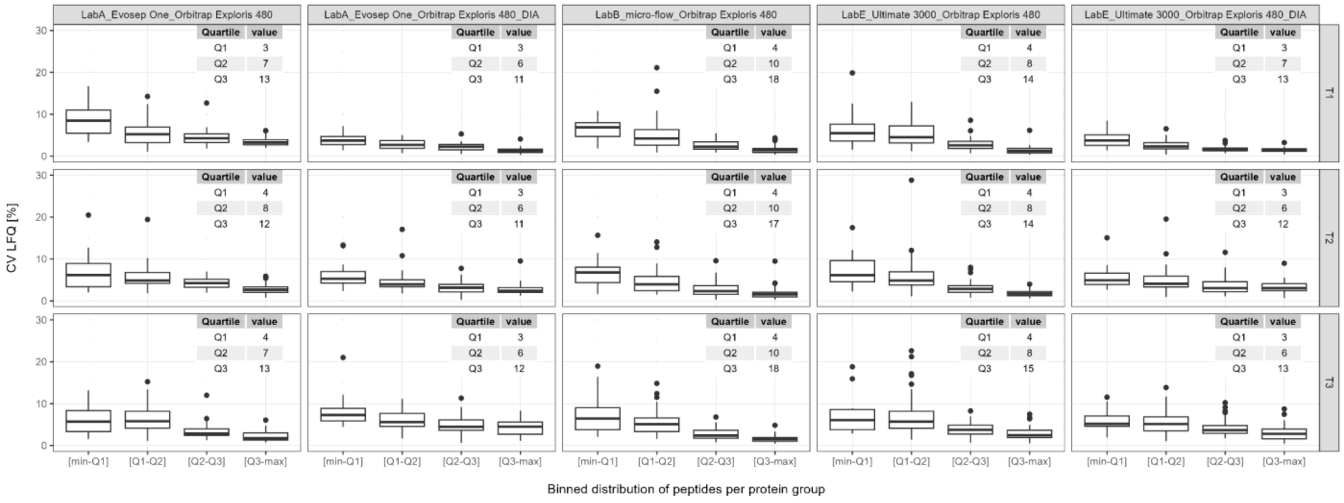

Fig. S47: CV distribution in respect to binned distribution of peptide numbers per protein group of all plasma datasets measured on Orbitrap Exploris 480 instruments. Y-axis is cut at 30% to aid data visualization. Quartile values Q1, Q2 (median) and Q3 for the respective distribution are shown in table.

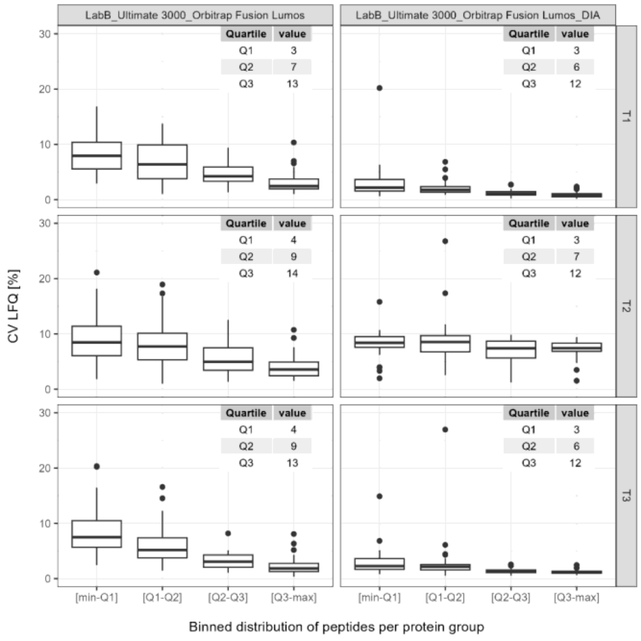

Fig. S48: CV distribution in respect to binned distribution of peptides numbers per protein group of all plasma datasets measured on Orbitrap Fusion Lumos instruments. Y-axis is cut at 30% to aid data visualization. Quartile values Q1, Q2 (median) and Q3 for the respective distribution are shown in table.

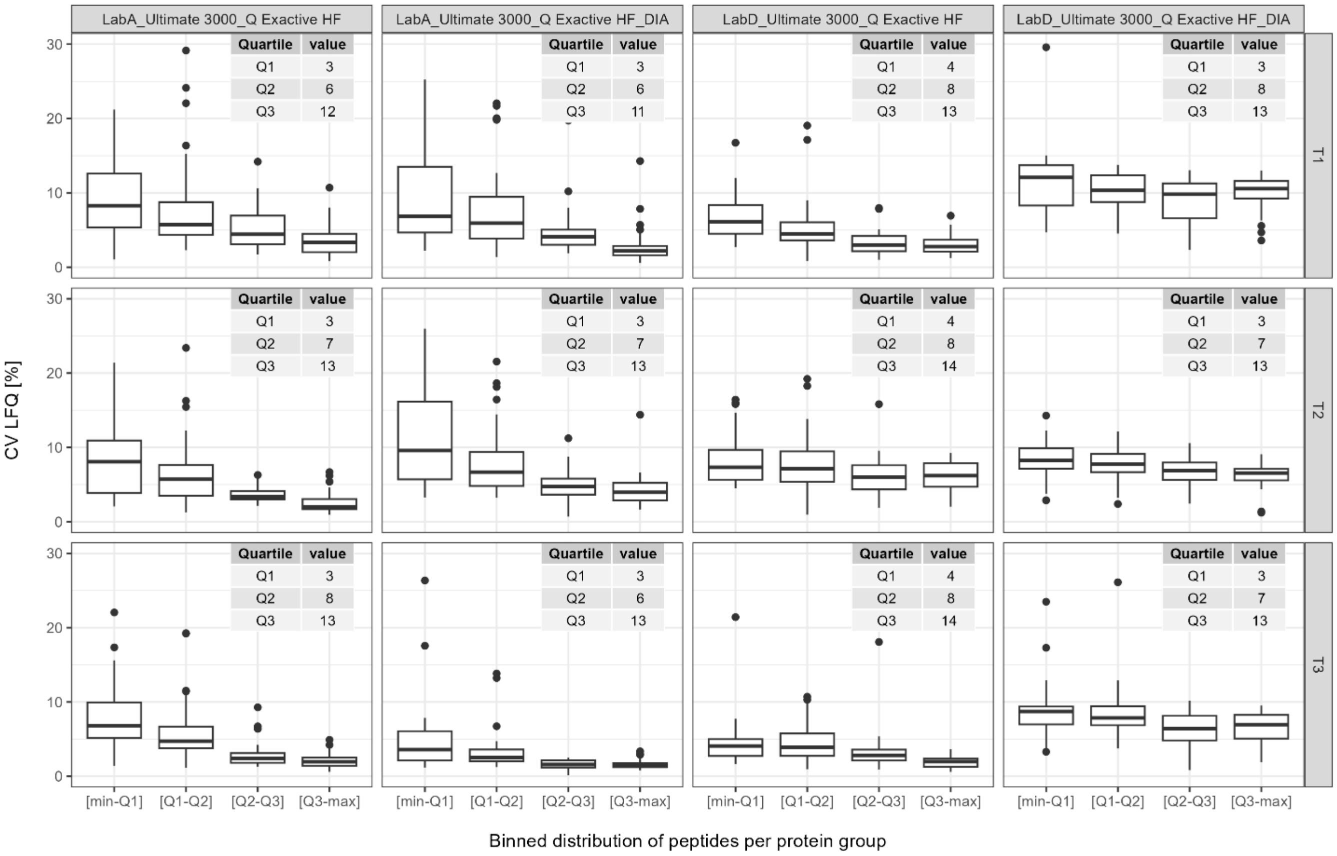

Fig. S49: CV distribution in respect to binned distribution of peptide numbers per protein group of all plasma datasets measured on Q Exactive HF instruments. Y-axis is cut at 30% to aid data visualization. Quartile values Q1, Q2 (median) and Q3 for the respective distribution are shown in table.

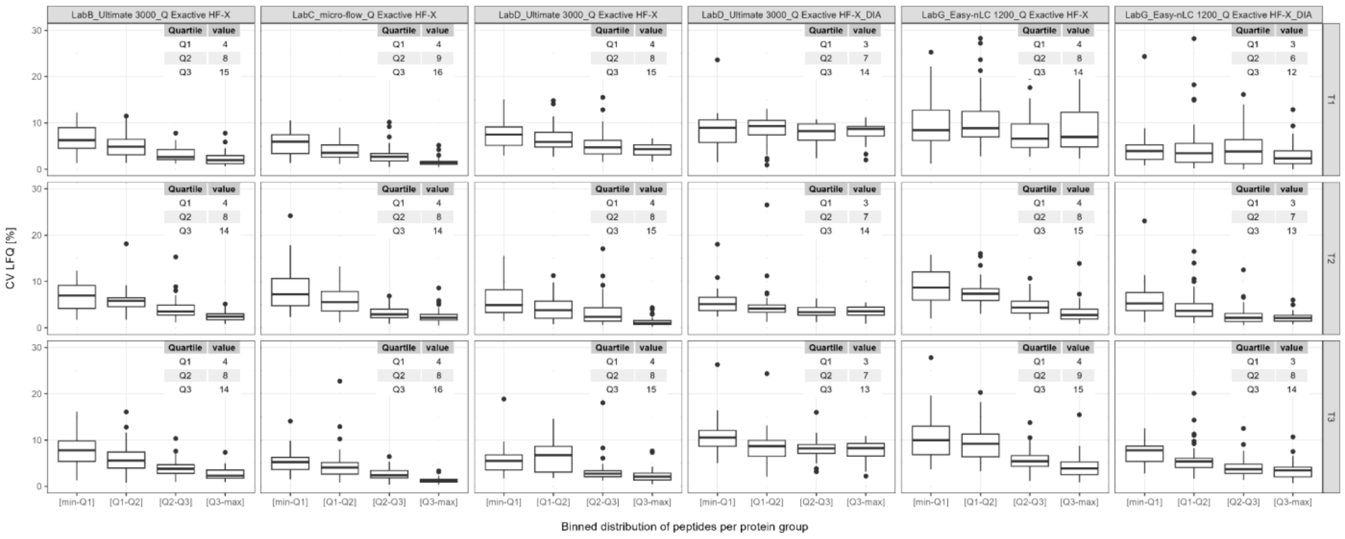

Fig. S50: CV distribution in respect to binned distribution of peptide numbers per protein group of all plasma datasets measured on Q Exactive HF-X instruments. Y-axis is cut at 30% to aid data visualization. Quartile values Q1, Q2 (median) and Q3 for the respective distribution are shown in table.

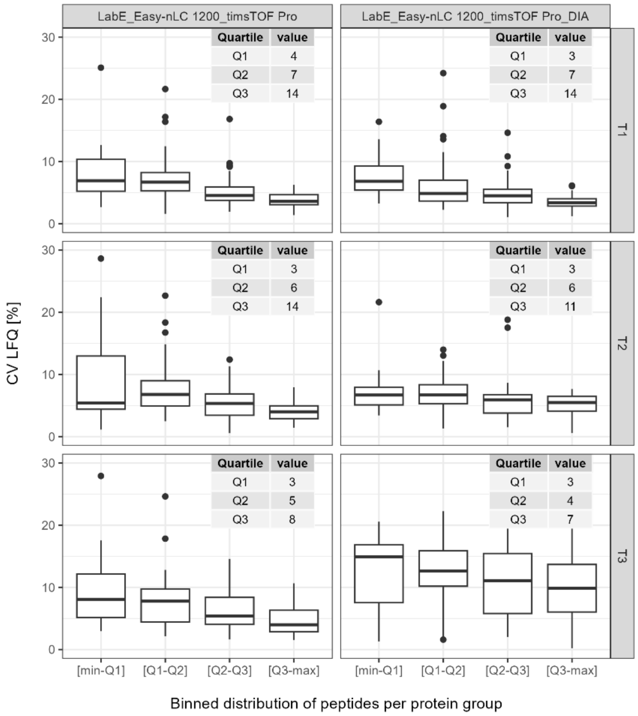

Fig. S51: CV distribution in respect to binned distribution of peptide numbers per protein group of all plasma datasets measured on timsTOF Pro instruments. Y-axis is cut at 30% to aid data visualization. Quartile values Q1, Q2 (median) and Q3 for the respective distribution are shown in table.

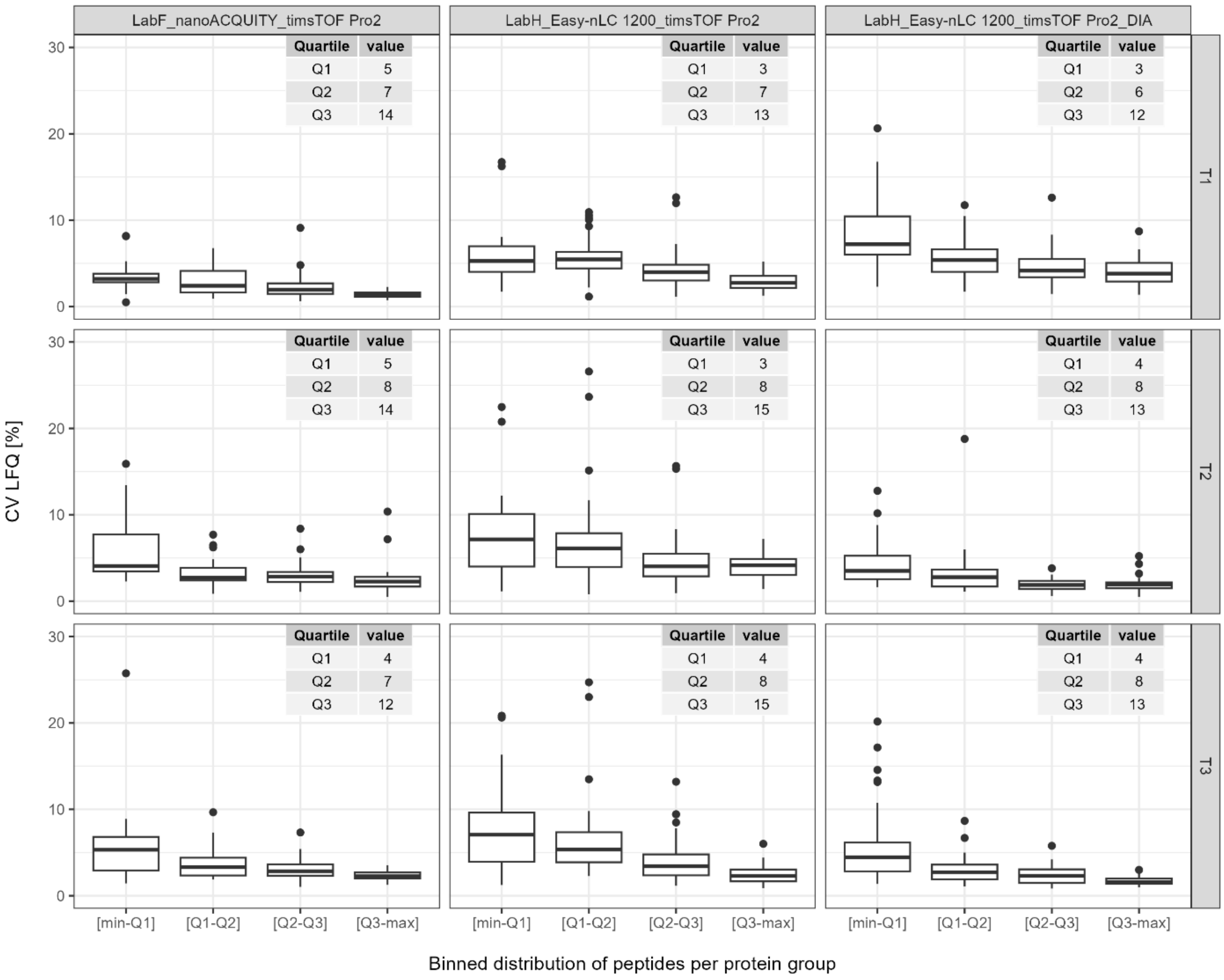

Fig. S52: CV distribution in respect to binned distribution of peptide numbers per protein group of all plasma datasets measured on timsTOF Pro2 instruments. Y-axis is cut at 30% to aid data visualization. Quartile values Q1, Q2 (median) and Q3 for the respective distribution are shown in table.

6.6 Serum: CV vs. Binned peptides per protein group distribution

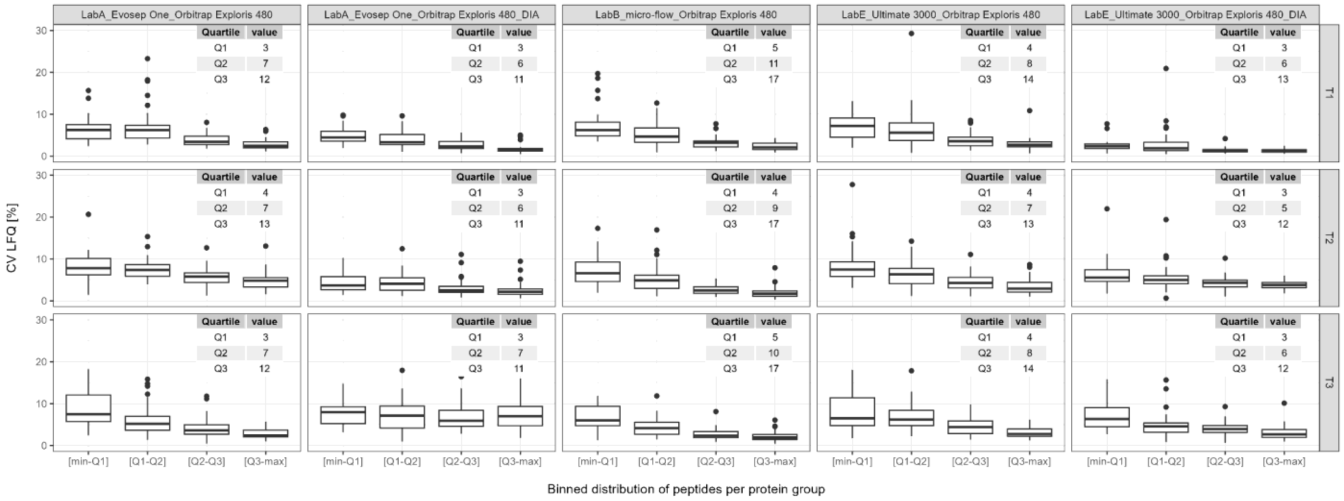

Fig. S53: CV distribution in respect to binned distribution of peptide numbers per protein group of all serum datasets measured on Orbitrap Exploris 480 instruments. Y-axis is cut at 30% to aid data visualization. Quartile values Q1, Q2 (median) and Q3 for the respective distribution are shown in table.

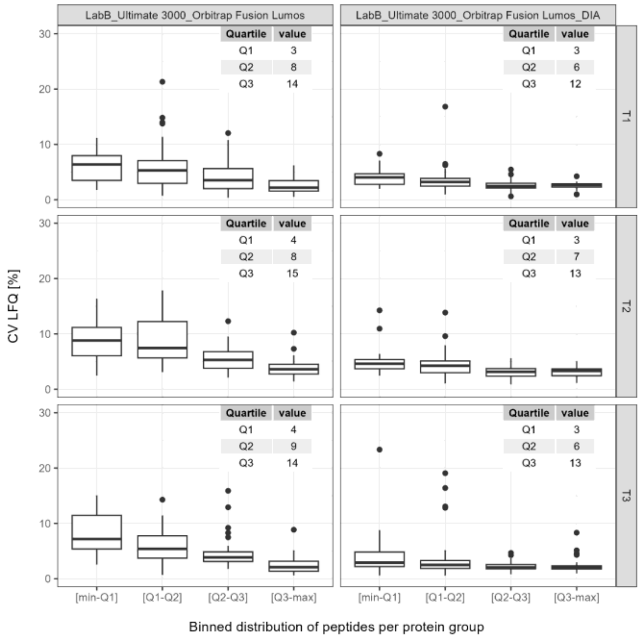

Fig. S54: CV distribution in respect to binned distribution of peptide numbers per protein group of all serum datasets measured on Orbitrap Fusion Lumos instruments. Y-axis is cut at 30% to aid data visualization. Quartile values Q1, Q2 (median) and Q3 for the respective distribution are shown in table.

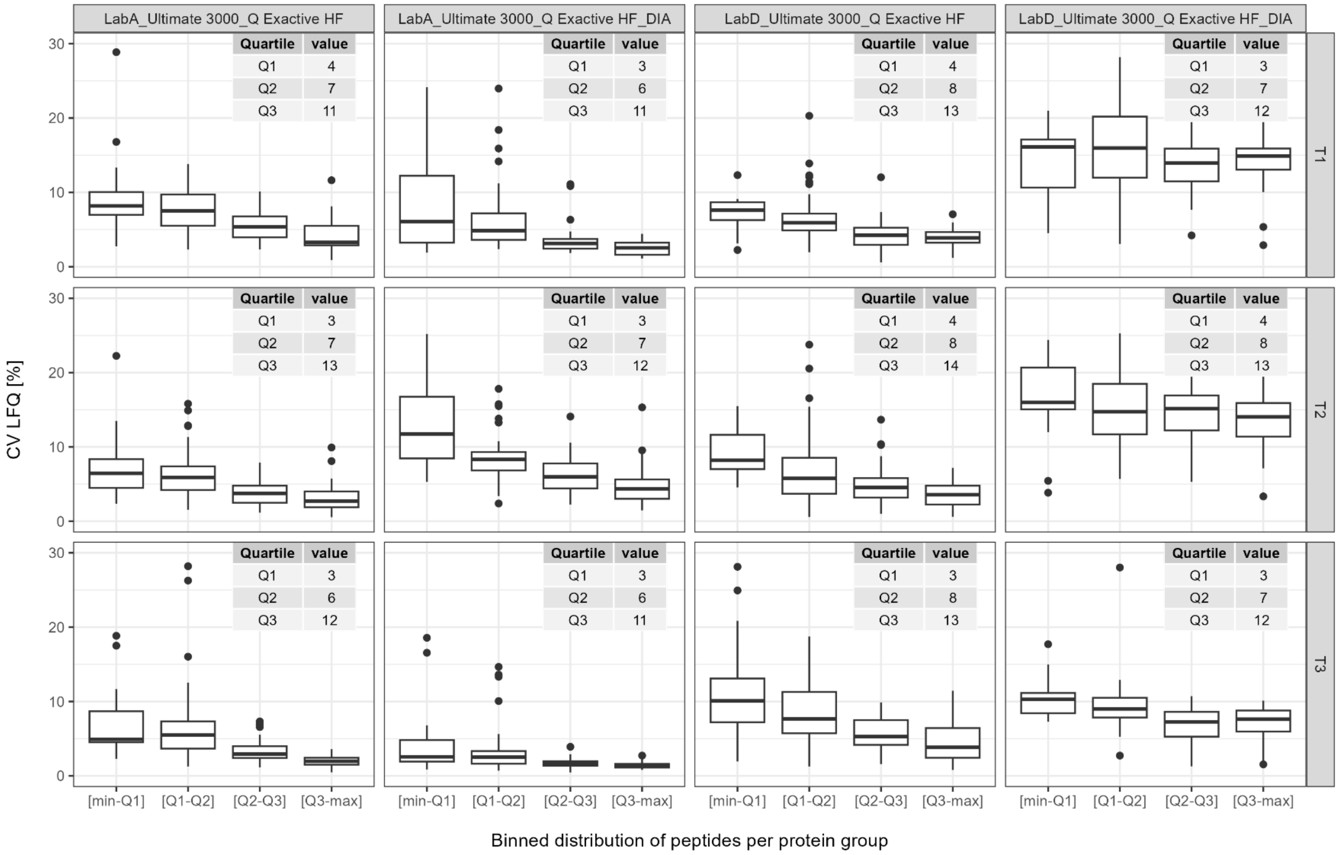

Fig. S55: CV distribution in respect to binned distribution of peptide numbers per protein group of all serum datasets measured on Q Exactive HF instruments. Y-axis is cut at 30% to aid data visualization. Quartile values Q1, Q2 (median) and Q3 for the respective distribution are shown in table.

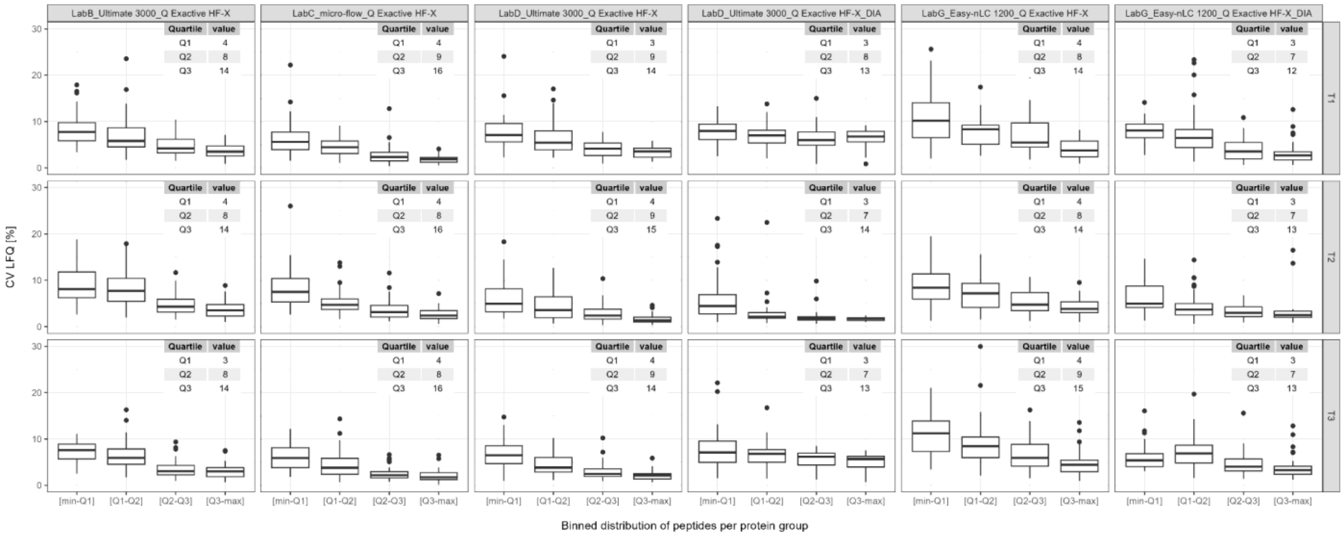

Fig. S56: CV distribution in respect to binned distribution of peptide numbers per protein group of all serum datasets measured on Q Exactive HF-X instruments. Y-axis is cut at 30% to aid data visualization. Quartile values Q1, Q2 (median) and Q3 for the respective distribution are shown in table.

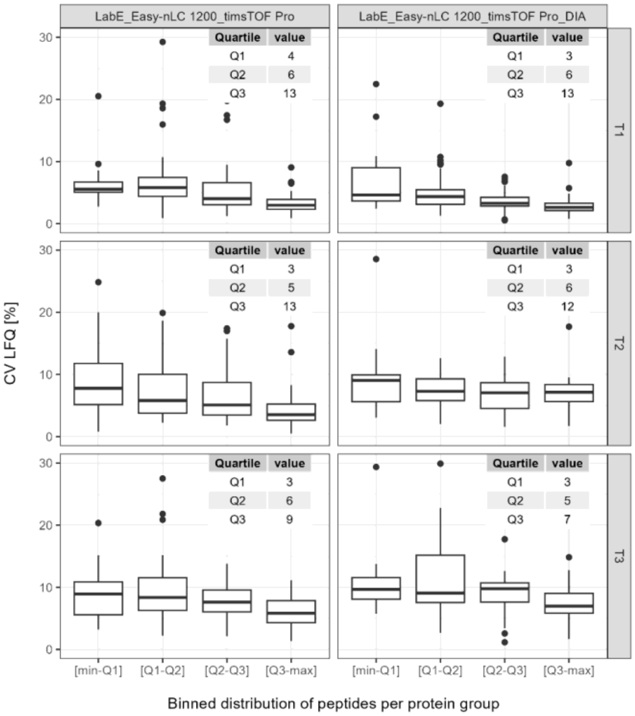

Fig. S57: CV distribution in respect to binned distribution of peptide numbers per protein group of all serum datasets measured on timsTOF Pro instruments. Y-axis is cut at 30% to aid data visualization. Quartile values Q1, Q2 (median) and Q3 for the respective distribution are shown in table.

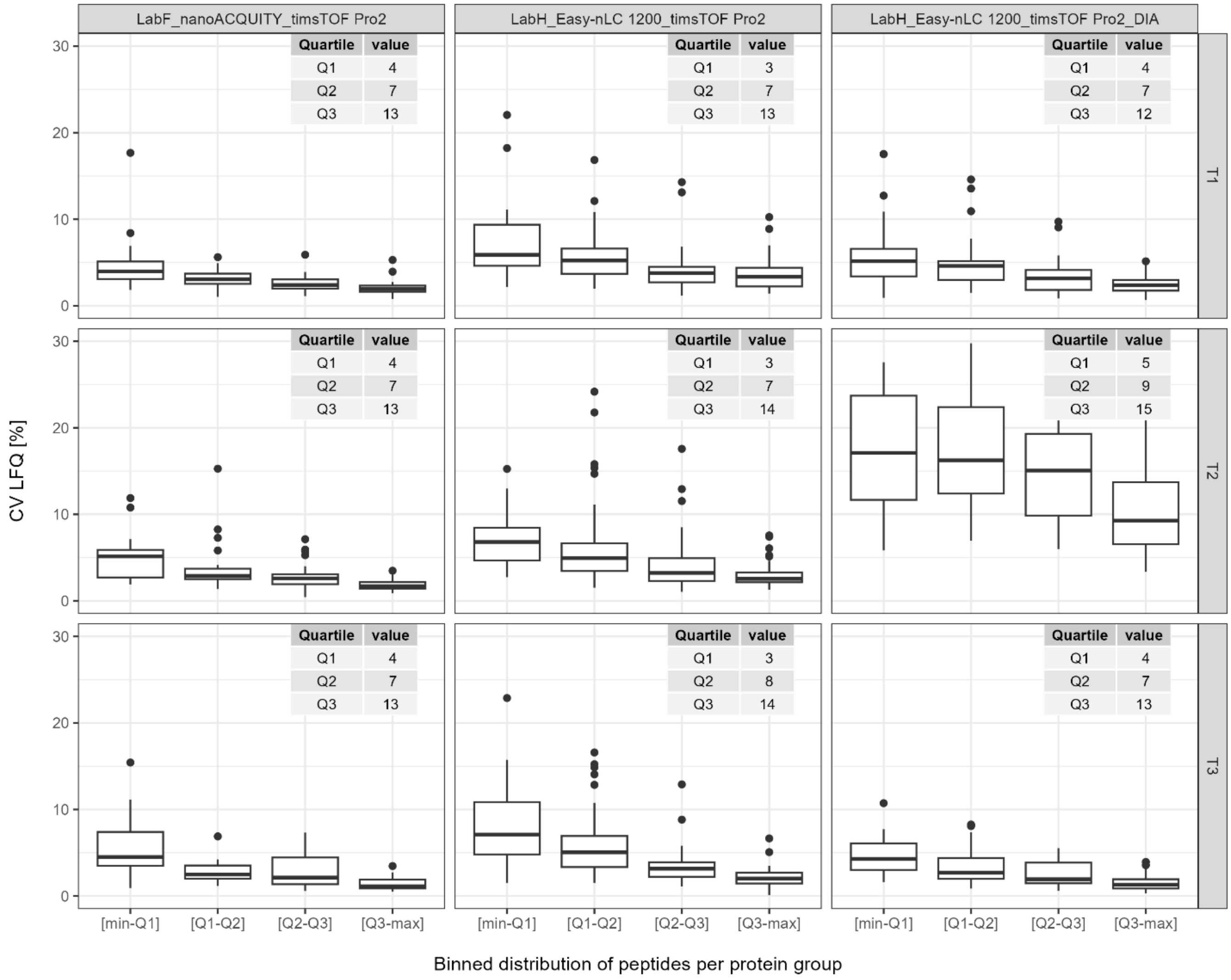

Fig. S58: CV distribution in respect to binned distribution of peptide numbers per protein group of all serum datasets measured on timsTOF Pro2 instruments. Y-axis is cut at 30% to aid data visualization. Quartile values Q1, Q2 (median) and Q3 for the respective distribution are shown in table.

6.7 Protein group-level: Mean and Standard Deviation

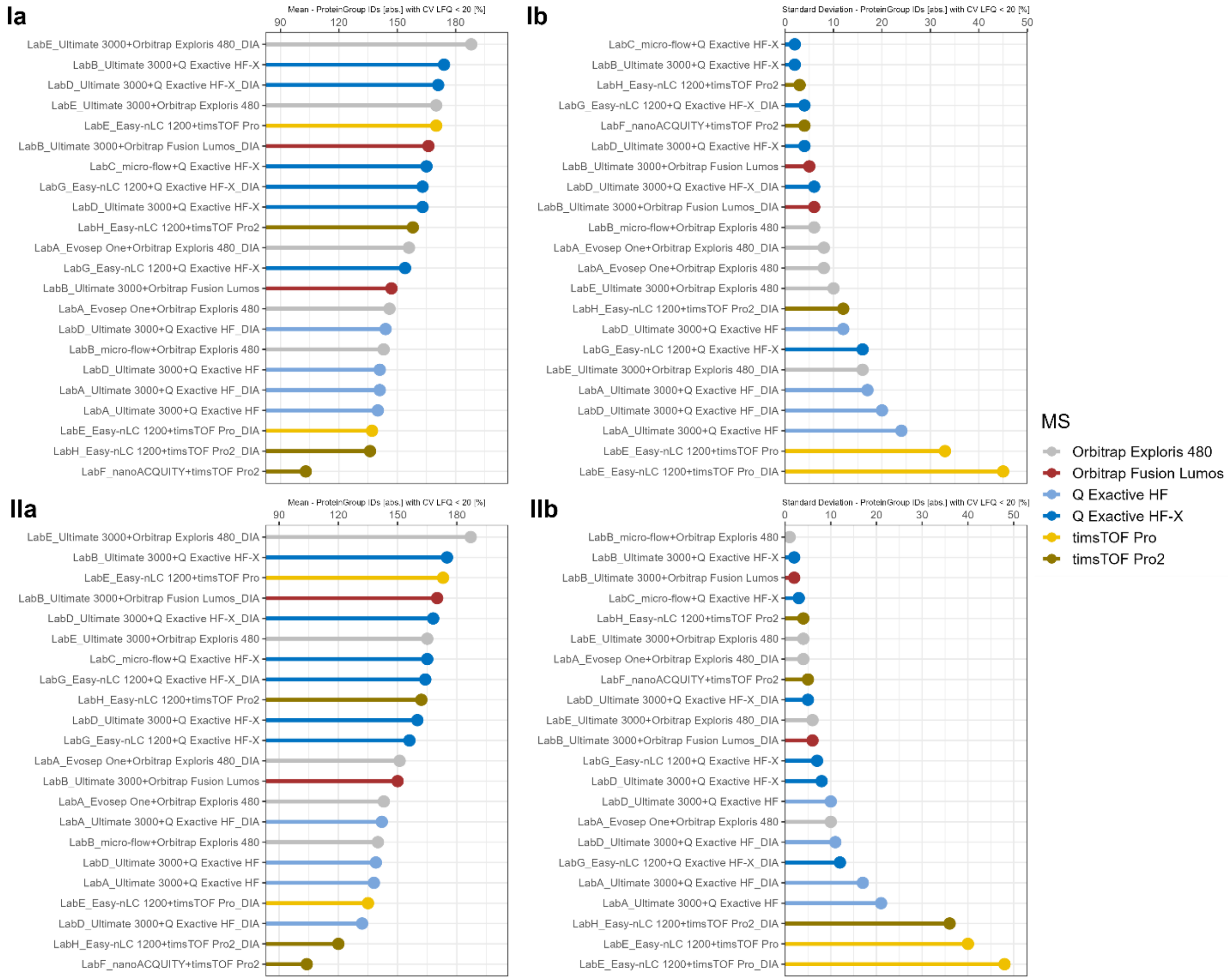

Fig. S59: Numbers of protein group IDs [abs.] with CV LFQ < 20% - achieved mean number of identifications in decreasing order (a) and standard deviation in increasing order (b) on protein group-level for plasma (I) and serum (II). Results are color coded by MS instrument.

7. Inter-laboratory reproducibility

7.1 Protein-level: Absolute overlap of IDs

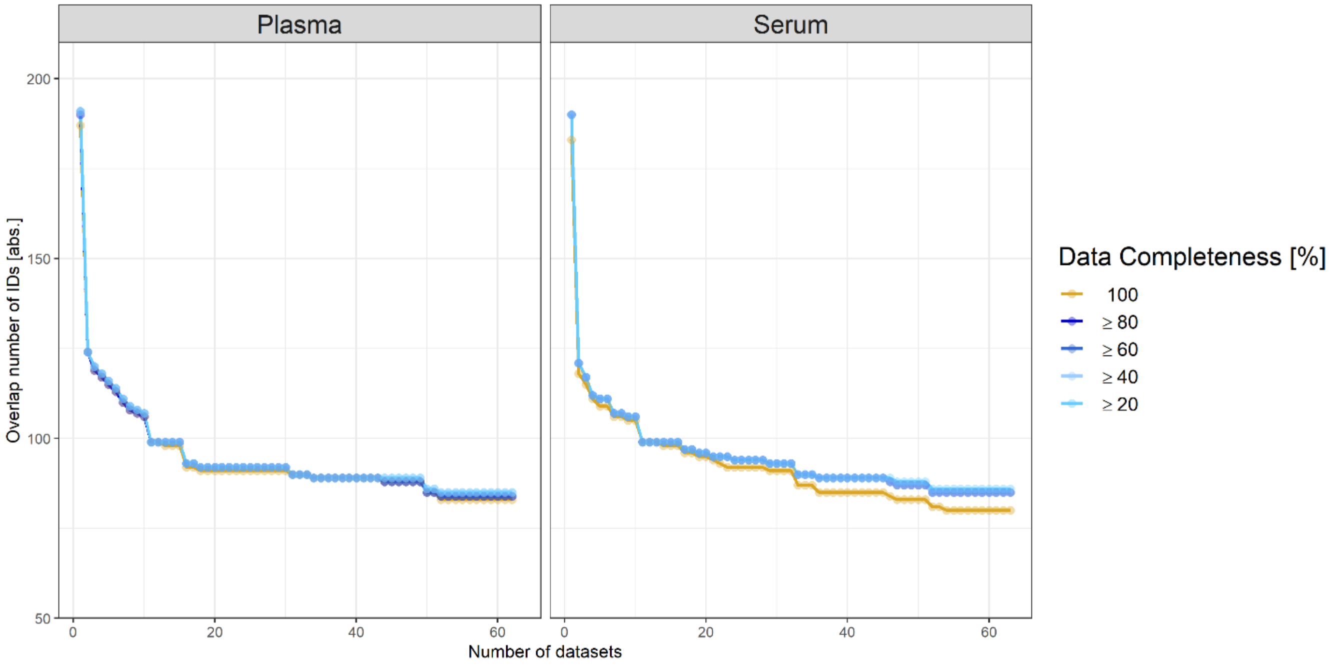

Fig. S60: Overlapping protein IDs [abs.] for plasma and serum with different levels of data completeness [%] depending on the number of datasets.

7.2 Protein-level: Total number of IDs & relative overlap of IDs

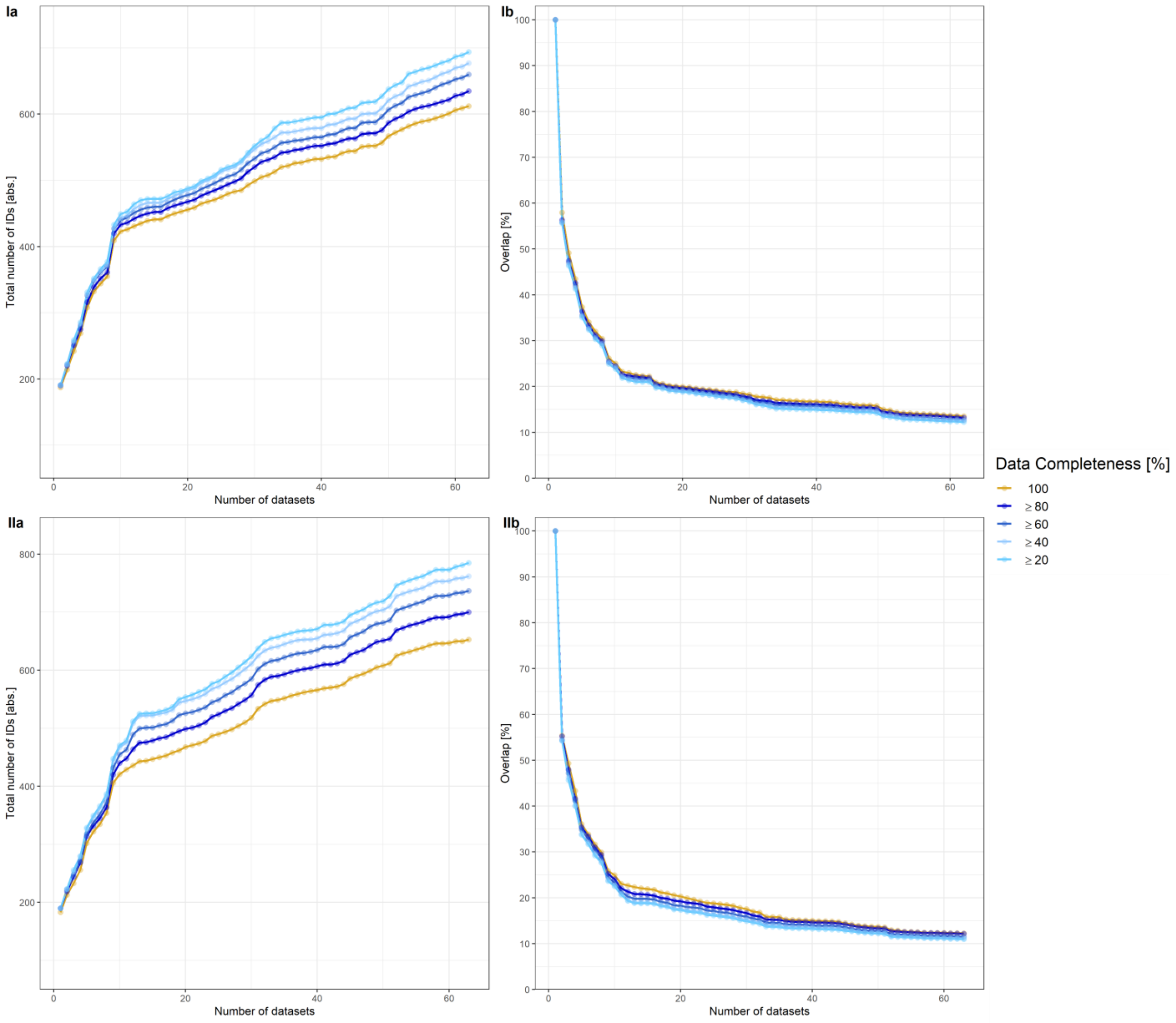

Fig. S61: Total number of protein IDs [abs.] (a) and relative overlap of IDs [%] (b) for plasma (I) and serum (II) for different levels of data completeness [%] depending on the number of datasets.

7.3 Peptide-level: Absolute overlap of IDs

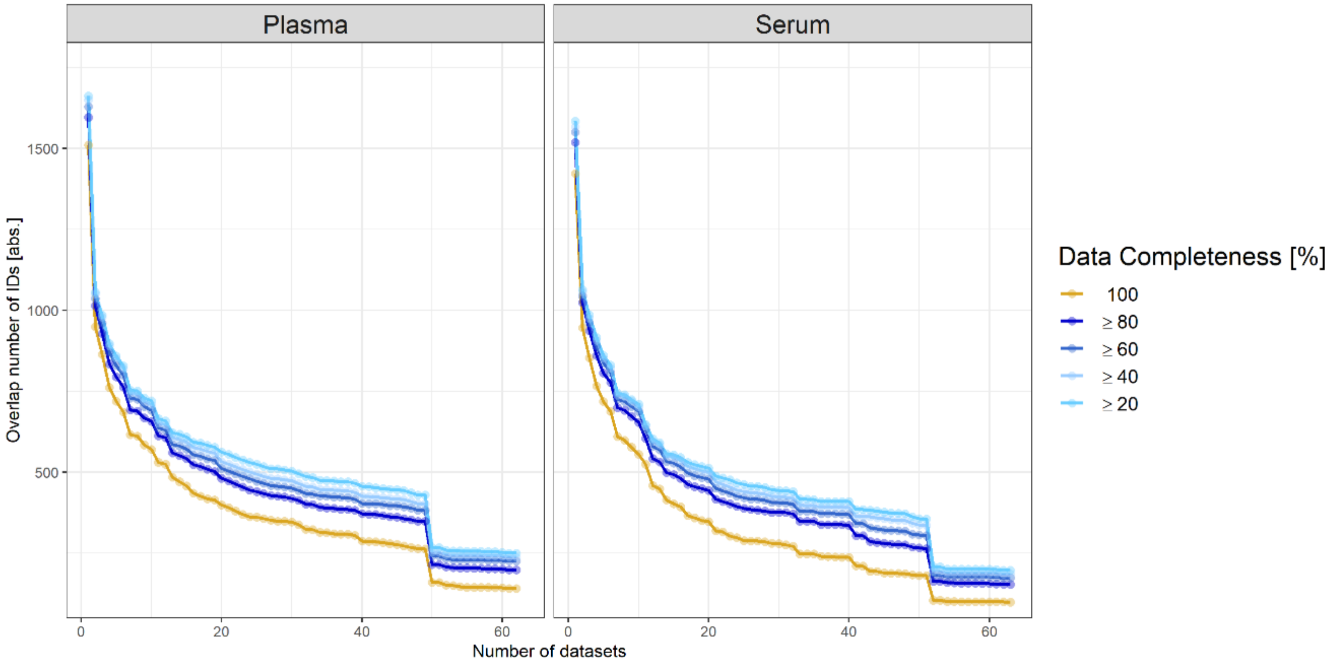

Fig. S62: Overlapping peptide IDs [abs.] for plasma and serum with different levels of data completeness [%] depending on the number of datasets.

7.4 Peptide-level: Total number of IDs & relative overlap of IDs

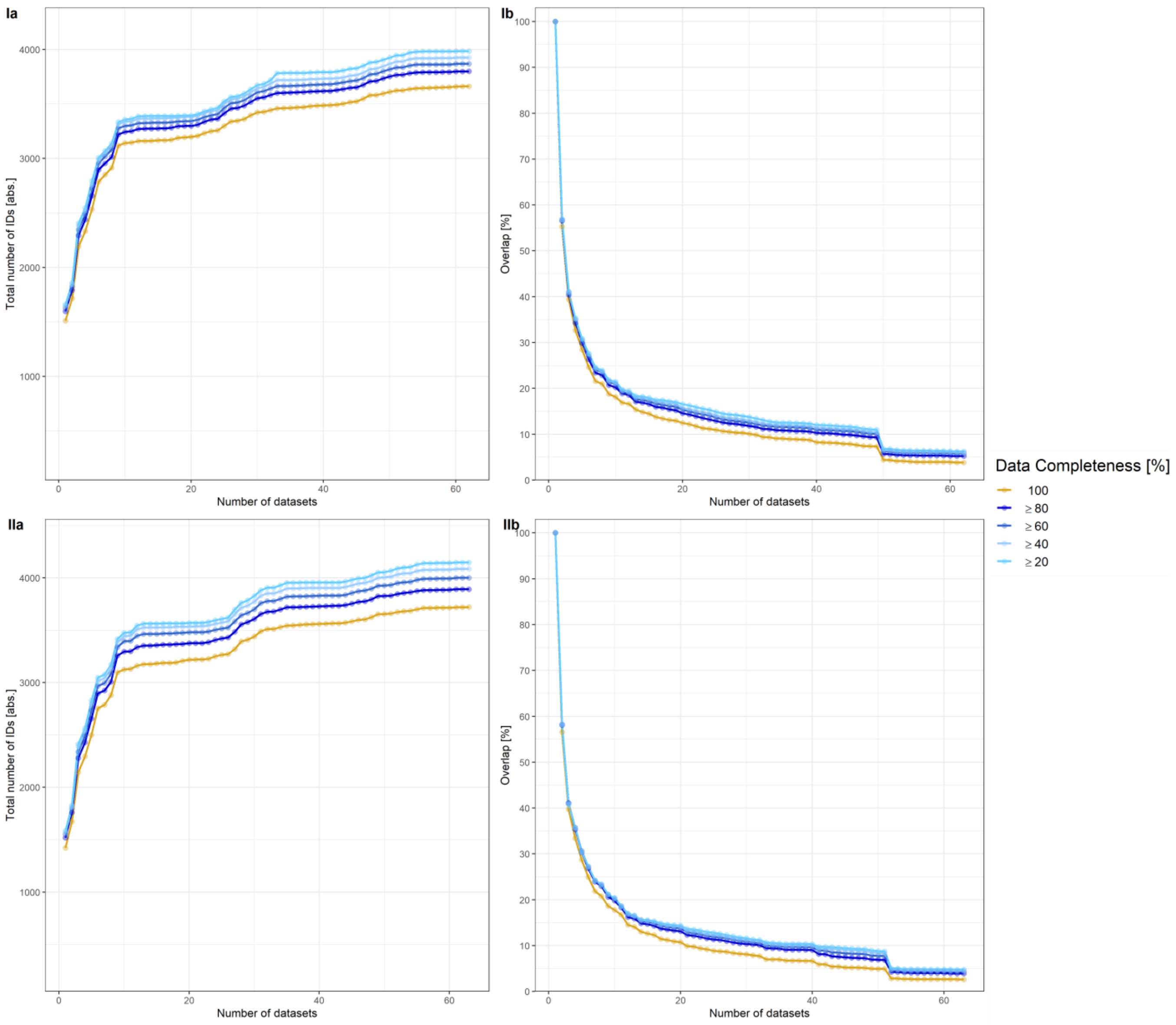

Fig. S63. Total number of peptide IDs [abs.] (a) and relative overlap of IDs [%] (b) for plasma (I) and serum (II) for different levels of data completeness [%] depending on the number of datasets.

7.5 Upset plot

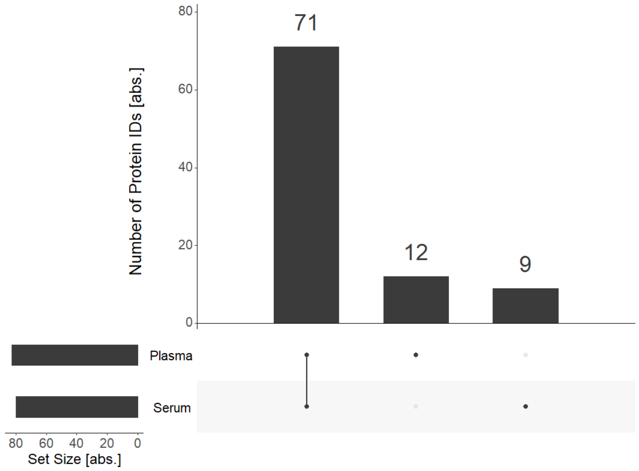

Fig. S64: Upset plot for comparing the identified overlapping protein IDs [abs.], which are present in each technical replicate (100% DC) and in all datasets, for plasma and serum, respectively.

7.6 Protein-level: Intensity distributions

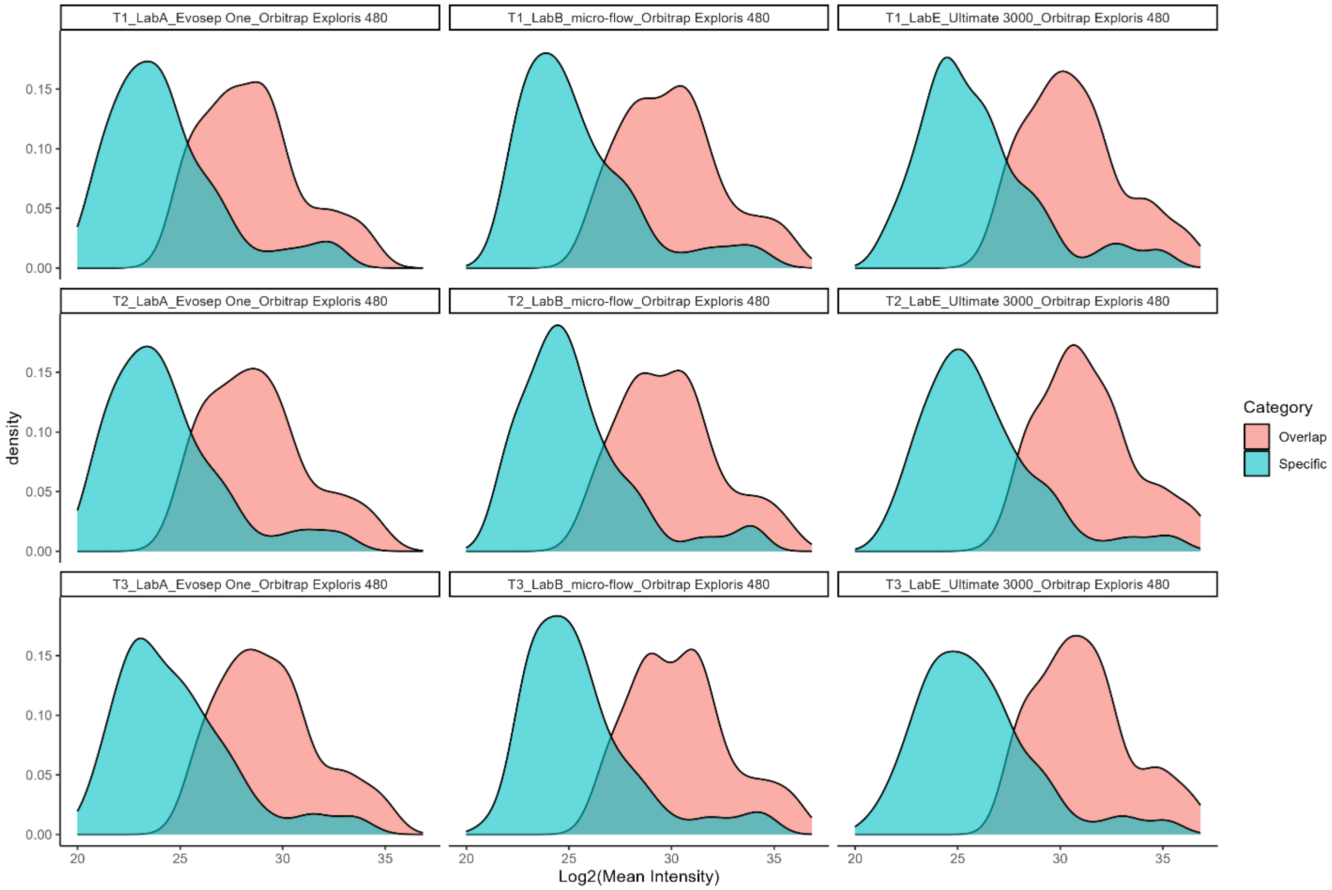

Fig. S65. Plasma sample intensity distributions for datasets measured on Orbitrap Exploris 480 instruments. Highlighted in red are overlapping proteins and in blue dataset specific proteins.

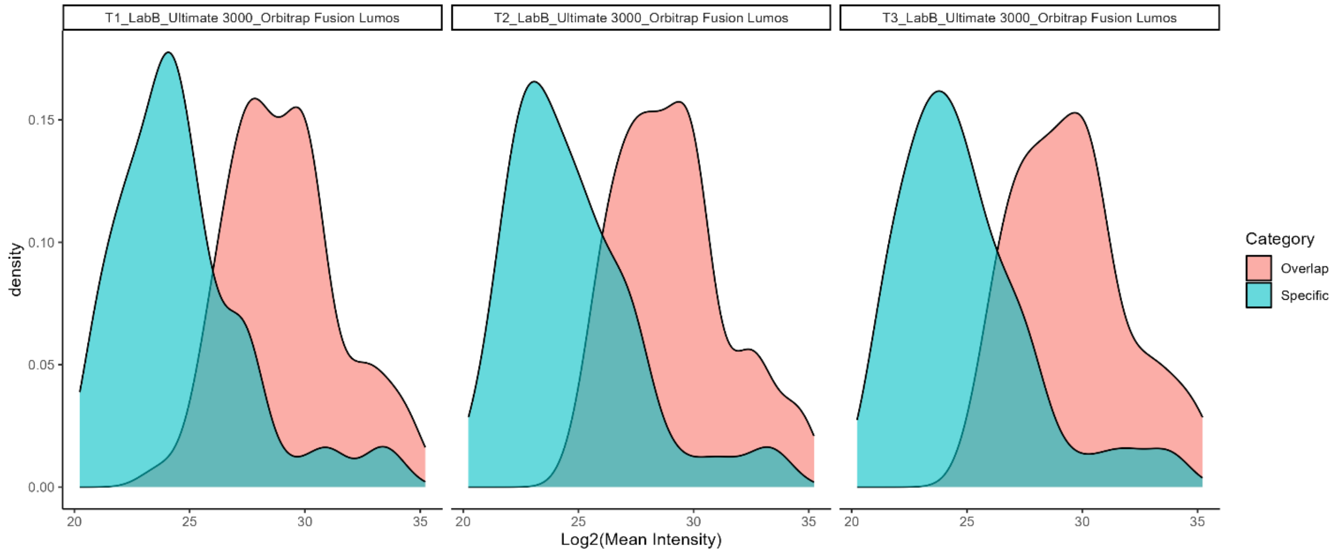

Fig. S66. Plasma sample intensity distributions for datasets measured on Orbitrap Fusion Lumos instruments. Highlighted in red are overlapping proteins and in blue dataset specific proteins.

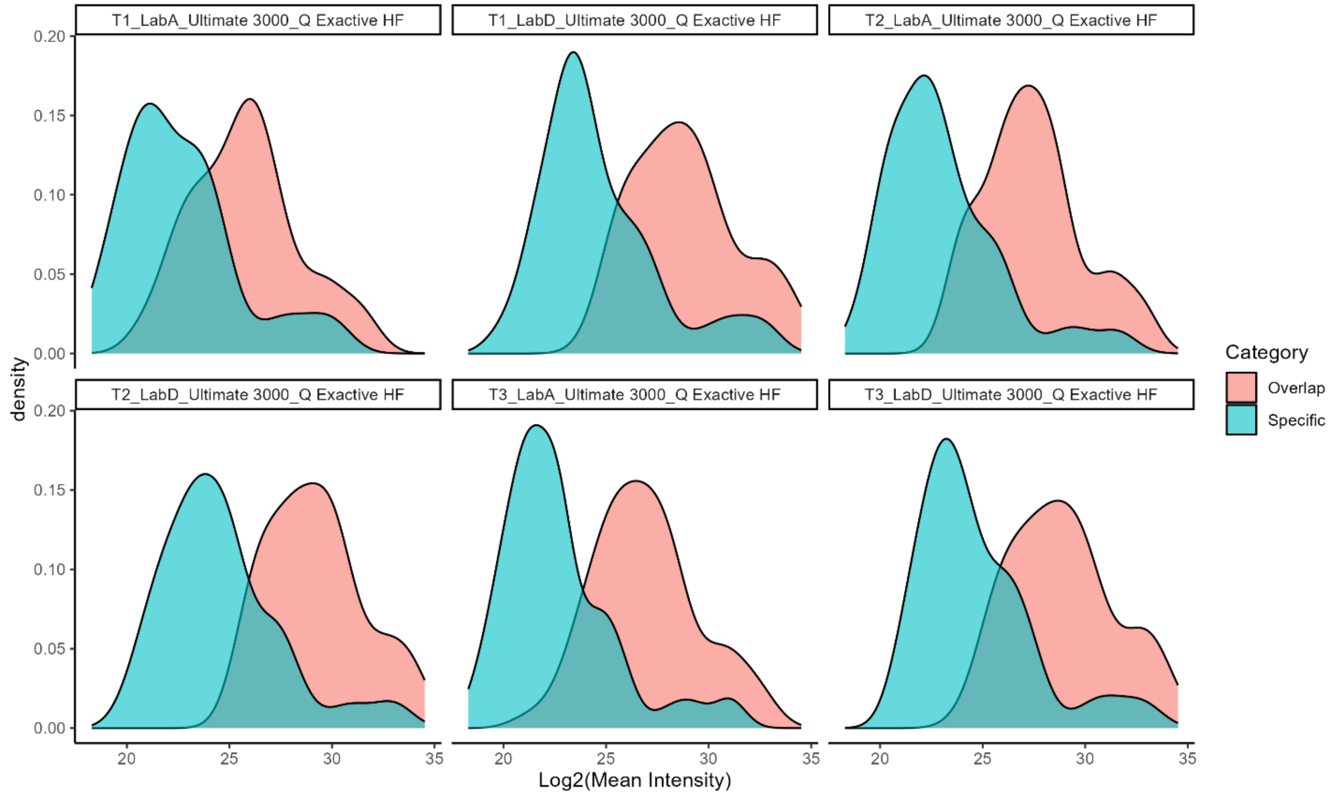

Fig. S67. Plasma sample intensity distributions for datasets measured on Q Exactive HF instruments. Highlighted in red are overlapping proteins and in blue dataset specific proteins.

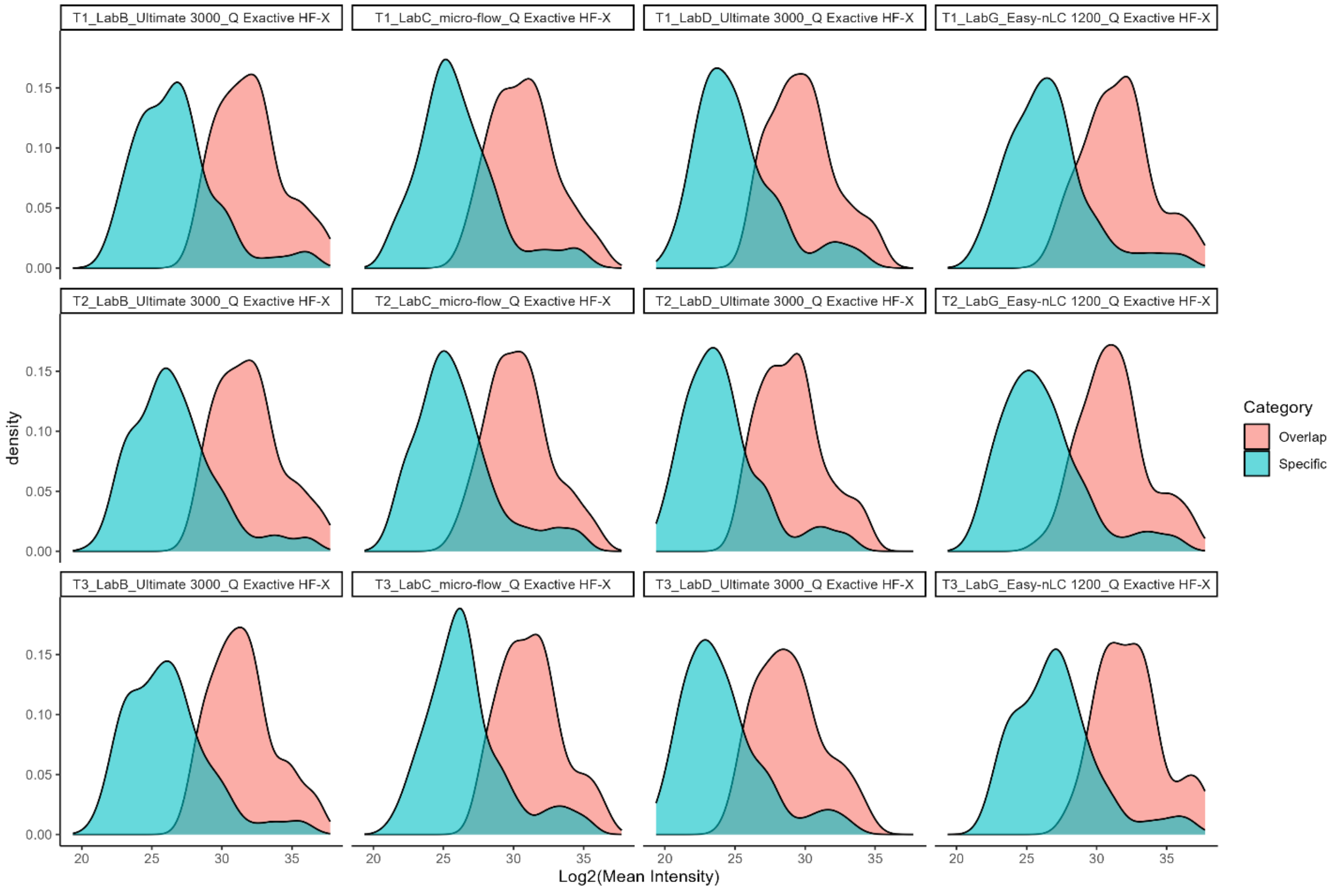

Fig. S68. Plasma sample intensity distributions for datasets measured on Q Exactive HF-X instruments. Highlighted in red are overlapping proteins and in blue dataset specific proteins.

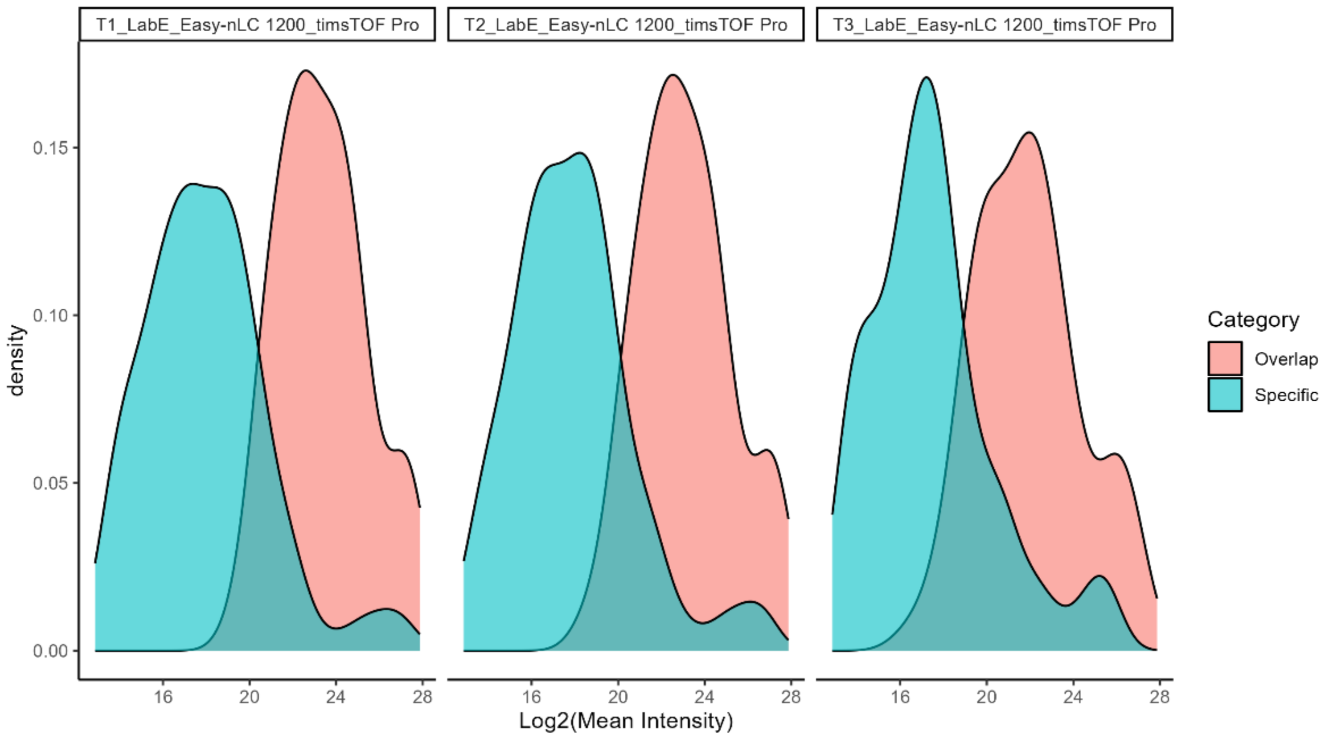

Fig. S69. Plasma sample intensity distributions for datasets measured on timsTOF Pro instruments. Highlighted in red are overlapping proteins and in blue dataset specific proteins.

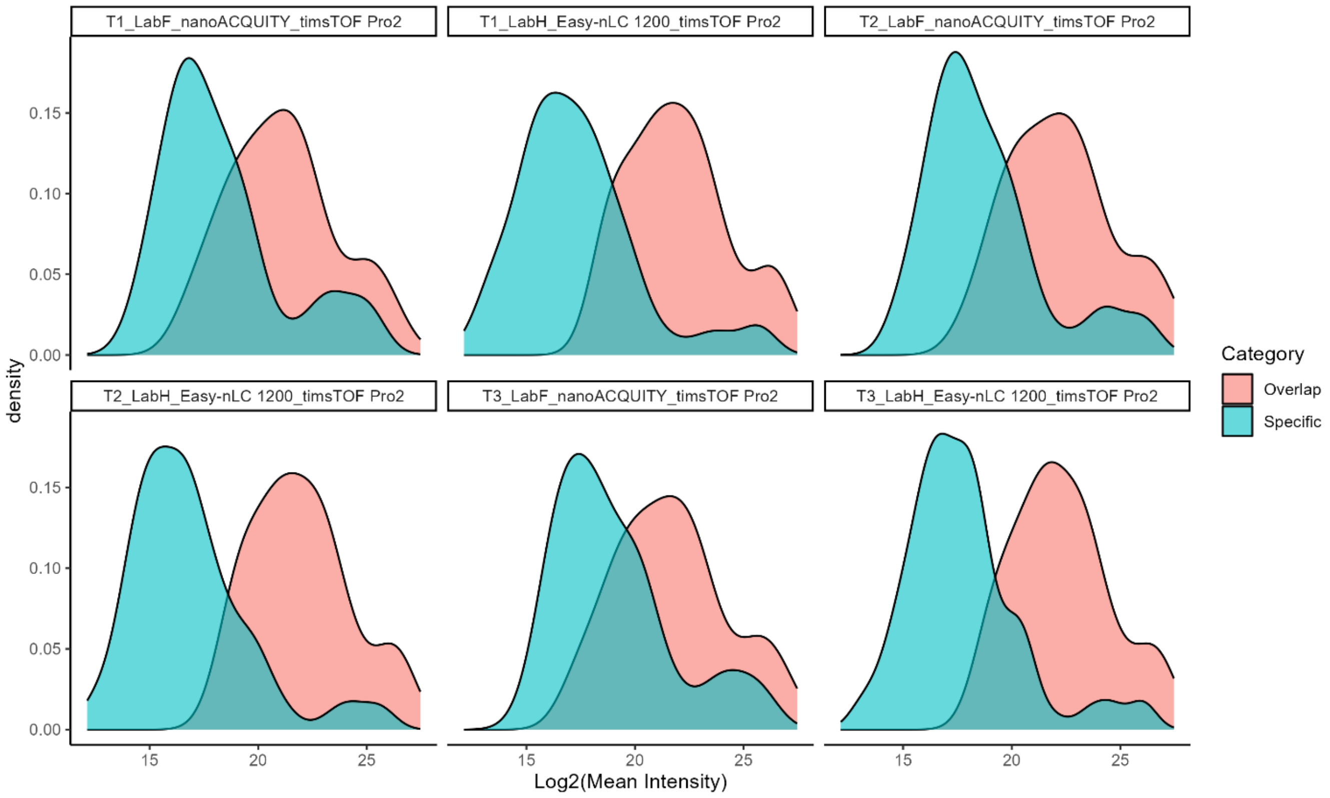

Fig. S70. Plasma sample intensity distributions for datasets measured on timsTOF Pro2 instruments. Highlighted in red are overlapping proteins and in blue dataset specific proteins.

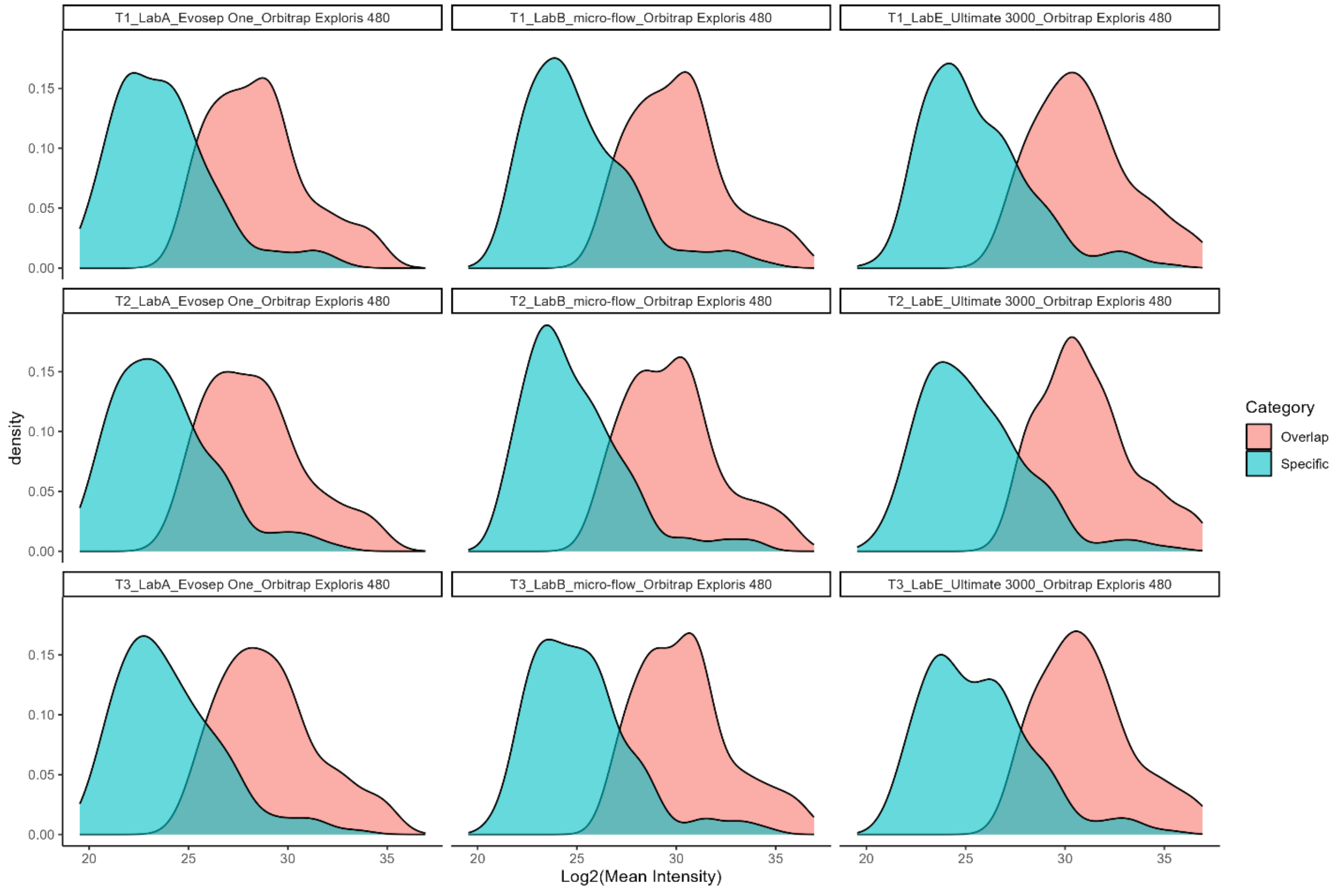

Fig. S71. Serum sample intensity distributions for datasets measured on Orbitrap Exploris 480 instruments. Highlighted in red are overlapping proteins and in blue dataset specific proteins.

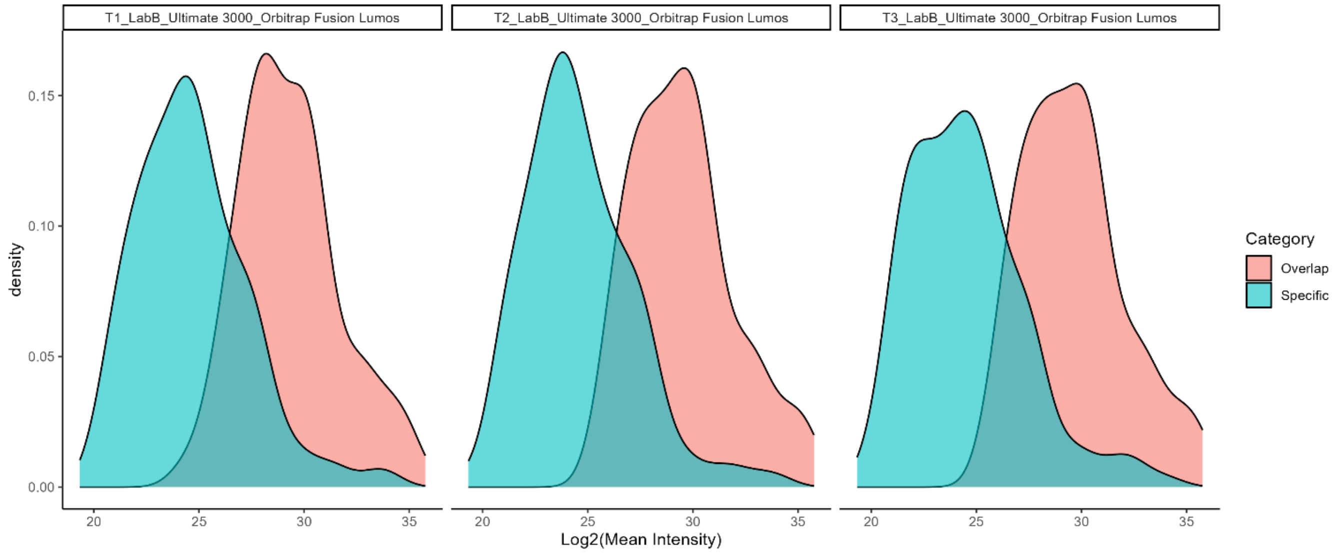

Fig. S72. Serum sample intensity distributions for datasets measured on Orbitrap Fusion Lumos instruments. Highlighted in red are overlapping proteins and in blue dataset specific proteins.

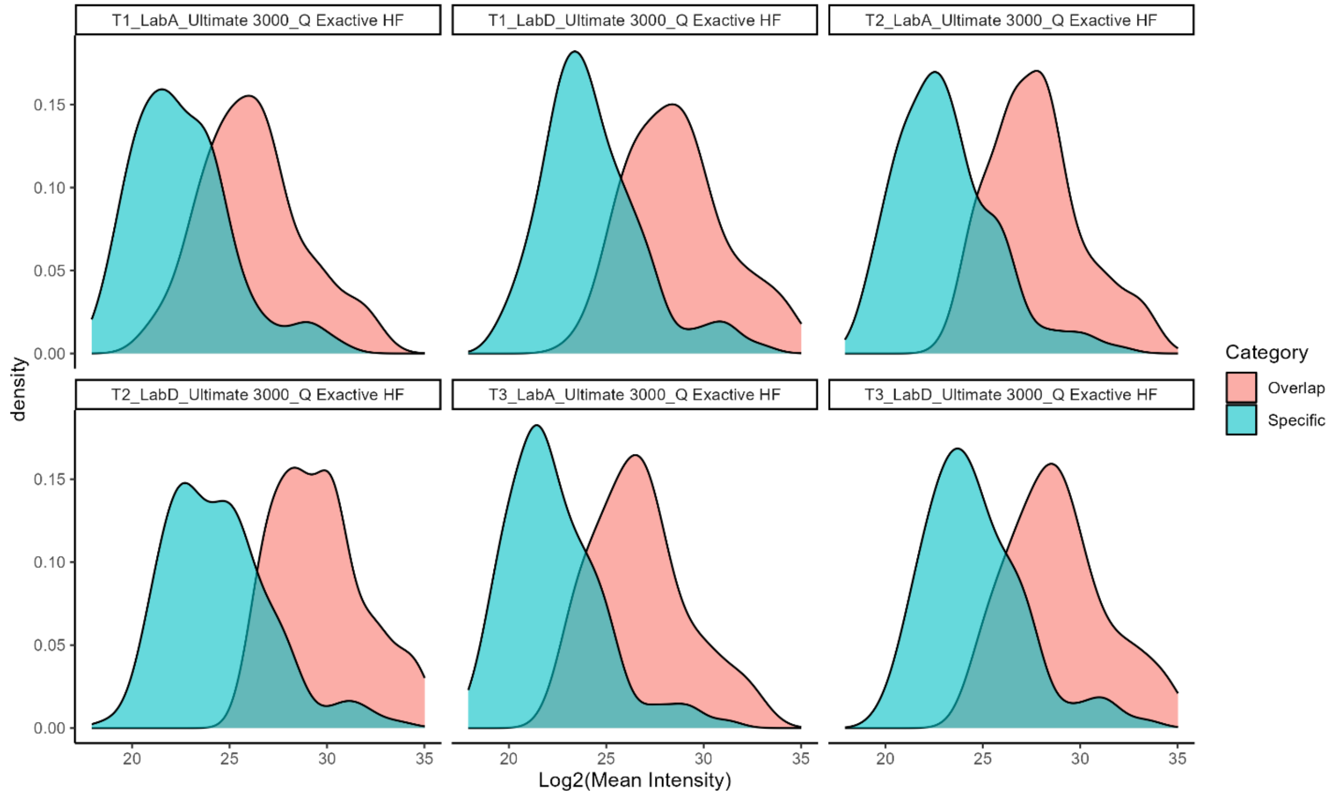

Fig. S73. Serum sample intensity distributions for datasets measured on Q Exactive HF instruments. Highlighted in red are overlapping proteins and in blue dataset specific proteins.

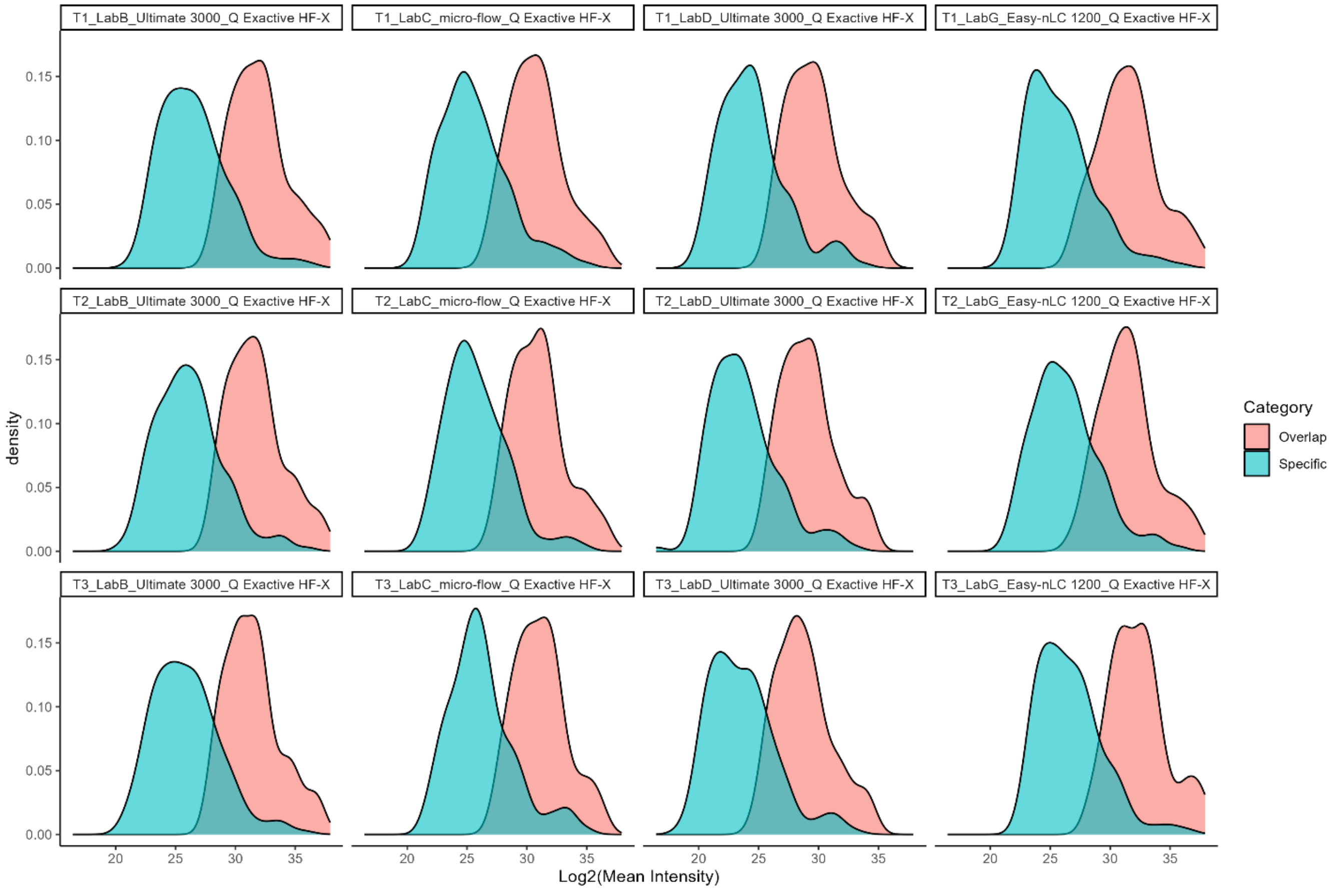

Fig. S74. Serum sample intensity distributions for datasets measured on Q Exactive HF-X instruments. Highlighted in red are overlapping proteins and in blue dataset specific proteins.

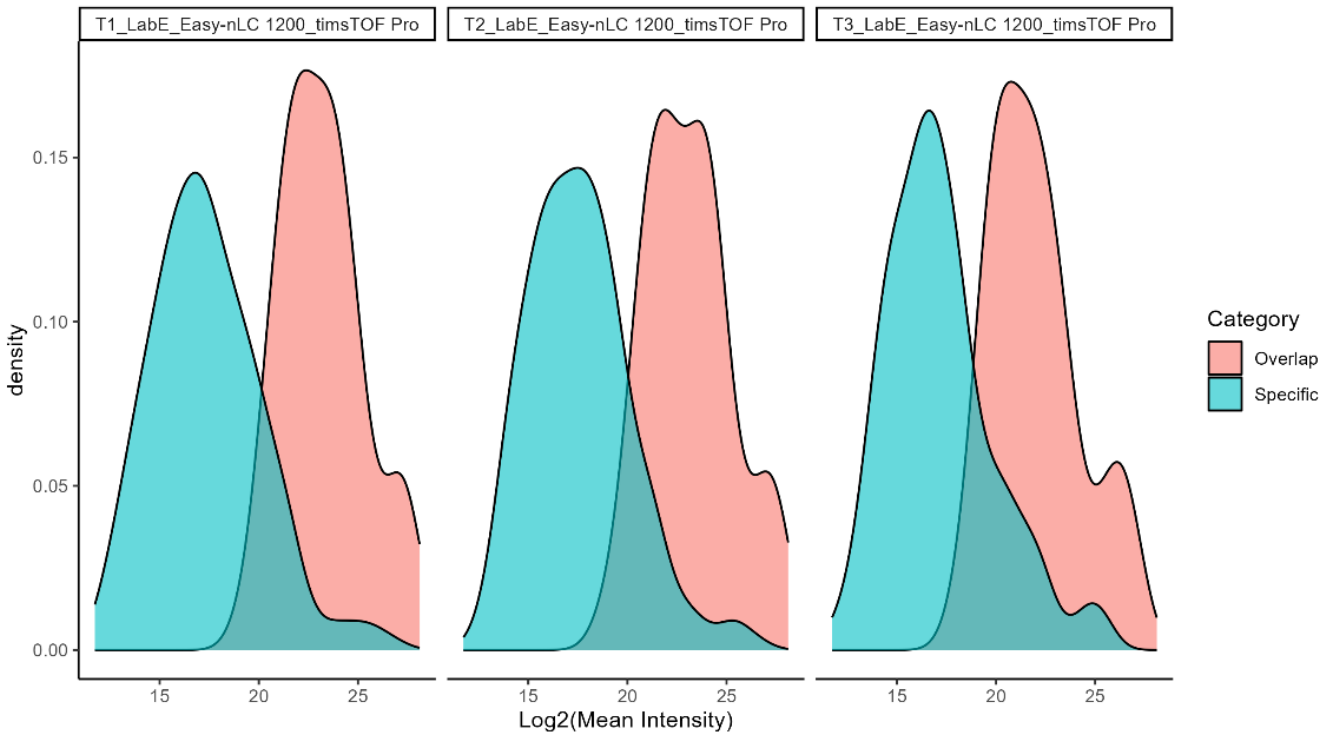

Fig. S75. Serum sample intensity distributions for datasets measured on timsTOF Pro instruments. Highlighted in red are overlapping proteins and in blue dataset specific proteins.

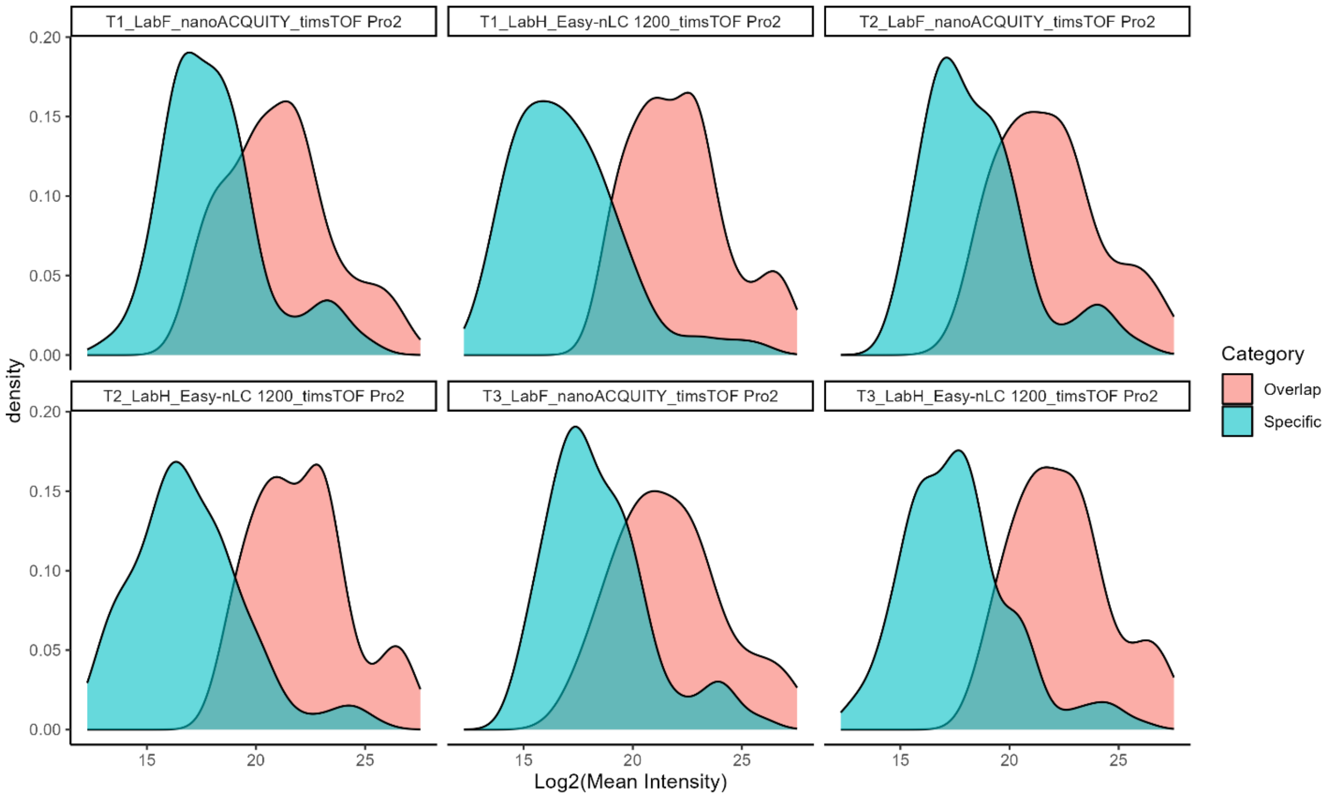

Fig. S76. Serum sample intensity distributions for datasets measured on timsTOF Pro2 instruments. Highlighted in red are overlapping proteins and in blue dataset specific proteins.

8. Overlap [%] – pairwise comparisons

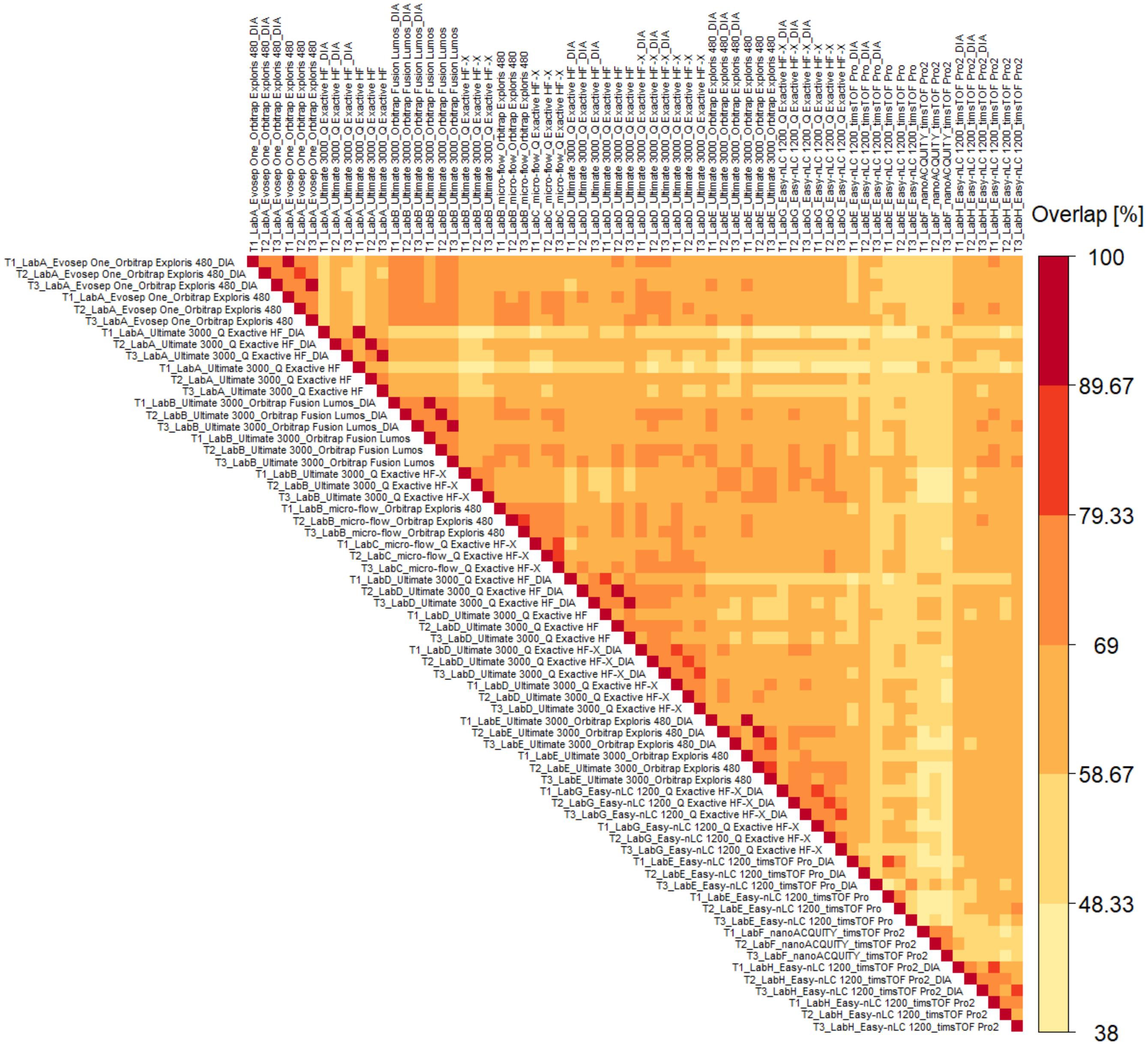

Fig. S77: Heatmap displaying the overlap [%] for each possible pairwise comparison between datasets on protein-level for plasma samples.

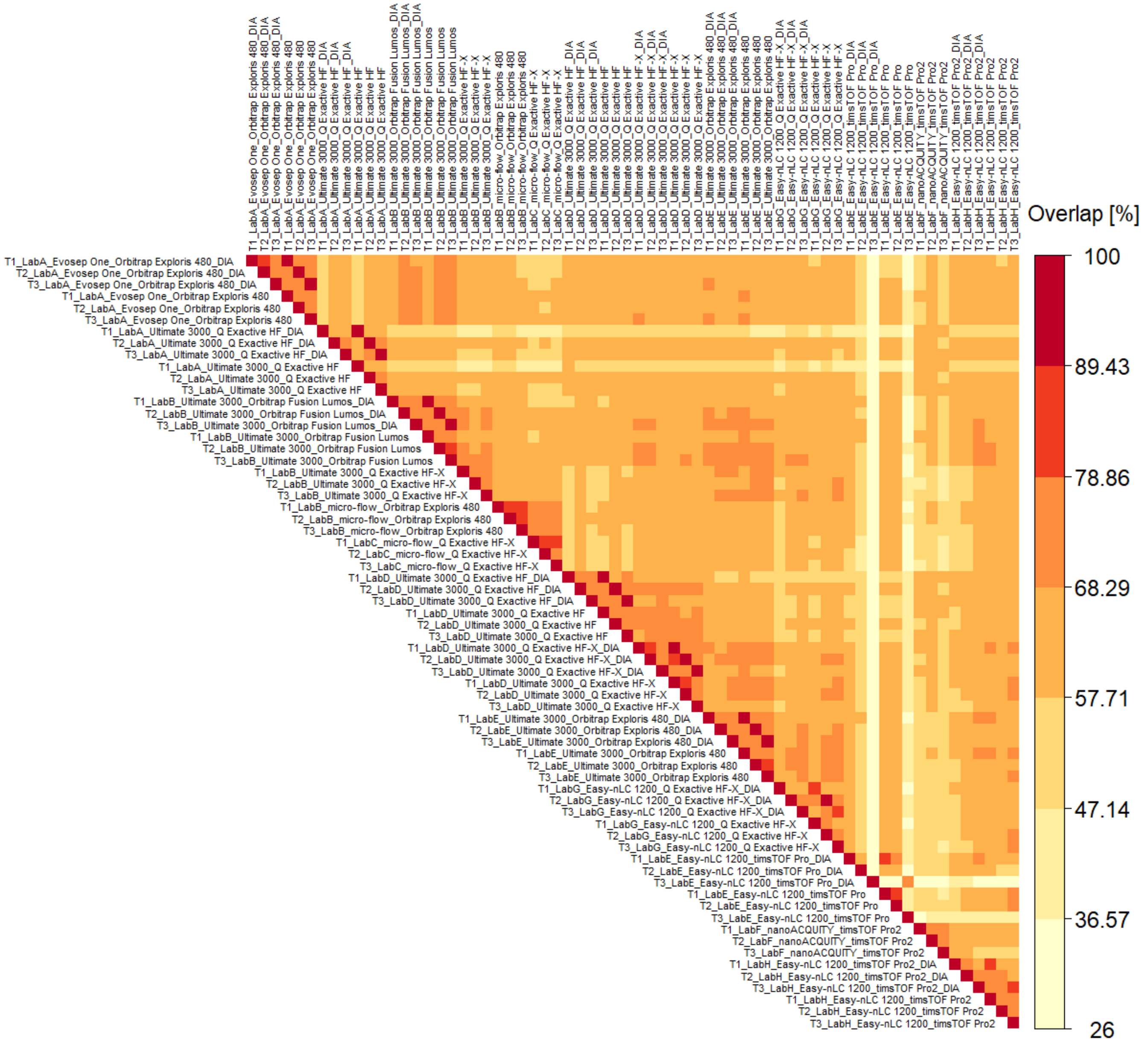

Fig. S78: Heatmap displaying the overlap [%] for each possible pairwise comparison between datasets on peptide-level for plasma samples.

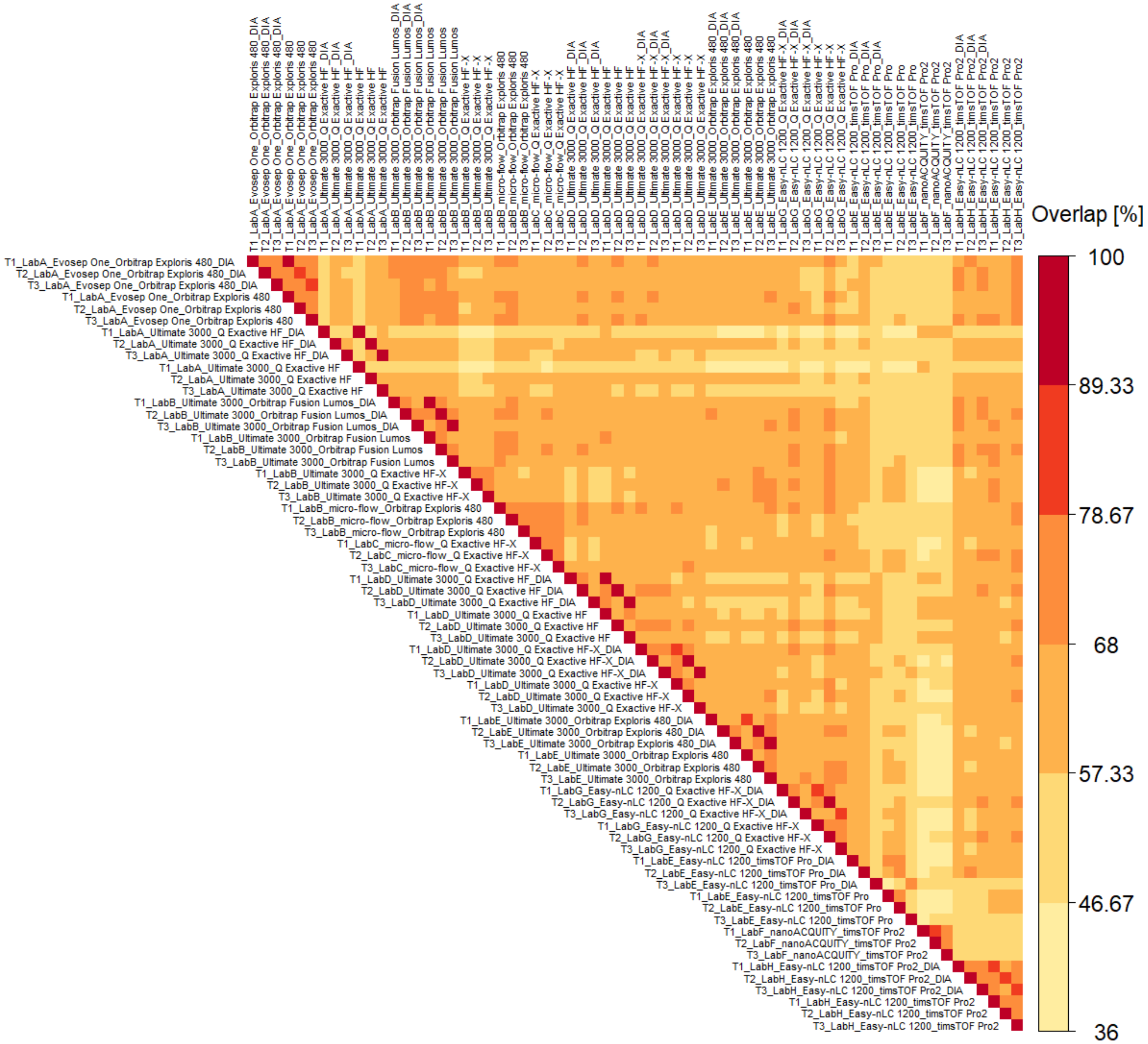

Fig. S79: Heatmap displaying the overlap [%] for each possible pairwise comparison between datasets on protein-level for serum samples.

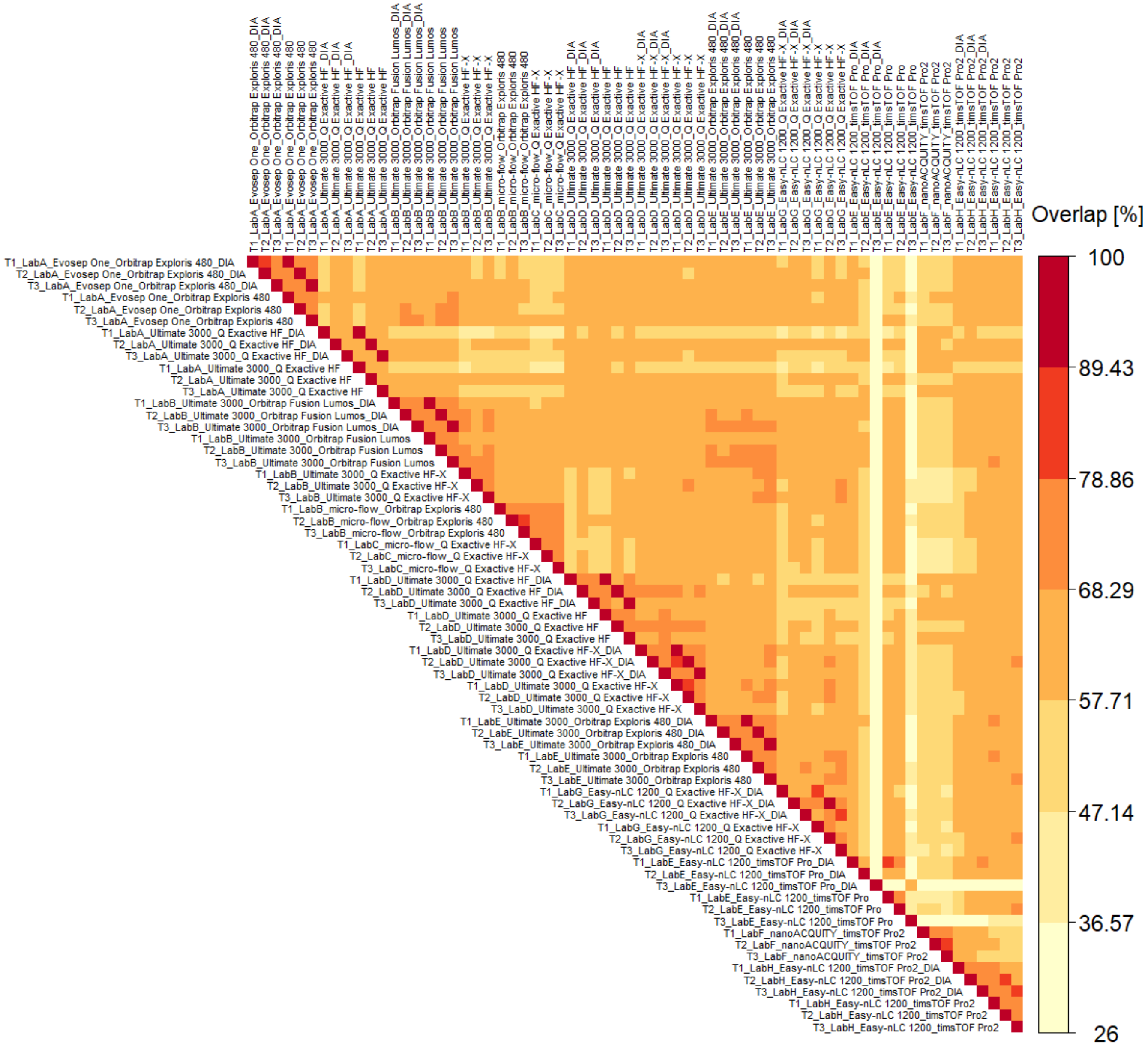

Fig. S80: Heatmap displaying the overlap [%] for each possible pairwise comparison between datasets on peptide-level for serum samples.

9. CV heatmap of common protein groups including all set ups

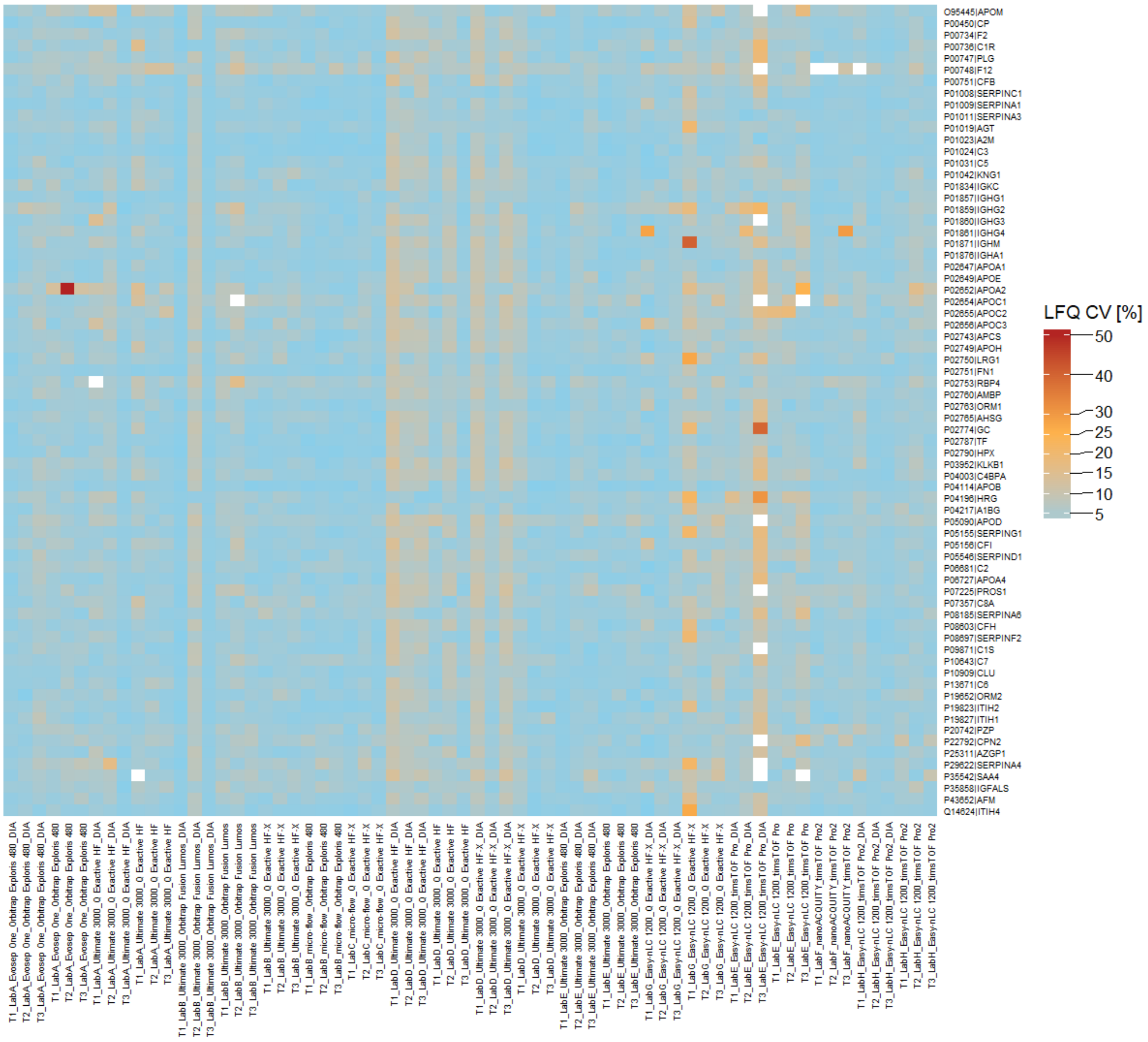

Fig. S81: Plasma - Heatmap displaying the LFQ CV [%] for commonly quantified protein groups across all set ups.

White squares refer to NA values where no LFQ values could be inferred and thus no CV was calculated.

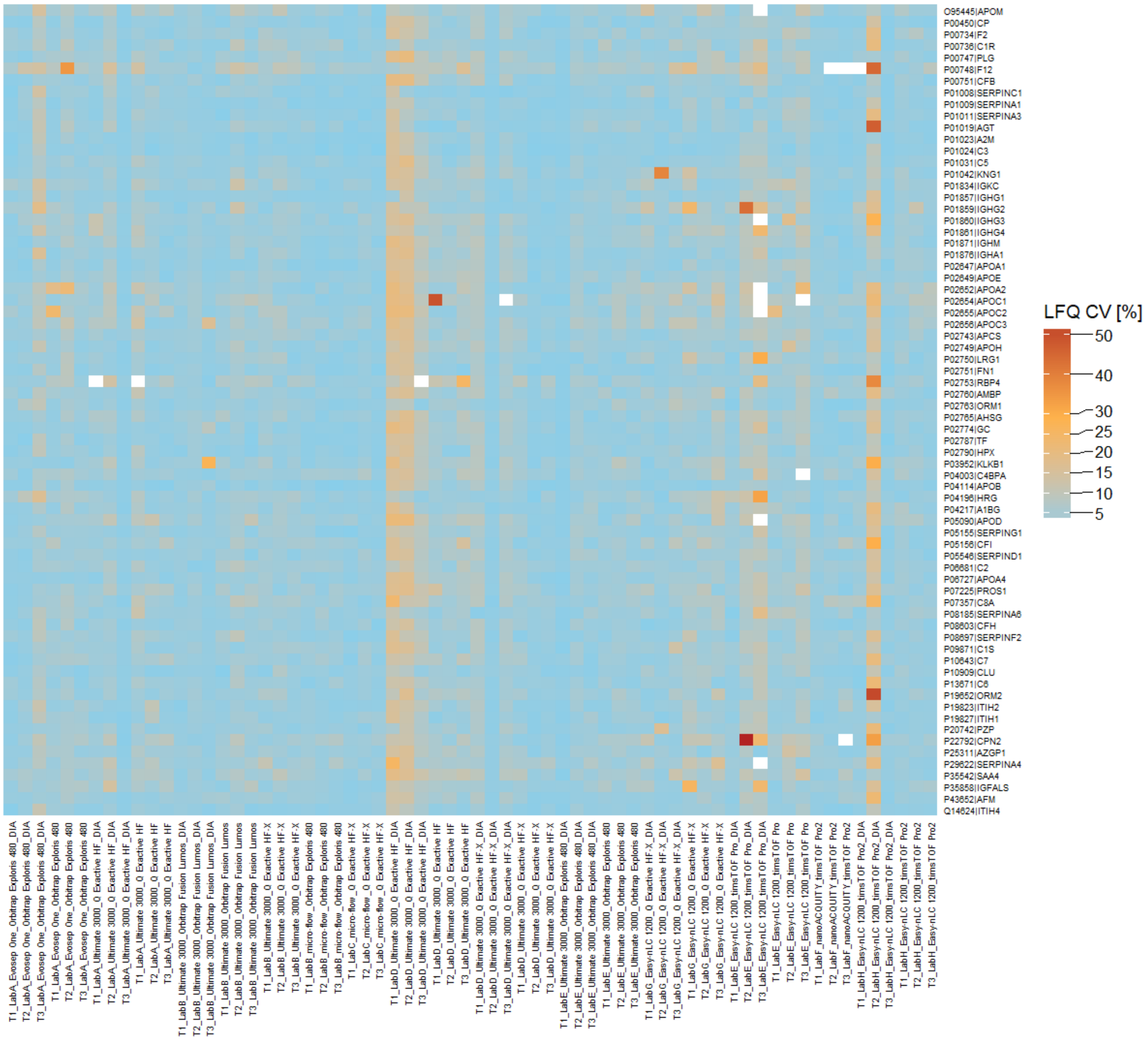

Fig. S82: Serum - Heatmap displaying the LFQ CV [%] for commonly quantified protein groups across all set ups. White squares refer to NA values where no LFQ values could be inferred and thus no CV was calculated.

10. DIA-NN Identifications

10.1 Protein-level

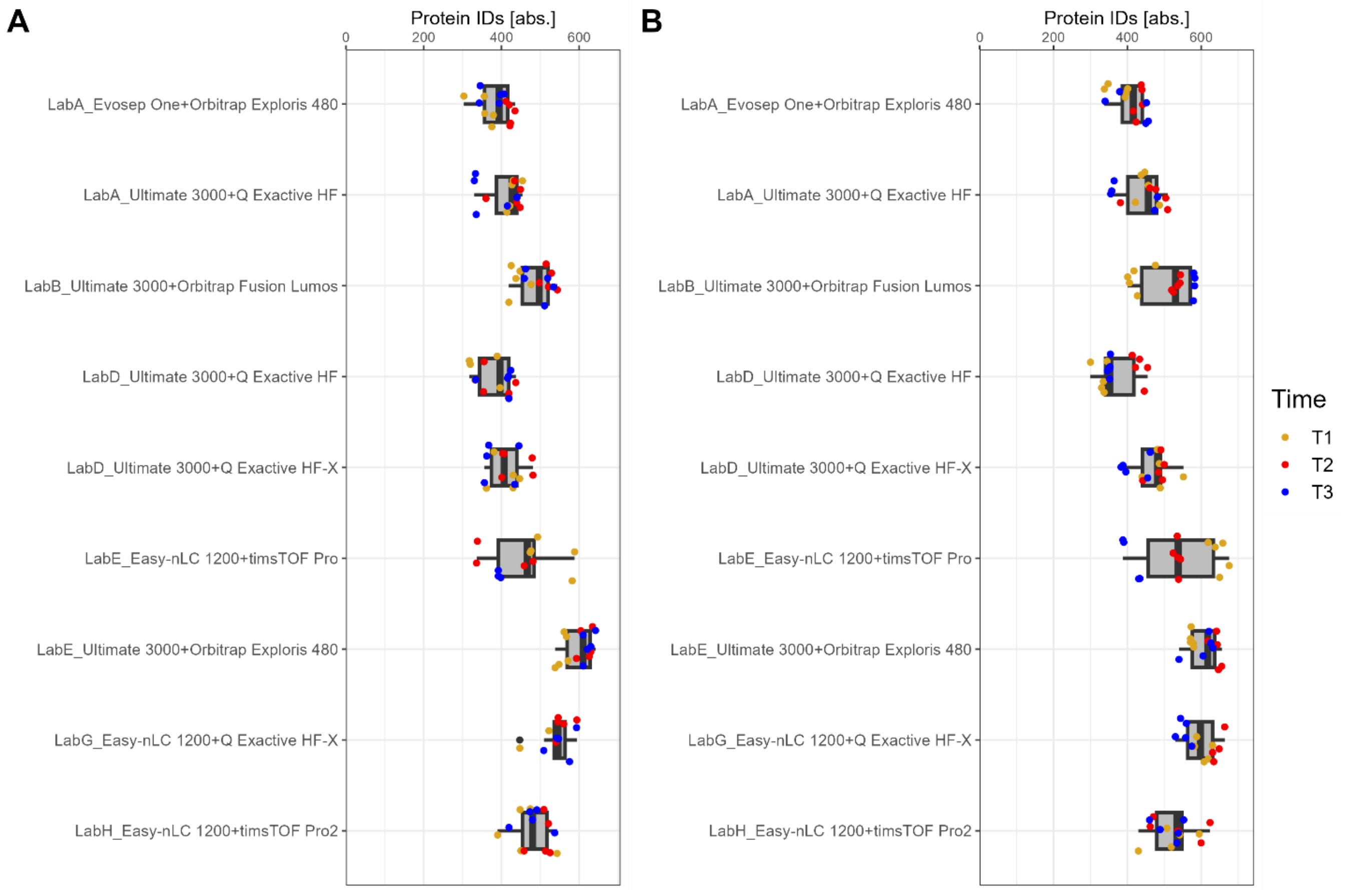

Fig. S83: Protein IDs for plasma (A) and serum (B) for different set ups measured in DIA mode. Measurements are color coded by timepoints. All DIA datasets were analyzed with DIA-NN (v.1.8.1).

11. Sample preparation

Serum and plasma samples were proteolysed using the iST-BCT kit from PreOmics (PreOmics GmbH, Martinsried, Germany) according to manufacturer's specifications. In short, the sample was reduced, alkylated and incubated for 3 hours at 37°C with Lys-C and trypsin. Resulting peptides (Serum: 73 µg/µL; Plasma: 78 µg/µL) were purified and evaporated after elution from the Cartridge. The peptide pellet was stored at -80°C until analysis.

12. LC-MS/MS settings

12.1 Laboratory A

Evosep One + Exploris 480 - DDA:

The peptide mixture, prepared as described in the instruction, was loaded on Evotips (one Evotip for each injection). They were placed in the Evosep (Evosep, Odense, Denmark) autosampler until analysis. The 30 samples per day method employing a 44 minutes gradient with solvents A (0.1% FA, H_2O) and B (0.1% FA, MeCN) was chosen and a 15 cm column (Dr. Maisch C18 AQ, 1.9µm beads, 150µm ID) used for separation of peptides. The LC was coupled to an Exploris 480 mass spectrometer (Thermo Fisher Scientific, Bremen, Germany). MS1 spectra were acquired in a Top 20 mode with a resolution of 120,000 in a mass range from 375-1500 m/z. The resolution for MS2 was 15,000, AGC target 100% and maximum injection time 40ms. Isolation width was 1.4 m/z.

Evosep One + Exploris 480 - DIA:

The peptide mixture, prepared as described in the instruction, was loaded on Evotips (one Evotip for each injection). They were placed in the Evosep (Evosep, Odense, Denmark) autosampler until analysis. The 30 samples per day method employing a 44 minutes gradient with solvents A (0.1%

FA, H_2O) and B (0.1% FA, MeCN) was chosen and a 15 cm column (Dr. Maisch C18 AQ, 1.9µm beads, 150µm ID) used for separation of peptides. The LC was coupled to an Exploris 480 mass spectrometer (Thermo Fisher Scientific, Bremen, Germany). Data independent acquisition was performed with a full scan with a resolution of 120000 from 400-1000 m/z, 100ms maximum injection time and a normalized AGC target of 300%. This scan was followed by DIA scans at a precursor mass range between 400-1000 m/z at an isolation window width of 10m/z with 1m/z overlap. Thus, the number of scan events was 60. The resolution was 30000 and AGC target 3000%.

UltiMate 3000 + QExactive HF - DDA:

The peptide solution was injected to an UltiMate 3000 RSLC nano-HPLC (Dionex, Germering, Germany) coupled to a Q Exactive HF mass spectrometer (Thermo Fisher Scientific, Bremen, Germany). Peptides were separated on a IonOpticks Odyssey column (25cm x 75µm, C18 1.6µm) at 40°C by a 50 minutes non-linear gradient at a flow rate of 300nL/min. Data were acquired in a data-dependent mode with a Top10 method. MS1 spectra were recorded at a resolution of 60,000 in a mass range from 375 to 1600 m/z. The resolution for MS2 was 15,000, AGC target 1e5 and maximum injection time 60ms. Isolation window was 2 m/z.

UltiMate 3000 + QExactive HF - DIA:

2µL of peptide solution were injected to an UltiMate 3000 RSLC nano-HPLC (Dionex, Germering, Germany) coupled to a Q Exactive HF mass spectrometer (Thermo Fisher Scientific, Bremen, Germany). Peptides were separated on a IonOpticks Odyssey column (25cm x 75µm, C18 1.6µm)

at 40°C by a 50 minutes non-linear gradient at a flow rate of 300nL/min. Data were acquired in a data-independent mode with a MS1 spectrum followed by 18 variable fragmentation windows. The MS1 spectrum was recorded at a resolution of 60000, maximum injection time of 60ms and AGC target of 3e6. The scan range was 350 to 1650 m/z. DIA was recorded at a resolution of 30000 with an AGC target of 3e6 and nCE of 27 with a default charge of 3 and variable isolation windows.

12.2 Laboratory B

Micro-flow + Exploris 480 – DDA:

Peptides were analyzed on a Dionex Ultimate 3000 RSLCnano CAP microflow system coupled to an Exploris mass spectrometer (ThermoFisher Scientific). 5 µg peptides were delivered to a column (Acclaim PepMap 100 C18, 150mm x 1mm, particle size 2 µm) at a flow rate of 50 µL/min and a temperature of 55 °C in HPLC grade water with 0.1% (v/v) formic acid and separated using a 30 min linear gradient from 3% to 28% of solvent B (0.1% (v/v) formic acid, 5% (v/v) DMSO in acetonitrile) nanoLC solvent A was 0.1% (v/v) formic acid, 5% (v/v) DMSO in HPLC grade water. The Exploris mass spectrometer was operated in data dependent acquisition (DDA) and positive ionization mode. MS1 spectra (360–1300 m/z) were recorded at a resolution of 60,000 using an automatic gain control (AGC) target value of 100% and maximum injection time (maxIT) of 50 msec. Only precursors with charge state 2 to 6 were selected and dynamic exclusion of 30 sec was enabled. Peptide fragmentation was performed using higher energy collision induced dissociation (HCD) and a normalized collision energy (NCE) of 28%. The precursor isolation window width was set to 1.1 m/z. MS2 Resolution was 15.000 with an automatic gain control (AGC) target value 100% and maximum injection time (maxIT) of 22 ms. The cycle time was set to 1s.

Dionex U3000 + Orbitrap Fusion LUMOS – DDA:

Peptides were analyzed on a Dionex Ultimate 3000 RSLCnano system coupled to a Fusion Lumos Tribrid mass spectrometer (ThermoFisher Scientific). 209 ng peptides were delivered to a trap column (ReproSil-pur C18-AQ, 5 µm, Dr. Maisch, 20 mm × 75 µm, self-packed) at a flow rate of 5 µL/min and a temperature of 50 °C in HPLC grade water with 0.1% (v/v) formic acid. After 10 minutes of loading, peptides were transferred to an analytical column (ReproSil Gold C18-AQ, 3 µm, Dr. Maisch, 450 mm × 75 µm, self-packed) and separated using a 60 min linear gradient from 4% to 32% of solvent B (0.1% (v/v) formic acid, 5% (v/v) DMSO in acetonitrile) at 300 nL/min flow rate. nanoLC solvent A was 0.1% (v/v) formic acid, 5% (v/v) DMSO in HPLC grade water. The Fusion Lumos mass spectrometer was operated in data dependent acquisition (DDA) and positive ionization mode. MS1 spectra (360–1300 m/z) were recorded at a resolution of 60,000 using an automatic gain control (AGC) target value of 4e5 and maximum injection time (maxIT) of 50 msec. Up to 20 peptide precursors were selected for fragmentation and Orbitrap readout. Only precursors with charge state 2 to 6 were selected and dynamic exclusion of 20 sec was enabled. Peptide fragmentation was performed using higher energy collision induced dissociation (HCD) and a normalized collision energy (NCE) of 30%. The precursor isolation window width was set to 1.3 m/z. MS2 Resolution was 15.000 with an automatic gain control (AGC) target value of 5e4 and maximum injection time (maxIT) of 22 ms.

Dionex U3000 + Fusion LUMOS – DIA:

Peptides were analyzed on a Dionex Ultimate 3000 RSLCnano system coupled to a Fusion Lumos Tribrid mass spectrometer (ThermoFisher Scientific). 209 ng peptides were delivered to a trap column (ReproSil-pur C18-AQ, 5 µm, Dr. Maisch, 20 mm × 75 µm, self-packed) at a flow rate of

5 μL/min and a temperature of 50 °C in HPLC grade water with 0.1% (v/v) formic acid. After 10 minutes of loading, peptides were transferred to an analytical column (ReproSil Gold C18-AQ, 3 μm, Dr. Maisch, 450 mm × 75 μm, self-packed) and separated using a 60 min linear gradient from 4% to 32% of solvent B (0.1% (v/v) formic acid, 5% (v/v) DMSO in acetonitrile) at 300 nL/min flow rate. nanoLC solvent A was 0.1% (v/v) formic acid, 5% (v/v) DMSO in HPLC grade water. The Fusion Lumos mass spectrometer was operated in data independent acquisition (DIA) and positive ionization mode. The DIA method consisted of a survey scan from 360 to 1300 m/z at 120,000 resolution and an automatic gain control (AGC) target value of 1e6 or 50 ms maximum injection time. The MS2 scans range was set to 350 – 1150 m/z with 41 variable windows at a resolution of 30,000, 1 m/z overlap between scan windows and an automatic gain control (AGC) target of 5e5 or 54 ms maximum injection time. Fragmentation was performed using higher energy collision induced dissociation (HCD) and a normalized collision energy (NCE) of 30%.

Dionex U3000 + Q-Exactive HFX – DDA:

Peptides were analyzed on a Dionex Ultimate 3000 RSLCnano system coupled to a Q-Exactive HF-X mass spectrometer (ThermoFisher Scientific). 209 ng peptides were delivered to a trap column (ReproSil-pur C18-AQ, 5 μm, Dr. Maisch, 20 mm × 75 μm, self-packed) at a flow rate of 5 μL/min and a temperature of 50 °C in HPLC grade water with 0.1% (v/v) formic acid. After 10 minutes of loading, peptides were transferred to an analytical column (ReproSil Gold C18-AQ, 3 μm, Dr. Maisch, 450 mm × 75 μm, self-packed) and separated using a 60 min linear gradient from 4% to 32% of solvent B (0.1% (v/v) formic acid, 5% (v/v) DMSO in acetonitrile) at 300 nL/min flow rate. nanoLC solvent A was 0.1% (v/v) formic acid, 5% (v/v) DMSO in HPLC grade water. The Q-Exactive HF-X mass spectrometer was operated in data dependent acquisition (DDA) and

positive ionization mode. MS1 spectra (360–1300 m/z) were recorded at a resolution of 60,000 using an automatic gain control (AGC) target value of 3e6 and maximum injection time (maxIT) of 45 msec. Up to 18 peptide precursors were selected for fragmentation. Only precursors with charge state 2 to 6 were selected and dynamic exclusion of 25 sec was enabled. Peptide fragmentation was performed using higher energy collision induced dissociation (HCD) and a normalized collision energy (NCE) of 26%. The precursor isolation window width was set to 1.3 m/z. MS2 Resolution was 15.000 with an automatic gain control (AGC) target value of 1e5 and maximum injection time (maxIT) of 25 ms.

12.3 Laboratory C

<u>Micro-flow + Q-Exactive HFX – DDA:</u>

Plasma and serum samples were analyzed on a micro-flow LC-MS/MS system using a modified Vanquish pump (Thermo Fisher Scientific) coupled to a Q Exactive Orbitrap HF-X mass spectrometer (Thermo Fisher Scientific). For each replicate 5 µg of peptides were injected. Chromatographic separation was performed via direct sample injection onto the head of a 15 cm Acclaim PepMap 100 C18 column (2 µm particle size, 1 mm ID, Thermo Fisher Scientific) at a flow rate of 50 µL/min. Samples were separated using a 30 min gradient ranging from 3% to 28% B (.1% FA, 3% DMSO in ACN) in solvent A (0.1% FA, 3% DMSO in water) at a flow rate of 50 µL/min and a temperature of 55 °C. The HF-X was operated in positive ion mode, using an electrospray voltage of 4.0 kV, a funnel RF lens value of 40, capillary temperature of 320°C and an auxillary gas heater temperature of 200°C. The flow rates for sheath gas, aux gas and sweep gas were set to 35, 5 and 0. MS1 spectra were acquired over a mass-to-charge (m/z) range of 360-1300 m/z at a resolution of 120,000 in the Orbitrap using a maximum injection time of 50 ms and an

automatic gain control (AGC) target value of 3e6. Up to 12 peptide precursors were isolated (isolation width of 1.3 m/z, maximum injection time of 22 ms, AGC value of 1e5), fragmented by HCD using 28% normalized collision energy (NCE) and analyzed in the Orbitrap at a resolution of 15,000. The dynamic exclusion duration of fragmented precursor ions was set to 30 s.

12.4 Laboratory D

<u>Ultimate 3000 + Q Exactive HF – DDA:</u>

The peptide mixture was injected to an UltiMate 3000 RSLC nano-HPLC (Dionex, Germering, Germany) coupled to a Q Exactive HF mass spectrometer (Thermo Fisher Scientific, Bremen, Germany). Peptides were separated on a nanoEase M/Z HSS T3 column (25cm x 75µm, C18 1.8µm, 100Å) at 40°C by a 80 minutes non-linear gradient at a flow rate of 250 nL/min ranging from 3% to 25% B (0.1% FA, H_2O) in A (0.1% FA, 2% MeCN, 97.9% H_2O). Data were acquired in a data-dependent mode with a Top10 method. MS1 spectra were recorded at a resolution of 60000 in a mass range from 300 to 1500 m/z. The resolution for MS2 was 15000, AGC target 1e5 and maximum injection time 50 ms. Isolation window was 1.6 m/z.

<u>Ultimate 3000 + Q Exactive HF – DIA:</u>

The peptide mixture was injected to an UltiMate 3000 RSLC nano-HPLC (Dionex, Germering, Germany) coupled to a Q Exactive HF mass spectrometer (Thermo Fisher Scientific, Bremen, Germany). Peptides were separated on a nanoEase M/Z HSS T3 column (25cm x 75µm, C18 1.8µm, 100Å) at 40°C by a 90 minutes non-linear gradient at a flow rate of 250nL/min ranging from 3% to 25% B (0.1% FA, H_2O) in A (0.1% FA, 2% MeCN, 97.9% H_2O). Data were acquired in a data-independent mode with a MS1 spectrum followed by 37 fragmentation windows. The

MS1 spectrum was recorded at a resolution of 120000, maximum injection time of 120 ms and AGC target of 3e6. The scan range was 300 to 1650 m/z. DIA was recorded at a resolution of 30000 with an AGC target of 3e6 and variable isolation width.

Ultimate 3000 + Q Exactive HFX – DDA:

The peptide mixture was injected to an UltiMate 3000 RSLC nano-HPLC (Dionex, Germering, Germany) coupled to a Q Exactive HFX mass spectrometer (Thermo Fisher Scientific, Bremen, Germany). Peptides were separated on a nanoEase M/Z HSS T3 column (25cm x 75µm, C18 1.8µm, 100Å) at 40°C by a 80 minutes non-linear gradient at a flow rate of 250nL/min ranging from 3% to 25% B (0.1% FA, H_2O) in A (0.1% FA, 2% MeCN, 97.9% H_2O). Data were acquired in a data-dependent mode with a Top15 method. MS1 spectra were recorded at a resolution of 60000 in a mass range from 300 to 1500 m/z. The resolution for MS2 was 15000, AGC target 1e5 and maximum injection time 50 ms. Isolation window was 1.6 m/z.

Ultimate 3000 + Q Exactive HFX – DIA:

The peptide mixture was injected to an UltiMate 3000 RSLC nano-HPLC (Dionex, Germering, Germany) coupled to a Q Exactive HFX mass spectrometer (Thermo Fisher Scientific, Bremen, Germany). Peptides were separated on a nanoEase M/Z HSS T3 column (25cm x 75µm, C18 1.8µm, 100Å) at 40°C by a 90 minutes non-linear gradient at a flow rate of 250nL/min ranging from 3% to 25% B (0.1% FA, H_2O) in A (0.1% FA, 2% MeCN, 97.9% H_2O). Data were acquired in a data-independent mode with a MS1 spectrum followed by 37 fragmentation windows. The MS1 spectrum was recorded at a resolution of 120000, maximum injection time of 120 ms and

AGC target of 3e6. The scan range was 300 to 1650 m/z. DIA was recorded at a resolution of 30000 with an AGC target of 3e6 and variable isolation width.

12.5 Laboratory E

Easy nLC 1200 + timsTOF Pro – DDA:

For the timsTOF Pro PASEF acquisition of plasma/serum ring trial samples, peptides were separated using the Easy nLC 1200 system fitted with an analytical column (Aurora Ultimate Series Emitter Column with CSI fitting, C18, 1.6 µm, 75 µm x 25 cm) (Ion Opticks). The outlet of the analytical column with a captive spray fitting was directly coupled to a timsTOF Pro (Bruker) mass spectrometer using a captive spray source. Solvent A was ddH2O (Biosolve Chimie), 0.1% (v/v) FA (Biosolve Chimie), 2% acetonitrile (ACN) (Pierce, Thermo Scientific), and solvent B was 100% ACN in dH2O, 0.1% (v/v) FA. The samples were loaded at a constant pressure. Peptides were eluted via the analytical column at a constant flow of 0.4 µL per minute at 55°C. During the elution, the percentage of solvent B was increased in a linear fashion from 4 to 17% in 30 minutes, then from 17 to 29% in 15 minutes, then from 29 to 37.5% in a further 5 minutes, and then to 100% in 10 minutes. Finally, the gradient was finished with 2.5 minutes at 4% solvent B to equilibrate the column. Peptides were introduced into the mass spectrometer via the standard Bruker captive spray source at default settings. The glass capillary was operated at 3500 V with 500 V end plate offset and 3 L/minute dry gas at 180°C. Full scan MS spectra with mass range m/z 100 to 1700 and a 1/k0 range from 0.85 to 1.3 V*s/cm2 with 100 ms ramp time were acquired with a rolling average switched on (10x). The duty cycle was locked at 100%, the ion polarity was set to positive, and the TIMS mode was enabled. The active exclusion window was set to 0.015 m/z, 1/k0 0.015 V*s/ cm2.

The isolation width was set to mass 700-800 m/z, width 2 – 3 m/z and the collision energy to 1/k0 0.85-1.3 V*s/ cm2, energy 27- 45 eV.

Easy nLC 1200 + timsTOF Pro – DIA

For the DIA acquisition of plasma/serum ring trial samples, gradient and acquisition method settings were the same as described for the DDA runs unless otherwise stated. For the DIA scans, a variable window pattern was used, covering the precursor range of 100 to 1700 m/z.

Ultimate 3000 + Exploris 480 – DDA:

For the Exploris 480 data acquisition, peptides were separated using the Ultimate 3000 LC fitted with a 75 µm x 25 cm analytical column (nanoEase MZ BEH C18, 1.7 µm, 130 Å, Waters). The outlet of the analytical column was coupled directly to an Exploris 480 Orbitrap (Thermo Fisher Scientific) mass spectrometer. Solvent A was ddH_2O (Biosolve Chimie), 0.1% (v/v) FA (Biosolve Chimie) and solvent B was 80% acetonitrile (ACN, Pierce – Thermo Scientific) in ddH_2O, 0.1% (v/v) FA. The samples were loaded with a constant flow of solvent A at a constant pressure. Peptides were eluted via the analytical column at a constant flow of 0.3 µL/minute at 35°C. During the elution, the percentage of solvent B was constant for 3 minutes at 2% before increasing in a linear fashion from 2 to 5% in 1 minute, then from 5% to 30% in 43 minutes, and then from 30% to 80% in a further 1 minute. Finally, the gradient was finished with 1 minute at 80% solvent B, followed by 1 minute 98% solvent A. Peptides were introduced into the mass spectrometer via a Pico-Tip Emitter 360 µm OD x 20 µm ID; 10 µm tip (New Objective) and at a static spray voltage of 2.2 kV in positive ion mode. The capillary temperature was set at 275°C. Full scan MS spectra

with mass range m/z 380 to 1400 were acquired in the Orbitrap with a resolution of 60,000 FWHM. The filling time was set to a maximum of 45 ms with a normalized automatic gain control (AGC) target of 300%. The intensity threshold was set to 1e5. Charge states were set to 2-6 and dynamic exclusion was left at default settings with exclusion after 1 times, exclusion after 20 seconds, and a mass tolerance of plus and minus 10 pp. Isotopes were excluded. For MS2 scans, the resolution was set to 15,000 FWHM with a normalized automatic gain control target of 100% and maximum fill time of 22 ms. The isolation window was set to 1.4 m/z. The collision energy mode was fixed with a HCD collision energy of 26%.

Ultimate 3000 + Exploris 480 – DIA:

For the DIA acquisition of plasma/serum ring trial samples, gradient and acquisition method settings were the same as described for the DDA runs unless otherwise stated. The MSMS settings were set as follows: Isolation offset was set to off, the collision energy mode was fixed with a HCD collision energy of 28%. The Orbitrap resolution was set to 30,000 with a normalized automatic gain control target of 100%. The maximum injection time was set to 54 ms. For the DIA scans, a variable window pattern was used, covering the precursor range of 350 to 1400 m/z at a resolution of 120,000.

12.6 Laboratory F

nanoAcquity + timsTOF Pro2 – DDA:

Samples were analyzed on a timsTOF-Pro2 (Bruker Daltonics) coupled online to a nanoAcquity LC system (Waters). Peptides were directly injected onto an Aurora 5 cm x 150 µm analytical emitter column (1.6 µm C18, IonOpticks, Australia) at a temperature of 25 °C and a flow rate of 2

µl/min for loading, washing and equilibration, and 1 µL/min for the separation of peptides running a linear gradient from 0 to 34% B (mobile phase A = 0.1% FA in water, mobile phase B = 0.1% FA in ACN). DDA-PASEF was programmed using a 50 ms TIMS ramp, from 0.6 to 1.4 1/k0, acquiring ions in the TOF from m/z 100 to 1700, with a cycle time of 0.62 seconds. The quadrupole isolation width was set to 2 m/z for m/z < 700 and to 3 m/z for m/z of 800 or greater.

12.7 Laboratory G

Easy nLC 1200 + QExactive HF-X– DDA:

Samples were measured by LC-MS using orbitrap Exploris 480 (Thermo Fisher Scientific) or Q Exactive HF-X (Thermo Fisher Scientific) instrumentation coupled to EASY nLC 1200 systems (Thermo Fisher Scientific). Peptide samples were separated using a 44-minute LC method on an in-house packed analytical column (75 µm x 20 cm, C18-AQ, 1.9 µm beads; Dr. Maisch Reprosil-Pur 120). The mobile phases consisted of solvent A (3% ACN, 0.1% formic acid) and solvent B (90% ACN, 0.1% formic acid). The gradient started at 2% solvent B for 1 minute, increased to 20% B over 19 minutes, and further to 30% B over the next 9 minutes. Solvent B was then ramped to 60% over 3 minutes, followed by a rapid increase to 90% B within 1 minute. The system was washed at 90% B for 5 minutes, reduced to 50% B over 1 minute, and re-equilibrated at 50% B for 5 minutes. A constant flow rate of 250 nL/min was maintained throughout the run. Full scans were acquired at a resolution of 60,000 over a scan range of 350–1800 m/z. Charge states were filtered to include ions with charges from 2 to 6, and dynamic exclusion was applied for 20 seconds with a 10 ppm mass tolerance. the full scan AGC target was set to 3×10^6, with a maximum injection time of 10 ms. Data types were set to profile for MS1 scans and centroid for MS2 scans. MS2 scans were performed using a top-20 data-dependent acquisition approach, and the intensity threshold for MS2 was set to 5×10^4. MS2 scans were acquired at a resolution of 15,000 with an isolation width of 1.3 m/z, a normalized AGC target of 100%, and a maximum injection time of 22 ms.

Easy nLC 1200 + QExactive HF-X– DIA:

Samples were measured by LC-MS using orbitrap Exploris 480 (Thermo Fisher Scientific) or Q Exactive HF-X (Thermo Fisher Scientific) instrumentation coupled to EASY nLC 1200 systems (Thermo Fisher Scientific). Peptide samples were separated using a 44-minute LC method on an in-house packed analytical column (75 µm x 20 cm, C18-AQ, 1.9 µm beads; Dr. Maisch Reprosil-Pur 120). The mobile phases consisted of solvent A (3% ACN, 0.1% formic acid) and solvent B (90% ACN, 0.1% formic acid). The gradient started at 2% solvent B for 1 minute, increased to 20% B over 19 minutes, and further to 30% B over the next 9 minutes. Solvent B was then ramped to 60% over 3 minutes, followed by a rapid increase to 90% B within 1 minute. The system was washed at 90% B for 5 minutes, reduced to 50% B over 1 minute, and re-equilibrated at 50% B for 5 minutes. A constant flow rate of 250 nL/min was maintained throughout the run. Full scans were acquired over a scan range of 350 to 1650 m/z at a resolution of 120,000. The DIA method utilized 40 overlapping isolation windows spanning the m/z range of 350 to 1650. The window widths ranged from 14 to 440 m/z, with narrower windows (14–20 m/z) applied at lower m/z values for higher resolution and progressively wider windows (up to 440 m/z) at higher m/z values for efficient coverage. Window placement was centered at intervals from 375 to 1430 m/z, with overlapping boundaries to ensure comprehensive sampling of precursor ions across the mass range (see table). The AGC target for full scans was set to 3×10^6 ions with a maximum injection time of 60 ms. MS2 scans were performed in 40 sub-experiments using variable isolation window sizes identical to those applied on the Exploris system. The resolution for MS2 scans was set to 30,000, the AGC target to 3e6 ions, and the maximum injection time to 35 ms. Stepped collision energies of 25.5, 27, and 30 were applied, and the first mass was set to 200 m/z.

Table S3: Measurement scheme.

Center m/z	Isolation width (m/z)	Item	From m/z	To m/z	Delta m/z
375	50	1	350	400	50
408	18	2	399	417	18
424	16	3	416	432	16
439	16	4	431	447	16
453.5	15	5	446	461	15
467.5	15	6	460	475	15
481	14	7	474	488	14
494	15	8	486.5	501.5	15
507.5	15	9	500	515	15
521	14	10	514	528	14
534	14	11	527	541	14
547	14	12	540	554	14
560	14	13	553	567	14
573	14	14	566	580	14
586.5	15	15	579	594	15
600	14	16	593	607	14
613.5	15	17	606	621	15
627.5	15	18	620	635	15
641.5	15	19	634	649	15
655.5	15	20	648	663	15
669.5	15	21	662	677	15
684	16	22	676	692	16
699	16	23	691	707	16
714.5	17	24	706	723	17
730.5	17	25	722	739	17
747	18	26	738	756	18
764.5	19	27	755	774	19
783	20	28	773	793	20
802	20	29	792	812	20
822	22	30	811	833	22
843.5	23	31	832	855	23
866.5	25	32	854	879	25
891	26	33	878	904	26
918	30	34	903	933	30
948.5	33	35	932	965	33
984	40	36	964	1004	40
1027	48	37	1003	1051	48
1081.5	63	38	1050	1113	63

1161.5	99	39	1112	1211	99
1430	440	40	1210	1650	440

12.8 Laboratory H

Easy nLC 1200 + timsTOF Pro2– DDA:

The samples were measured on an EASY-nLC 1200 system (Thermo Fisher Scientific) coupled to a TIMS quadrupole TOF mass spectrometer (Bruker timsTOF Pro2). Peptides were separated in 32 min gradient ranging from 2-29% B (0.1% FA, 9.9% H_2O, 90% MeCN) in A (0.1% FA, 3% MeCN, 96.9% H_2O) at a flow rate of 250 nl/min on a home-packed column (20 cm × 75 µm i.d., 1.9 µm ReproSil-Pur C18-AQ particles from Dr. Maisch) at 50 °C. The Bruker default method was used for ddaPASEF runs (DDA PASEF standard 1.1sec cycle time) based on 10 PASEF scans per 100ms TIMS cycle. A duty cycle of 100% was achieved by setting the ramp time and accumulation time to 100ms each. Singly charged precursors were excluded by their position in the m/z–ion mobility plane. Ion mobility range was set to 0.6 -1.6 1/K0. The quadrupole isolation width was set to 2 m/z for m/z < 700 and to 3 m/z for m/z of 800 or greater. Scan range was set to m/z 100 to 1,700 and active exclusion to 0.4 min. Threshold intensity for MS2 triggering was 2,500 at a target intensity of 20,000. Collision energy settings were default (20 eV for 0.6 1/K0 and 59 for 1.6 1/K0).

Easy nLC 1200 + timsTOF Pro2– DIA:

The samples were measured on an EASY-nLC 1200 system (Thermo Fisher Scientific) coupled to a TIMS quadrupole TOF mass spectrometer (Bruker timsTOF Pro2). Peptides were separated in 32 min gradient at a flow rate of 250 nl/min on a home-packed column (20 cm × 75 µm i.d., 1.9 µm ReproSil-Pur C18-AQ particles from Dr. Maisch) at 50 °C. To perform diaPASEF, the default method from Bruker was used ('diaPASEF – long gradient') with a scan range of m/z 100 to 1,700

and mobility range of 0.6 -1.6 1/K0. A duty cycle of 100% was achieved by setting the ramp time and accumulation time to 100 ms each. The quadrupole isolation width was set to 2 m/z for m/z < 700 and to 3 m/z for m/z of 800 or greater.
